# New Butenolides and Cyclopentenones from Saline Soil-Derived Fungus *Aspergillus Sclerotiorum*

**DOI:** 10.3390/molecules24142642

**Published:** 2019-07-21

**Authors:** Li-Ying Ma, Huai-Bin Zhang, Hui-Hui Kang, Mei-Jia Zhong, De-Sheng Liu, Hong Ren, Wei-Zhong Liu

**Affiliations:** 1 College of Pharmacy, Binzhou Medical University, Yantai 264003, China; 2 Beijing Higher Institution Engineering Research Center of Food Additives and Ingredients, Beijing Key Laboratory of Flavor Chemistry, Beijing Laboratory for Food Quality and Safety, Beijing Technology and Business University, Beijing 100048, China

**Keywords:** *Aspergillus sclerotiorum*, γ-hydroxyl butenolides, cyclopentenones, enantiomer, cytotoxicity, antimicrobial activity

## Abstract

Three new *γ*-hydroxyl butenolides (**1**–**3**), a pair of new enantiomeric spiro-butenolides (**4a** and **4b**), a pair of enantiomeric cyclopentenones (**5a** new and **5b** new natural), and six known compounds (**6**–**11**), were isolated from *Aspergillus sclerotiorum*. Their structures were established by spectroscopic data and electronic circular dichroism (ECD) spectra. Two pairs of enantiomers [(+)/(–)-**6c** and (+)/(–)-**6d**] obtained from the reaction of **6** with acetyl chloride (AcCl) confirmed that **6** was a mixture of two pairs of enantiomers. In addition, the X-ray data confirmed that **7** was also a racemate. The new metabolites (**1**−**5)** were evaluated for their inhibitory activity against cancer and non-cancer cell lines. As a result, compound **1** exhibited moderate cytotoxicity to HL60 and A549 with IC_50_ values of 6.5 and 8.9 µM, respectively, and weak potency to HL-7702 with IC_50_ values of 17.6 µM. Furthermore, compounds **1**−**9** were screened for their antimicrobial activity using the micro-broth dilution method. MIC values of 200 μg/mL were obtained for compounds **2** and **3** towards *Staphylococcus aureus* and *Escherichia coli*, while compound **8** exhibited a MIC of 50 μ/mL towards *Candida albicans*.

## 1. Introduction

*Aspergillus sclerotiorum* widely distributes in various environments such as marine samples, soil, sea-salt field, and rotting apples, and produces a series of bioactive metabolites. Representatives include asterriquinones [1], aspochracin derivative [2], lovastatin analogues [3], butenolides [4], cyclopeptides [5,6,7], sclerotiamide [8], hydroxamic acids [9], and ochratoxin [10]. In addition, co-incubation of this strain with *Penicillium citrinum* resulted in the production of furanone derivatives and alkaloids [11].

**Figure 1:**
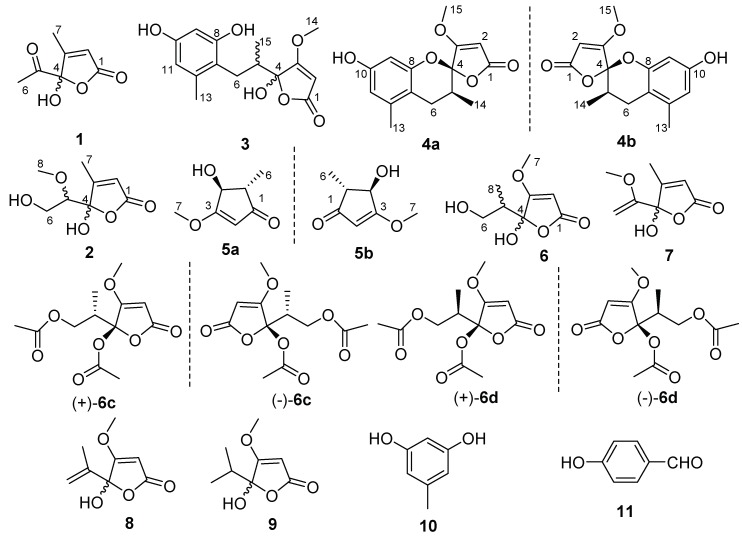
Structures of Compounds **1**−**3**, **4a**, **4b**, **5a**, **5b**, **6**−**11**, (+)/(−)-**6c** and (+)/(−)-**6d**.

The Yellow River Delta is formed mainly by the deposition of sand and mud carried by the Yellow River, which is the world’s youngest wetland ecosystem [12]. High evaporation and tidal intrusion heavily salinizes and alkalizes the soil, half of which is barren and not suitable for the growth of crops [13,14]. The saline soil is typically characterized by poor nutrient and high salinity, which endows the microorganism special biosynthetic pathways during the evolutionary process to produce structurally novel and biologically active secondary metabolites. Up to now, only a few research papers have been carried out on the fungi from this unique environment [15]. In our continuation of investigation on the saline soil-derived fungi from the Yellow River Delta, dozens of natural products (NPs) with multifarious structural features and a wide range of biological activities were obtained [16,17]. As part of our ongoing efforts in seeking for new bioactive NPs, *A. sclerotiorum* JH42 with antimicrobial activity was isolated from saline soil, and subjected to chemical exploration, which led to the achievement of six new (**1**–**3**, **4a**, **4b,** and **5a**), a new natural (**5b**), and six known (**6**–**11**) compounds (Figure 1). Additionally, the isolated *γ*-hydroxyl butenolides in the current work were all proved to undergo tautomerism at C-4 based on the analyses of physicochemical properties, calculated ECD data, and X-ray diffraction. Herein, details of the isolation, structure elucidation, acetylation, cytotoxic, and antimicrobial activities of these compounds are reported.

## 2. Results

**Figure 2:**
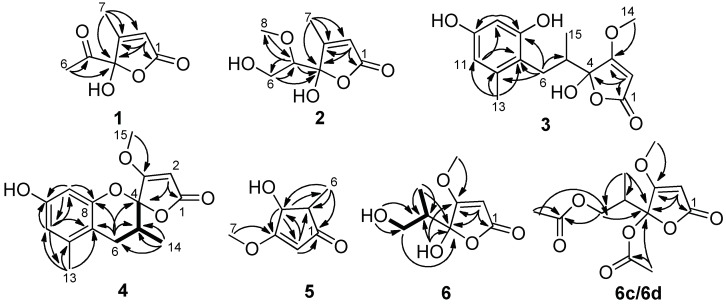
Key COSY and HMBC correlations of **1**–**6**, **6c** and **6d.**

Compound **1** was obtained as an optically inactive colorless oil with the molecular formula of C_7_H_8_O_4_ as determined by the deprotonated-ion HRESIMS at *m/z* 155.0339 [M − H]^−^ (calcd for C_7_H_7_O_4_, 155.0344). Its IR spectrum indicated the presence of hydroxyl (3374 cm^−1^), keto carbonyl (1755 cm^−1^), *α*,*β*-unsaturated lactone groups (1731, 1652 cm^−1^) [18]. The ^1^H NMR spectrum (Table 1) in DMSO-*d*_6_ showed two singlet methyls at *δ*_H_ 2.25 and 1.96, a singlet olefinic proton at *δ*_H_ 6.18, and a hydroxyl group at *δ*_H_ 8.50. Its ^13^C NMR spectrum revealed seven carbon resonances, corresponding to a keto carbonyl (*δ*_C_ 201.5), four diagnostic carbon signals (*δ*_C_ 170.2, 165.7, 118.9 and 105.7) for a *γ*-hydroxyl butenolide moiety [4], and two methyl groups (*δ*_C_ 24.7 and 12.7) (Table 1). The gross structure of **1** was unambiguously established by the HMBC correlations from H-2 to C-1, C-3 and C-4, from H-6 to C-4 and C-5, and from H-7 to C-1, C-3 and C-4 (Figure 2). It was a mixture of inseparable enantiomers, which mutually transformed through the *γ*-keto-acid form as existence in penicillic acid [19].

Compound **2** was also isolated as an optically inactive colorless oil with the molecular formula of C_8_H_12_O_5_ as elucidated by the HRESIMS *m/z* 187.0610 [M − H]^−^ (calcd for C_8_H_11_O_5_, 187.0601). The IR absorption bands at 3373, 1739 and 1652 cm^−1^ suggested the presence of hydroxyl, *α*,*β*-unsaturated lactone groups. The ^1^H NMR spectrum in MeOH-*d*_4_ displayed signals for a singlet olefinic proton at *δ*_H_ 5.86, an oxymethine at *δ*_H_ 3.56 (dd, *J* = 7.0, 3.2 Hz), a diastereotopic methylene at *δ*_H_ 3.90 (m) and 3.66 (dd, *J* = 11.7, 7.0 Hz), a methoxyl at *δ*_H_ 3.50 (s) and a methyl at *δ*_H_ 2.09 (Table 1). The ^13^C NMR spectrum (Table 1), with the help of DEPT and HSQC data, showed the presence of two methyls (one oxygenated), an oxymethylene, an olefinic and an oxymethine, a hemiketal carbon (*δ*_C_ 110.1), and two quaternary carbons (*δ*_C_ 172.9 and 168.7). The above data indicated **2** was also a *γ*-hydroxyl butenolide as **1**. The main differences were the appearances of an oxygenated methyl, an oxymethylene and an oxymethine, together with the disappearances of a carbonyl and a methyl. The planar structure and the assignments of the NMR data were completed by the HMBC correlations (Figure 2). Since no optical rotation and no CD absorption exhibited, compound **2** was also an inseparable racemic mixture.

Compound **3** was a colorless solid with zero optical rotation. Its HRESIMS at *m/z* 293.1021 [M − H]^−^ (calcd for C_15_H_17_O_6_, 293.1020) gave the molecular formula of C_15_H_18_O_6_. The ^1^H NMR spectrum displayed signals for two meta-coupled aromatic protons at *δ*_H_ 6.38 and 6.20 (d, *J* = 1.6 Hz, each), one isolated olefinic proton at *δ*_H_ 5.20, a set of nonequivalent methylene protons at *δ*_H_ 2.58 and 2.08, one methine at *δ*_H_ 2.35 (m), one methoxyl at *δ*_H_ 3.94 and two methyl protons at *δ*_H_ 2.19 and 0.86 (Table 2). The ^13^C NMR spectrum displayed fifteen resonance signals (Table 2). The carbon signals at *δ*_C_ 180.9, 170.7, 106.3, 90.2 and 60.0 showed the representative resonances for a *γ*-hydroxyl-*β*-methoxyl butenolide moiety as in dihydropenicillic acid [20], which was also isolated in the current report. Obviously, two aromatic methines and four aromatic quaternary carbons constructed a tetrasubstituted phenyl ring, and the linkages of substituents were completed by the HMBC correlations (Figure 2). The splitting behaviors of methylene at *δ*_H_ 2.58 (dd, *J* = 13.6, 11.2 Hz) and 2.08 (disturbed by the signals of solvent), methine at *δ*_H_ 2.35 (m) and methyl at *δ*_H_ 0.86 (br s) completed the structure of **3**. The weak carbon signals of C-4, C-6, C-7, and C-14, together with the broad singlet of H-14 (should be doublet) (Figure S15 and S16), implied that the C-4 anomers mutually transformed in solution. Owing to no optical rotation and no CD absorption displayed, **3** existed as an inseparable racemic mixture.

**Figure 3:**
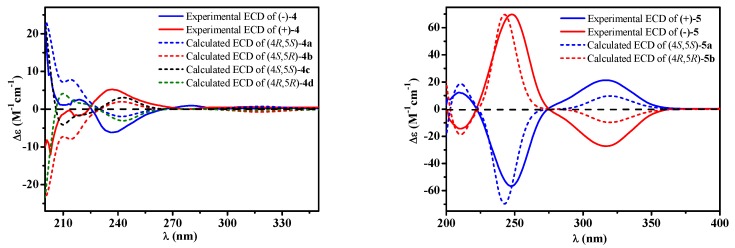
Experimental [(+)/(–)-**4**, (+)/(–)-**5**] and calculated [**4a**, **4b**, **4c**, **4d**, **5a** and **5b**] electronic circular dichroism (ECD) spectra.

Compound **4** was afforded as an optically inactive colorless solid. The HRESIMS *m/z* 277.1071 [M + H]^+^ gave a molecular formula of C_15_H_16_O_5_, which was one H_2_O unit less than that of **3**. Its ^1^H and ^13^C NMR data were similar to those of **3**, except for the chemical shifts of C-4, C/H-5, C/H-6, C-7 and C-8 (Table 2). The above information, especially the chemical shift of C-8 (*δ*_C_ 153.2), implied that 8-OH and 4-OH in **3** should be dehydrated into an ether linkage in **4**. The 2D NMR spectra (Figure 2) established its planar structure, which was different from aspergispiroketal in the locations of the substituents at benzyl moiety [21], as proved by the HMBC correlations from H-6 to C-7 and C-8, from H-9 to C-8, C-10 and C-11, from H-11 to C-7, C-9, C-10 and C-13, and from H-13 to C-7, C-11 and C-12. In view of no optical activity and ECD absorption, **4** was a racemate and separated into (+)-**4** and (–)-**4** using high performance liquid chromatography (HPLC) on a chiral column. Their absolute configurations were determined according to the experimental and calculated ECD data (Figure 3). Based on the optimized structures, the ECD calculation was conducted using time-dependent density functional theory (TD-DFT) at BP86/6-311G (d,p) for four isomers of **4** (Attachment S1). Then the absolute configurations of (+)-**4** and (–)-**4** were determined to be (4*S*,5*R*)-**4b** and (4*R*,5*S*)-**4a**, respectively.

Compound **5**, a colorless solid with zero optical rotation, was assigned the molecular formula of C_7_H_10_O_3_ on the basis of its positive HRESIMS at *m/z* 143.0704 [M + H]^+^ (calcd for C_7_H_11_O_3_, 143.0703). The ^1^H NMR spectrum showed an olefinic proton at *δ*_H_ 6.37 (d, *J* = 2.6 Hz), a oxymethine at *δ*_H_ 4.37 (dd *J* = 2.6, 1.6 Hz), a methine at *δ*_H_ 2.20 (qd, *J* = 7.5, 1.6 Hz), a methoxyl at *δ*_H_ 3.75 (s) and a doublet methyl at *δ*_H_ 1.20 (*J* = 7.5 Hz) (Table 1). The ^13^C NMR spectrum displayed seven carbon signals including a keto carbonyl, two olefinic and four aliphatic carbons (Table 1). The planar structure of **5** was confirmed by the HMBC correlations (Figure 2). In the NOE difference experiment, the signal of H-2 was enhanced when H-6 was irradiated, so H-2 and H-3 were in the opposite directions, which was further confirmed by the small coupling constant (*J* = 1.6 Hz). Compound **5** was subjected to chiral HPLC and separated into (+)-**5** and (−)-**5**. According to the calculated and experimental ECD data (Figure 3), their absolute configurations were determined to be 2*S*,3*S* and 2*R*,3*R* for **5a** and **5b**, respectively. **5b** had been reported as a synthetic intermediate without ^13^C NMR, ECD and specific rotation data reported [22]. Therefore, **5a** was a new compound, while **5b** was a new natural product.

**Figure 4:**
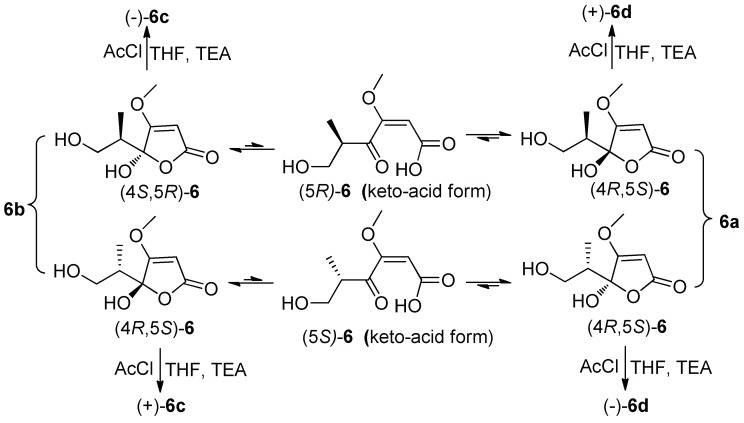
Tautomeric forms (*γ*-keto-acids and *γ*-hydroxyl butenolides) of **6**, and the reaction of **6** with AcCl.

Compound **6** was obtained as colorless blocks with the molecular formula of C_8_H_12_O_5_ on the basis of its negative HRESIMS. Some articles reported its structure with a set of ^1^H and ^13^C NMR data [4,23], but the compound obtained in our project showed two sets of ^1^H and ^13^C NMR data (**6a** and **6b**) (Table 3) with the ratio of about 1:1 in DMSO-*d*_6_, about 2:1 in acetone-*d*_6_ (only ^1^H NMR spectrum measured, Figure S42), about 1.4:1 in MeOH-*d*_4_. Additionally, only H-6, H-8, and C-5 exhibited two sets of NMR signals, and signals of C-3 and C-4 were too weak to be observed in MeOH-*d*_4_ (Figure S43 and S44). The above phenomena suggested **6** was a mixture of two pairs of racemates, and the proportion of anomers of C-4 changed with solvents (Figure 4). The structures and the assignments of the ^1^H and ^13^C NMR data of **6a** and **6b** in DMSO-*d*_6_ were completed by 2D NMR spectra (Figure 2).

**Figure 5:**
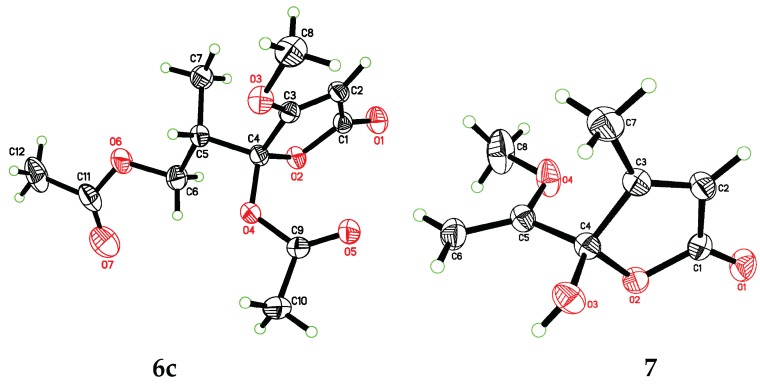
X-ray ORTEP diagrams of **6c** and **7**.

**Figure 6:**
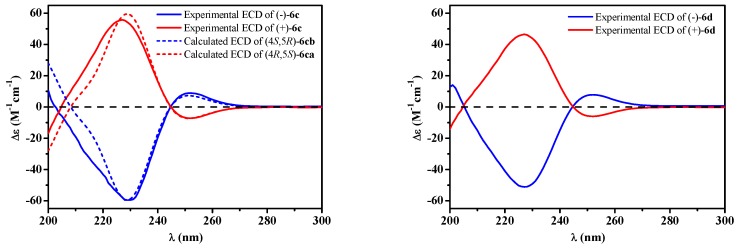
Experimental [(+)/(–)-**6c**, (+)/(–)-**6d**] and calculated [(4*S*,5*R*)/(4*R*,5*S*)-**6c**] ECD spectra.

In order to explore the case, compound **6** was reacted with AcCl leading to the production of **6c** and **6d** with optical inactivity (Figure 4). Their molecular formulae of C_12_H_16_O_7_ were obtained on the basis of their HRESIMS. And their planar structures were constructed by the ^1^H and ^13^C NMR (Table 4) and HMBC spectra (Figure 4). The single crystal X-ray diffraction using Cu Kα radiation showed **6c** to be a centrosymmetric space group P2_1_/C with 4*S*,5*R* and 4*R*,5*S* configurations (Figure 5), so (±)-**6d** should be the 4*S*,5*S* and 4*R*,5*R* configurations. Then they were subjected to chiral HPLC and isolated into (+)/(−)-**6c** and (+)/(−)-**6d**, respectively. The calculated and experimental ECD data (Figure 6) proved the absolute configurations of (+)-**6c** and (−)-**6c** to be the respective 4*R*,5*S* and 4*S*,5*R*. Considering their ECD absorptions mainly resulted from C-4 chiral center, the same ECD data of (+)-**6c** and (+)-**6d**, and (−)-**6c** and (−)-**6d** to the mirror images, implied that (+)-**6d** and (−)-**6d** had 4*R*,5*R* and 4*S*,5*S* configurations, respectively. The *δ* values of C-1, C-2 and C-4 in **6c** were slightly larger than those in **6d** with C-3 and C-5 to the contrary (Table 4), and the same behaviors were also observed in **6b** and **6a** (Table 3), so (±)-**6c** and (±)-**6d** should derive from **6b** and **6a**, separately. Consequently, **6b** should be a racemate with the configurations of 4*S*,5*R* and 4*R*,5*S*, **6a** be 4*S*,5*S* and 4*R*,5*R*.

Compound **7** was isolated as colorless blocks. Its NMR data was almost identical with spersclerotioron G, which was reported as an *S* configuration compound at C-4 chiral center [4]. However, in our report it was optically inactive and of no Cotton effects in its ECD spectrum, therefore it was a racemic mixture, which was confirmed by a centrosymmetric space group P2_1_/n in the single crystal X-ray diffraction with Cu Kα radiation (Figure 5). Other known compounds were identified as penicillic acid (**8**) [20], dihydropenicillic acid (**9**) [20], orcinol (**10**) [24], and *p*-hydroxyl benzaldehyde (**11**) [25], by comparison of their spectroscopic data with those in the literature.

Compounds **1**–**5** were preliminarily evaluated for their cytotoxicity against human promyelocytic leukemia (HL60), human lung adenocarcinoma (A549) and human normal liver (HL-7702) cell lines by the MTT method, with doxorubicin as a positive control (IC_50_: 0.85, 1.5 and 8.3 µM, respectively). Compound **1** and **3** showed selective cytotoxicity against HL60 (IC_50_: 6.5 and 12.1 µM, respectively), A549 (IC_50_: 8.9 and 16.7 µM, respectively), and HL-7702 (IC_50_: 17.6 and 22.8 µM, respectively) cell lines. The results suggested that compounds **1** and **3** showed stronger cytotoxicity to cancer cells than to non-cancer cell lines. The other compounds were inactive to the tested cell lines (IC_50_ > 20 µM).

Meanwhile, the antimicrobial assays of compounds **1**–**9** were screened against *Staphylococcus aureus* (ATCC 25923), *Escherichia coli* (ATCC 25922) and *Candida albicans* (ATCC 10231). Among them, the known compound **8** showed pronounced antimicrobial activity to the tested organisms, while compounds **2** and **3** displayed weak activity against *S. aureus* and *E. coli* (Table 5).

## 3. Discussion

*A. sclerotiorum* JH42 not only produced six new compounds (**1**–**3, 4a**, **4b** and **5a**) and a new natural product (**5b**), but also penicillic acid (**8**) with a high yield (59 g in 73 g of the crude extract). Since penicillic acid possessed multiple bioactivities, such as antitumor [19,26,27,28], antibacterial [4,29], antimalarial [4], phytotoxic [30], antiviral [31], antifungal properties [32], *A. sclerotiorum* JH42 might be employed as a potential producer to provide penicillic acid in industry for the future development and applications.

Additionally, the *γ*-hydroxyl butenolides (**1**–**3**, **6**–**7**) in this study were all proved to be mixtures of enantiomers. The information implied that *γ*-hydroxyl butenolides usually exist in a mixture of anomers of C-4, which were inseparable because of their mutual transformation through the *γ*-keto-acid form. The results have guiding significance for the isolation and structure determination of this kind of compounds.

## 4. Materials and Methods 

### 4.1. General Experimental Procedures

The optical rotations, ultraviolet (UV), IR and ECD spectra were measured on an Autopol V Plus polarimeter (Rudolph Research Analytical, Hackettstown, NJ, USA), a TU-1091 spectrophotometer (Beijing Purkinje General Instrument Co., Beijing, China), Nicolet 6700 spectrophotometer (Thermo Scientific, Waltham, MA, USA) with an attenuated total reflectance (ATR) method, and a Chirascan spectropolarimeter (Applied Photophysics, Leatherhead, United Kingdom), respectively. An Avance 400 (Bruker, Billerica, MS, USA) was used to collect the NMR data. X-ray crystal data were performed on a Bruker Smart 1000 CCD X-ray diffractometer (Bruker Biospin Group, Karlstuhe, Germany). HRESIMS data were acquired on a Q-TOF Ultima GLOBAL GAA076 LC or a 1200RRLC-6520 Accurate-Mass Q-TOF LC/MS mass spectrometer (Agilent, Santa Clara, CA, USA). LC-6AD Liquid Chromatography (Shimadzu, Kyoto, Japan) equipped with an ODS column (HyperClone, 5 μm ODS C_18_ 120 Å, 250 × 10 mm, Phenomenex, 4 mL/min), and a chiral column [ChiralPAK IC, 5 μm cellulose tri(3,5-dichlorophenyl carbamate), 250 × 10 mm, Daicel Chiral Technologies Co. LTD. (Shaihai, China)] was used in the HPLC isolation process. The optical density was measured on a Multiskan FC microplate readers (Thermo Fisher Scientific, Shanghai, China). Silica gel (200−300 mesh, Qingdao Marine Chemical Inc., Qingdao, China), reversed-phase C_18_ silica gel (Pharmacia Fine Chemical Co., Ltd., Uppsala, Sweden) and sephadex LH-20 (Ge Healthcare Bio-Sciences AB, Uppsala, Sweden), were used in column chromatography.

### 4.2. Fungal Material

*A. sclerotiorum* JH42 (Genbank accession No. HQ717801) was isolated from the saline soil collected along the coast of Bohai bay in Zhanhua in August 2008. The working strain was identified according to ITS sequence analysis and assigned the accession number JH42. It was preserved in China General Microbiological Culture Collection Center (Depositary Number: CGMCC NO. 13562). 

### 4.3. Fermentation and Extraction

*A. sclerotiorum* JH42 was cultured on Petri dishes of potato dextrose agar (PDA) at 28 °C for 7 days. A small spoon of spores was transferred into 500-mL conical flasks containing 180 mL culture medium (decoction of 200 g potato, glucose 20 g, maltose 20 g, mannitol 10 g, yeast extract 3 g, KH_2_PO_4_ 0.5 g, MgSO_4_·7H_2_O 0.3 g, dissolved in 1 L seawater), and cultured at 28 °C for 9 days on a rotary shaker at 170 rpm. The culture broth (34.5 L) was filtered into filtrate and mycelia. The former was extracted with ethyl acetate, while the latter was extracted with methanol. The methanol solution was concentrated under reduced pressure to yield an aqueous solution, which was then extracted with ethyl acetate. The ethyl acetate extracts were merged and evaporated under reduced pressure to give an extract (73 g).

### 4.4. Purification

The extract was dissolved in acetone and left for crystallization at room temperature by slow evaporation of the solvent to obtain **8** (18.0 g). Then the residue was performed on a silica gel column chromatography with a step gradient of petroleum ether/ethyl acetate (from 1:0 to 0:1, *v/v*) to afford ten fractions (Fr.s 1–10). Fr. 7 (3.6 g) was separated into seven subfractions (Fr.s 7.1–7.7) by an ODS column eluting with MeOH/H_2_O gradient (from 20:80 to 100:0, *v/v*). Fr. 7.2 (0.2 g) was purified by semipreparative HPLC on an ODS column eluting with 15% MeOH to yield **1** (25.2 mg, *t*_R_ 7.9 min) and **2** (14.2 mg, *t*_R_ 5.6 min). Fr. 4 (1.1 g) was chromatographed on a Sephadex LH-20 column (MeOH) and then purified by HPLC (60% MeOH) to yield **3** (38.0 mg, *t*_R_ 22.1 min). Fr. 7.6 (0.1 g) was purified by HPLC (40% MeOH) on an ODS column to yield **4** (12.0 mg, *t*_R_ 26.8 min), which was further separated by HPLC on a chiral column (n-hexane/isopropanol, 60:40, *v/v*, 2.0 mL/min) to give **4a** (1.8 mg, *t*_R_ 17.3 min) and **4b** (1.7 mg, *t*_R_ 41.5 min). Fr. 7.3 (0.1 g) was separated by HPLC (15% MeOH) to afford **5** (14.6 mg, *t*_R_ 12.2 min), which was further separated by HPLC on a chiral column (n-hexane/isopropanol, 60:40, *v/v*, 2.0 mL/min) to give **5a** (2.5 mg, *t*_R_ 28.6 min) and **5b** (3.3 mg, *t*_R_ 21.3 min). Fr. 6 (1.3 g) was chromatographed on a silica gel column using chloroform/methanol (30:1, *v/v*) to obtain **6** (0.63 g). Fr. 5 (44.0 g) was crystalized in acetone to give **8** (41.0 g) again, and then the residue was isolated by HPLC (25% MeOH) to yield **7** (16.0 mg, *t*_R_ 13.8 min) and **9** (27.0 mg, *t*_R_ 20.3 min). Fr. 8 (0.35 g) was purified by HPLC (15% MeOH) to yield **10** (13.8 mg, *t*_R_ 27.2 min) and **11** (9.8 mg, *t*_R_ 21.2 min).

Aspersclerolide A (**1**): colorless oil (MeOH); UV (MeOH) *λ*_max_ (log *ε*): 210 (3.94) nm; IR (ATR) *ν*_max_ 3374, 1755, 1731, 1652, 1435, 1382, 1360, 1302, 1202, 1149, 1018, 912, 857, 765 cm^−1^; ^1^H and ^13^C NMR data: see Table 1; HRESIMS *m/z* 155.0339 [M − H]^−^ (calcd for C_7_H_7_O_4_, 155.0344).

Aspersclerolide B (**2**): colorless oil (MeOH); UV (MeOH) *λ*_max_ (log *ε*): 206 (3.88) nm; IR (ATR) *ν*_max_ 3373, 2943, 1739, 1652, 1438, 1380, 1185, 1114, 1037, 922, 853, 697 cm^−1^; ^1^H and ^13^C NMR data: see Table 1; HRESIMS *m/z* 187.0610 [M − H]^−^ (calcd for C_8_H_11_O_5_, 187.0601).

Aspersclerolide C (**3**): colorless solid (MeOH); UV (MeOH) *λ*_max_ (log *ε*): 282 (3.35), 221 (4.14), 205 (4.46) nm; IR (ATR) *ν*_max_ 3369, 3251, 1742, 1706, 1630, 1593, 1511, 1456, 1342, 1293, 1267, 1222, 1142, 1095, 985, 911, 806, 780 cm^−1^; ^1^H and ^13^C NMR data: see Table 2; HRESIMS *m/z* 293.1021 [M − H]^−^ (calcd for C_15_H_17_O_6_, 293.1020).

(±)-Aspersclerolide D (**4**): colorless solid (MeOH); UV (MeOH) *λ*_max_ (log *ε*): 279 (3.51), 221 (4.13), 210 (4.11) nm; IR (ATR) *ν*_max_ 3326, 1737, 1679, 1639, 1626, 1594, 1501, 1451, 1373, 1342, 1266, 1173, 1061, 993, 926, 802, 776 cm^−1^; ^1^H and ^13^C NMR data: see Table 2; HRESIMS *m/z* 277.1071 [M + H]^+^ (calcd for C_15_H_17_O_5_, 277.1071). 

(−)-Aspersclerolide D (**4a**): colorless solid (MeOH); ${\left[\alpha \right]}_{D}^{20}$−39.8 (*c* 0.090, MeOH); ECD (MeOH) *λ*_max_ (Δ*ε*) 280 (+0.95), 237 (−6.16), 219 (+2.54) nm.

(+)-Aspersclerolide D (**4b**): colorless solid (MeOH); ${\left[\alpha \right]}_{D}^{20}$+36.9 (*c* 0.084, MeOH); ECD (MeOH) *λ*_max_ (Δ*ε*) 278 (−0.003), 237 (+5.24), 219 (−1.75) nm.

(±)-4-hydroxy-3-methoxy-5-methyl-2-cyclopentenone (**5**): colorless solid (MeOH); UV (MeOH) *λ*_max_ (log *ε*): 246 (3.75) nm; IR (ATR) *ν*_max_ 3405, 2943, 1712, 1627, 1456, 1316, 1252, 1130, 1074, 1006, 937, 888, 844, 794 cm^−1^; ^1^H and ^13^C NMR data: see Table 1; HRESIMS *m/z* 143.0704 [M − H]^−^ (calcd for C_7_H_11_O_3_, 143.0703).

(+)-(4*S*,5*S*)-4-hydroxy-3-methoxy-5-methyl-2-cyclopentenone (**5a**): colorless solid (MeOH); ${\left[\alpha \right]}_{D}^{20}$ +11.11 (*c* 0.13, MeOH); ECD (MeOH) *λ*_max_ (Δ*ε*) 317 (+21.36), 247 (−56.64), 209 (+12.29) nm.

(−)-(4*R*,5*R*)-4-hydroxy-3-methoxy-5-methyl-2-cyclopentenone (**5b**): colorless solid (MeOH); ${\left[\alpha \right]}_{D}^{20}$−12.65 (*c* 0.17, MeOH); ECD (MeOH) *λ*_max_ (Δ*ε*) 317 (−27.20), 248 (+69.81), 210 (−14.32) nm.

6-hydroxyl dihydropenicillic acid (**6**): colorless blocks (MeOH); UV (MeOH) *λ*_max_ (log *ε*): 223 (3.98) nm; IR (ATR) *ν*_max_ 3309, 3112, 1742, 1709, 1622, 1449, 1414, 1356, 1294, 1268, 1223, 1167, 1094, 1038, 1021, 986, 953, 935, 899, 817, 783, 675 cm^−1^; ^1^H and ^13^C NMR data: see Table 3; HRESIMS *m/z* 187.0615[M − H]^−^ (calcd for C_8_H_11_O_5_, 187.0606).

(±)-**6c**: colorless blocks (MeOH); ^1^H and ^13^C NMR data: see Table 4; HRESIMS *m/z* 295.0809 [M + Na]^+^ (calcd for C_12_H_16_O_7_Na, 295.0788).

(+)-**6c**: colorless solid (MeOH); ${\left[\alpha \right]}_{D}^{20}$+43.90 (*c* 0.04, MeOH); ECD (MeOH) *λ*_max_ (Δ*ε*) 252 (−7.22), 227 (+55.82) nm.

(−)-**6c**: colorless solid (MeOH); ${\left[\alpha \right]}_{D}^{20}$−46.15 (*c* 0.03, MeOH); ECD (MeOH) *λ*_max_ (Δ*ε*) nm; 252 (+8.92), 229 (−59.62) nm.

(±)-**6d**: colorless solid (MeOH); ^1^H and ^13^C NMR data: see Table 4; HRESIMS *m/z* 295.0780 [M + Na]^+^ (calcd for C_12_H_16_O_7_Na, 295.0788).

(+)-**6d**: colorless solid (MeOH); ${\left[\alpha \right]}_{D}^{20}$+78.57 (*c* 0.08, MeOH); ECD (MeOH) *λ*_max_ (Δ*ε*) 252 (−6.01), 227 (+46.51) nm.

(−)-**6d**: colorless solid (MeOH); ${\left[\alpha \right]}_{D}^{20}$ −80.00 (*c* 0.06, MeOH); ECD (MeOH) *λ*_max_ (Δ*ε*) 252 (+7.82), 227 (−51.14) nm.

X-ray Single-Crystal Structure Determinations of **6c** and **7**. Colorless crystals of **6c** and **7** were obtained from MeOH. Their structures were solved by direct methods using the SHELXTL software package and refined by least squares minimization. The crystallographic data for **6c** (deposition number: CCDC 1912596) and **7** (deposition number: CCDC 1912598) have been deposited in the Cambridge Crystallographic Data Centre. These data can be obtained free of charge from the Cambridge Crystallographic Data Centre via www.ccdc.cam.ac.uk/data_request/cif.

Crystal data for **6c**. C_12_H_16_O_7_, *M*_r_ = 272.25, Monoclinic, space group P2_1_/c, unit cell dimensions *a* = 8.6937(3) Å, *b* = 22.0810(5) Å, *c* = 7.9551(2) Å, *V* = 1392.71(7) Å^3^, *Z* = 4, *D*_c__alcd_ = 1.298 g/cm^3^, *F*(000) = 576. *α* = *γ* = 90.00, *β* = 114.218(4); A total of 2427 unique reflections were collected, with 2123 reflections greater than *I* ≥ 2*σ* (*I*) (*R*_int_ = 0.0165). The structure was solved by direct methods and refined by full-matrix least-squares on *F*^2^, with anisotropic displacement parameters for non-hydrogen atoms at final *R* indices [*I* > 2*σ* (*I*)], *R*_1_ = 0.0445, *wR*_2_ = 0.1199; *R* indices (all data), *R*_1_ = 0.0503, *wR*_2_ = 0.1249. 

Crystal data for **7**. C_8_H_10_O_4_, *M*_r_ = 170.16, Monoclinic, space group P2_1_/n, unit cell dimensions *a* = 7.6359(3) Å, *b* = 13.2597(4) Å, *c* = 8.5653(3) Å, *V* = 852.49(5) Å^3^, *Z* = 4, *D*_c__alcd_ = 1.326 g/cm^3^, *F*(000) = 360. *α* = *γ* = 90.00, *β* = 100.581(2); A total of 1482 unique reflections were collected, with 1394 reflections greater than *I* ≥ 2*σ* (*I*) (*R*_int_ = 0.0185). The structure was solved by direct methods and refined by full-matrix least-squares on *F*^2^, with anisotropic displacement parameters for non-hydrogen atoms at final *R* indices [*I* > 2*σ* (*I*)], *R*_1_ = 0.0414, *wR*_2_ = 0.1115; *R* indices (all data), *R*_1_ = 0.0436, *wR*_2_ = 0.1130. 

### 4.5. Preparation and Isolation of (±)-6c and (±)-6d

AcCl (41μL) was added to a solution of **6** (53 mg) and triethylamine (TEA) (79 μL) in THF (5 mL). The mixed solution was stirred at room temperature for 2 h. After removal of the solvent under reduced pressure, 5 mL of distilled water was added into the residue and subsequently extracted with ethyl acetate (5 × 3 mL). The ethyl acetate layer was evaporated under reduced pressure to give a crude product, followed by purification on HPLC with an ODS column (40% MeOH) to give **6c** (11.6 mg, *t*_R_ 15.6 min) and **6d** (10.8 mg, *t*_R_ 16.6 min). **6c** and **6d** were further separated by HPLC on a chiral column (n-hexane/isopropanol, 60:40, *v/v*, 2.0 mL/min) to give (+)-**6c** (5.2 mg, *t*_R_ 68.2 min), (−)-**6c** (4.8 mg, *t*_R_ 48.2 min), (+)-**6d** (4.4 mg, *t*_R_ 74.2 min) and (−)-**6d** (4.1 mg, *t*_R_ 47.5 min), respectively. 

### 4.6. Biological Assay

The cytotoxic activity against HL60, A549 and HL-7702 cell lines was performed by the MTT method as previously described [33].

The antimicrobial assays against *S. aureus*, *E. coli* and *C. albicans* were carried out by the broth microdilution method [34]. The tested organisms were incubated overnight with shaking (200 rpm) in thermostatic oscillation incubator (37 °C) in Mueller Hinton broth (MHB) and liquid Sabourand medium for the bacteria and the fungus, respectively. The microbial inoculum density was adjusted to 1 × 10^6^ cfu/mL with 0.9% saline solution by comparison with a MacFarland standard. The tested substances and positive drugs were dissolved in methanol to an initial concentration of 40 mg/mL. 4 μL of initial compound solution and 196 μL of MHB (liquid Sabourand medium for fungus) were added into the first well and mixed evenly. Then 100 μL of solution from the first hole, along with 2 μL of methanol and 98 μL of MHB (liquid Sabourand medium for fungus), were transferred to the second hole, and then shaken up as mixture uniform. The repetitive operation was performed to the eleventh one, from which 100 μL of solution well was discarded. Then, 100 μL of microbial suspension was added to the solutions in 96-well to achieve a final volume of 200 µL and final sample concentrations from 400 to 0.39 µg/mL. The blank well was also incubated with only medium under the same conditions. All experiments were carried out in triplicate and with chloramphenicol and ketoconazole as the positive controls. Optical density measurement for bacteria and fungus was recorded at 620 nm after incubation at 37 °C for 12 and 24 h, respectively. The minimal inhibitory concentration (MIC) was defined as the concentration at which the growth was inhibited 80% of the tested microorganisms [35].

## 5. Conclusions

In summary, three new (**1**–**3**) and four known (**6**–**9**) *γ*-hydroxyl butenolides, a pair of new enantiomeric spiro-butenolides (**4a** and **4b**), a pair of enantiomeric cyclopentenones (**5a** new and **5b** new natural), along with orcinol (**10**) and *p*-hydroxyl benzaldehyde (**11**), were isolated from *A. sclerotiorum* JH42. The acquisition of two pairs of enantiomers [(+)/(−)-**6c**) and (+)/(−)-**6d**] by the reaction of **6** with AcCl confirmed that **6** was a mixture of two pairs of enantiomers. In addition, the X-ray diffraction data of **7** revealed it was also a racemic mixture. Compound **1** exhibited moderate cytotoxicity against HL60 and A549 cell lines with IC_50_ values of 6.5 and 8.9 µM, respectively. New compounds **2** and **3** showed weak antibacterial activity against *S. aureus* and *E. coli*, while **8** displayed pronounced antimicrobial activity against all the tested organisms.

## Figures and Tables

**Table 1:** ^1^H (400 MHz) and ^13^C (100 MHz) NMR data for **1**, **2** and **5** [*δ*_H_, mult (*J* in Hz)].

Position	1 ^a^		2 ^b^		5 ^b^	
	δ_C_	δ_H_	δ_C_	δ_H_	δ_C_	δ_H_
1	170.2, C		172.9, C		203.8, C	
2	118.9, C	6.18, s	119.5, CH	5.86, br s	127.6, CH	6.37, d (2.6)
3	165.7, C		168.7, C		158.2, C	
4	105.7, C		110.1, C		74.2, CH	4.37, dd (2.6, 1.6)
5	201.5, C		84.2, CH	3.56, dd (7.0, 3.2)	51.1, CH	2.20, dq (7.5, 1.6)
6	24.7, CH	2.25, s	62.4, CH_2_	3.90, m; 3.66, dd (11.7, 7.0)	13.3, CH_3_	1.20, d (7.5)
7	12.7, CH	1.96, s	13.4, CH_3_	2.09, br s	57.7, CH_3_	3.75, s
8			60.6, CH_3_	3.50, s		
OH		8.50, s				

^a^ NMR spectra obtained in DMSO-*d*_6_; ^b^ NMR spectra obtained in MeOH-*d*_4_.

**Table 2:** ^1^H (400 MHz) and ^13^C (100 MHz) NMR data for **3** and **4** in acetone-*d*_6_.

Position	3		4	
	δ_C_	δ_H_, mult (*J* in Hz)	δ_C_	δ_H_, mult (*J* in Hz)
1	170.7, C		169.9, C	
2	90.2, CH	5.20, s	91.2, CH	5.44, s
3	180.9, C		178.4, C	
4	106.3, C		104.2, C	
5	39.7, CH	2.35, m	31.4, CH	2.35 ^c^
6	26.4, CH_2_	2.58, dd (13.6, 11.2); 2.08 ^a^	27.1, CH_2_	2.77, m; 2.35 ^c^
7	117.1, C		112.2, C	
8	157.3, C		153.2, C	
9	101.2, CH	6.38, d (1.6)	101.8, CH	6.18, d (2.2)
10	156.8, C		157.3, C	
11	109.7, CH	6.20, d (1.6)	111.9, CH	6.38, d (2.2)
12	139.4, C		138.6, C	
13	20.1, CH_3_	2.19, s	19.1, CH_3_	2.16, s
14	60.0, CH_3_	3.94, s	60.7, CH_3_	4.07, s
15	13.3, CH_3_	0.86, br s ^b^	15.1, CH_3_	0.99, d (5.6)

^a^ Disturbed by solvent; ^b^ Caused by the mutually transformed anomers of C-4 in solution; ^c^ Overlapping signals.

**Table 3:** ^1^H (400 MHz) and ^13^C (100 MHz) NMR data for **6a** and **6b** in DMSO-*d*_6_.

Position	6a		6b	
	δ_C_	δ_H_, mult (*J* in Hz)	δ_C_	δ_H_, mult (*J* in Hz)
1	169.9, C		170.0, C	
2	89.3, CH	5.28, s	89.4, CH	5.23, s
3	179.9, C		179.5, C	
4	103.7, C		104.3, C	
5	41.6, CH	2.02, m	40.5, CH	1.99, m
6	61.4, CH_2_	3.39, dt (10.5, 4.6); 3.09, m	61.1, CH_2_	3.76, dt (10.5, 5.0); 3.19, m
7	59.6, CH_3_	3.83, s	59.7, CH_3_	3.86, s
8	11.0, CH_3_	0.93, d (6.9)	11.3, CH_3_	0.78, d (6.9)
4-OH		7.44, s		7.52, s
6-OH		4.51, t (5.3)		4.68, t (5.3)

**Table 4:** ^1^H (400 MHz) and ^13^C (100 MHz) NMR data for (±)-**6c** and (±)-**6d** in DMSO-*d*_6_.

Position	(±)-6c		(±)-6d	
	δ_C_	δ_H_, mult (*J* in Hz)	δ_C_	δ_H_, mult (*J* in Hz)
1	168.6, C		168.5, C	
2	91.2, CH	5.66, s	91.1, CH	5.64, s
3	177.1, C		177.3, C	
4	102.4, C		102.2, C	
5	37.8, CH	2.4, m	38.2, CH	2.45, m
6	62.9, CH_2_	4.25, dd (11.2, 4.8);	63.0, CH_2_	3.93, dd (11.6, 6.2);
		3.96, dd (11.2, 7.0)		3.86, dd (11.6, 6.0)
7	60.5, CH_3_	3.92, s	60.5, CH_3_	3.92, s
8	10.6, CH_3_	0.89, d (7.0)	10.8, CH_3_	1.03, d (6.9)
9	170.2, C		170.1, C	
10	20.6, CH_3_	2.02, s	20.6, CH_3_	2.06, s
11	167.9, C		168.0, C	
12	21.1, CH_3_	2.08, s	21.1, CH_3_	2.08, s

**Table 5:** Antimicrobial Activity of Compounds **1**–**9** (MIC μg/mL).

	1	2	3	4	5	6	7	8	9	Control
*S. aureus*	> 400	200	200	> 400	-	> 400	> 400	6.25	> 400	3.12 ^a^
*E. coli*	> 400	200	200	> 400	-	-	-	12.5	-	6.25 ^a^
*C. albicans*	-	-	-	-	-	-	-	50	-	6.25 ^b^

^a^ chloramphenicol; ^b^ ketoconazole; - no activity.

## Supplementary Materials

The following are available online at https://www.mdpi.com/1420-3049/24/14/2642/s1, Figure S1: ^1^H NMR spectrum (400 MHz) of **1** in DMSO-*d*_6_, Figure S2: ^13^C NMR spectrum (100 MHz) of **1** in DMSO-*d*_6_, Figure S3: HMBC spectrum of **1** in DMSO-*d*_6_, Figure S4: IR spectrum of **1**, Figure S5: UV spectrum of **1** in MeOH, Figure S6: HRESIMS of **1**, Figure S7: ^1^H NMR spectrum (400 MHz) of **2** in MeOH-*d*_4_, Figure S8: ^13^C NMR spectrum (100 MHz) of **2** in MeOH-*d*_4_, Figure S9: DEPT 135 spectrum of **2** in MeOH-*d*_4_, Figure S10: HSQC spectrum of **2** in MeOH-*d*_4_, Figure S11: HMBC spectrum of **2** in MeOH-*d*_4_, Figure S12: IR spectrum of **2**, Figure S13: UV spectrum of **2** in MeOH, Figure S14: HRESIMS of **2**, Figure S15: ^1^H NMR spectrum (400 MHz) of **3** in acetone-*d*_6_, Figure S16: ^13^C NMR spectrum (100 MHz) of **3** in acetone-*d*_6_, Figure S17: HMBC spectrum of **3** in acetone-*d*_6_, Figure S18: UV spectrum of **3** in MeOH, Figure S19: IR spectrum of **3**, Figure S20: HRESIMS of **3**, Figure S21: ^1^H NMR spectrum (400 MHz) of **4** in acetone-*d*_6_, Figure S22: ^13^C NMR spectrum (100 MHz) of **4** in acetone-*d*_6_, Figure S23: DEPT spectrum of **4** in acetone-*d*_6_, Figure S24: HMQC spectrumof **4** in acetone-*d*_6_, Figure S25: ^1^H-^1^H COSY spectrum of **4** in acetone-*d*_6_, Figure S26: HMBC spectrum of **4** in acetone-*d*_6_, Figure S27: IR spectrum of **4**, Figure S28: UV spectrum of **4** in MeOH, Figure S29: HRESIMS of **4**, Figure S30: ^1^H NMR spectrum (400 MHz) of **5** in MeOH-*d*_4_, Figure S31: ^13^C NMR spectrum (100 MHz) of **5** in MeOH-*d*_4_, Figure S32: HMBC spectrum of **5** in MeOH-*d*_4_, Figure S33: NOE difference spectrum of **5** in MeOH-*d*_4_, Figure S34: IR spectrum of **5**, Figure S35: UV spectrum of **5** in MeOH, Figure S36: HRESIMS of **5**, Figure S37: ^1^H NMR spectrum (400 MHz) of **6** in DMSO-*d*_6_, Figure S38: ^13^C NMR spectrum (100 MHz) of **6** in DMSO-*d*_6_, Figure S39: ^1^H-^1^H COSY spectrum of **6** in DMSO-*d*_6_, Figure S40: HSQC spectrum of **6** in DMSO-*d*_6_, Figure S41: HMBC spectrum of **6** in DMSO-*d*_6_, Figure S42: ^1^H NMR spectrum (400 MHz) of **6** in acetone-*d*_6_, Figure S43: ^1^H NMR spectrum (400 MHz) of **6** in MeOH-*d*_4_, Figure S44: ^13^C NMR spectrum (100 MHz) of **6** in MeOH-*d*_4_, Figure S45: HSQC spectrum of **6** in MeOH-*d*_4_, Figure S46: HRESIMS of **6**, Figure S47: ^1^H NMR spectrum (400 MHz) of **6c** in DMSO-*d*_6_, Figure S48: ^13^C NMR spectrum (100 MHz) of **6c** in DMSO-*d*_6_, Figure S49: HMBC spectrum of **6c** in DMSO-*d*_6_, Figure S50: HRESIMS of **6c**, Figure S51: ^1^H NMR spectrum (400 MHz) of **6d** in DMSO-*d*_6_, Figure S52: ^13^C NMR spectrum (100 MHz) of **6d** in DMSO-*d*_6_, Figure S53: HMBC spectrum of **6d** in DMSO-*d*_6_, Figure S54: HRESIMS of **6d**, Attachment **S1**: Supporting information for the calculated ECD spectra of compounds **4**, **5**, and **6c**.
