# Supplementary material for: New Butenolides and Cyclopentenones from Saline Soil-Derived Fungus Aspergillus Sclerotiorum
Source: Molecules. 2019 Jul 21;24(14):2642. doi: 10.3390/molecules24142642 (PMC6680918; doi:10.3390/molecules24142642)
Supplement: Supplementary file 1 [file molecules-24-02642-s001.pdf]

SUPPORTING INFORMATION

New butenolides and cyclopentenones from saline soil-derived fungus *Aspergillus sclerotiorum*

Li-Ying Ma [1], Huai-Bin Zhang [1], Hui-Hui Kang [1], Mei-Jia Zhong [1], De-Sheng Liu [1,*], Hong Ren [2] and Wei-Zhong Liu [1,*]

[1] College of Pharmacy, Binzhou Medical University, Yantai 264003, China; maliyingbz@163.com (L.-Y.M.); zhanghuaibinhua@163.com (H.-B.Z.); kanghuihui_1993@126.com (H.-H.K.); 18660627015@163.com (M.-J.Z.)

[2] Beijing Higher Institution Engineering Research Center of Food Additives and Ingredients, Beijing Key Laboratory of Flavor Chemistry, Beijing Laboratory for Food Quality and Safety, Beijing Technology and Business University, Beijing, 100048, China; renhong@th.btbu.edu.cn (H.R.)

***** Correspondence: lwz1963@163.com (W.-Z.L.); Tel.: +86-535-691-3205 (W.-Z.L.); desheng_liu@sina.com (D.-S.L.)

Table of Contents

Figure S1. ^{1}H NMR spectrum (400 MHz) of **1** in DMSO-d_6. 1
Figure S2. ^{13}C NMR spectrum (100 MHz) of **1** in DMSO-d_6. 1
Figure S3. HMBC spectrum of **1** in DMSO-d_6. 2
Figure S4. IR spectrum of **1**. 2
Figure S5. UV spectrum of **1** in MeOH. 3
Figure S6. HRESIMS of **1**. 3
Figure S7. ^{1}H NMR spectrum (400 MHz) of **2** in MeOH-d_4. 4
Figure S8. ^{13}C NMR spectrum (100 MHz) of **2** in MeOH-d_4. 4
Figure S9. DEPT 135 spectrum of **2** in MeOH-d_4. 5
Figure S10. HSQC spectrum of **2** in MeOH-d_4. 5
Figure S11. HMBC spectrum of **2** in MeOH-d_4. 6
Figure S12. IR spectrum of **2**. 6
Figure S13. UV spectrum of **2** in MeOH. 7
Figure S14. HRESIMS of **2**. 7
Figure S15. ^{1}H NMR spectrum (400 MHz) of **3** in acetone-d_6. 8
Figure S16. ^{13}C NMR spectrum (100 MHz) of **3** in acetone-d_6. 8
Figure S17. HMBC spectrum of **3** in acetone-d_6. 9
Figure S18. IR spectrum of 3. 9
Figure S19. UV spectrum of **3** in MeOH.. 10
Figure S20. HRESIMS of **3**. 10
Figure S21. ^{1}H NMR spectrum (400 MHz) of **4** in acetone-d_6. 11
Figure S22. ^{13}C NMR spectrum (100 MHz) of **4** in acetone-d_6. 11
Figure S23. DEPT spectrum of **4** in acetone-d_6. 12
Figure S24. HSQC spectrumof **4** in acetone-d_6. 12
Figure S25. ^{1}H-^{1}H COSY spectrum of **4** in acetone-d_6. 13
Figure S26. HMBC spectrum of **4** in acetone-d_6. 13
Figure S27. IR spectrum of **4**. 14
Figure S28. UV spectrum of **4** in MeOH. 14
Figure S29. HRESIMS of **4**. 15
Figure S30. ^{1}H NMR spectrum (400 MHz) of **5** in MeOH-d_4. 15
Figure S31. ^{13}C NMR spectrum (100 MHz) of **5** in MeOH-d_4. 16
Figure S32. HMBC spectrum of **5** in MeOH-d_4. 16
Figure S33. NOE difference spectrum of **5** in MeOH-d_4. 17
Figure S34. IR spectrum of **5**. 17
Figure S35. UV spectrum of **5** in MeOH. 18
Figure S36. HRESIMS of **5**. 18
Figure S37. ^{1}H NMR spectrum (400 MHz) of **6** in DMSO-d_6. 19
Figure S38. ^{13}C NMR spectrum (100 MHz) of **6** in DMSO-d_4. 19
Figure S39. ^{1}H-^{1}H COSY spectrum of **6** in DMSO-d_6. 20
Figure S40. HSQC spectrum of **6** in DMSO-d_6. 20
Figure S41. HMBC spectrum of **6** in DMSO-d_6. 21
Figure S42. ^{1}H NMR spectrum (400 MHz) of **6** in acetone-d_6. 21
Figure S43. ^{1}H NMR spectrum (400 MHz) of **6** in MeOH-d_4. 22

Figure S44. ^{13}C NMR spectrum (100 MHz) of **6** in MeOH-d_4. 22
Figure S45. HSQC spectrum of **6** in MeOH-d_4. 23
Figure S46. HRESIMS of **6**. 23
Figure S47. ^{1}H NMR spectrum (400 MHz) of **6c** in DMSO-d_6. 24
Figure S48. ^{13}C NMR spectrum (100 MHz) of **6c** in DMSO-d_6. 24
Figure S49. HMBC spectrum of **6c** in DMSO-d_6. 25
Figure S50. HRESIMS of **6c**. 25
Figure S51. ^{1}H NMR spectrum (400 MHz) of **6d** in DMSO-d_6. 26
Figure S52. ^{13}C NMR spectrum (100 MHz) of **6d** in DMSO-d_6. 26
Figure S53. HMBC spectrum of **6d** in DMSO-d_6. 27
Figure S54. HRESIMS of **6d**. 27
Attachment S1. Supporting information for the calculated ECD spectra of compounds **4**, **5**, and **6c**. 28

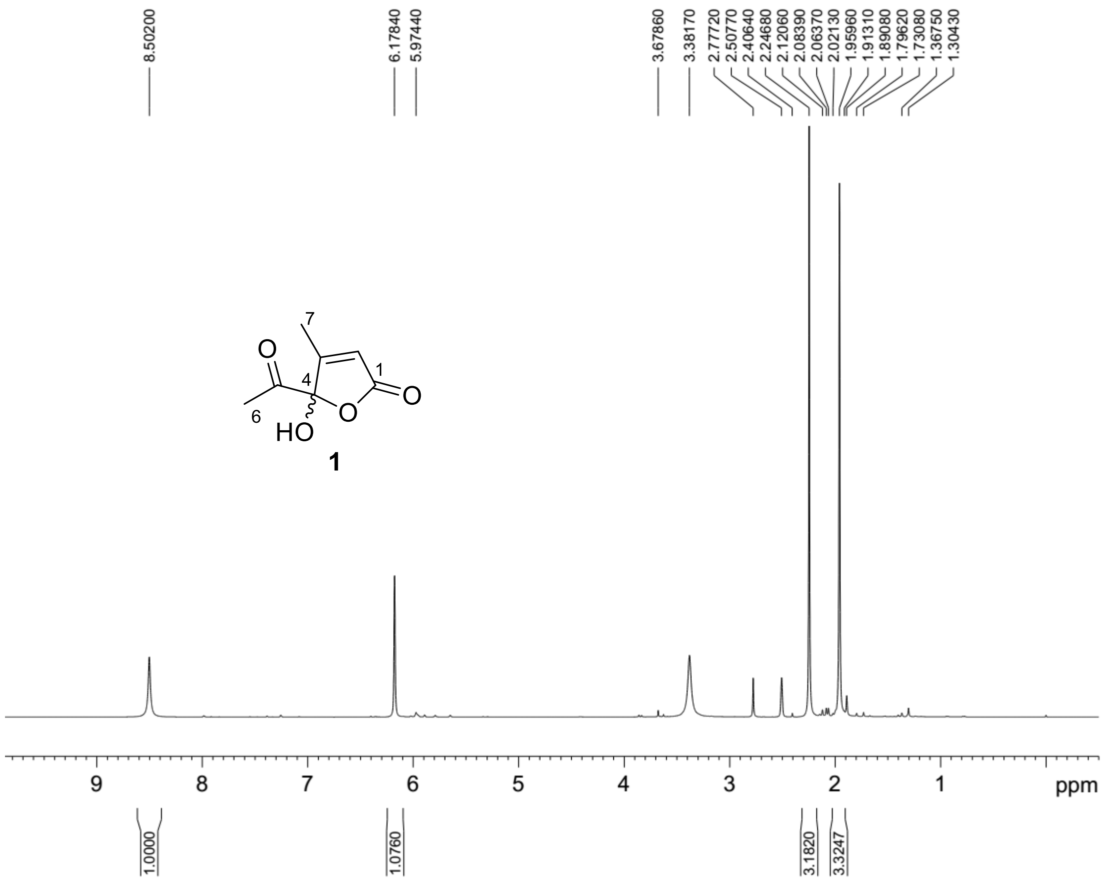

Figure S1. ^{1}H NMR spectrum (400 MHz) of **1** in DMSO-d_6.

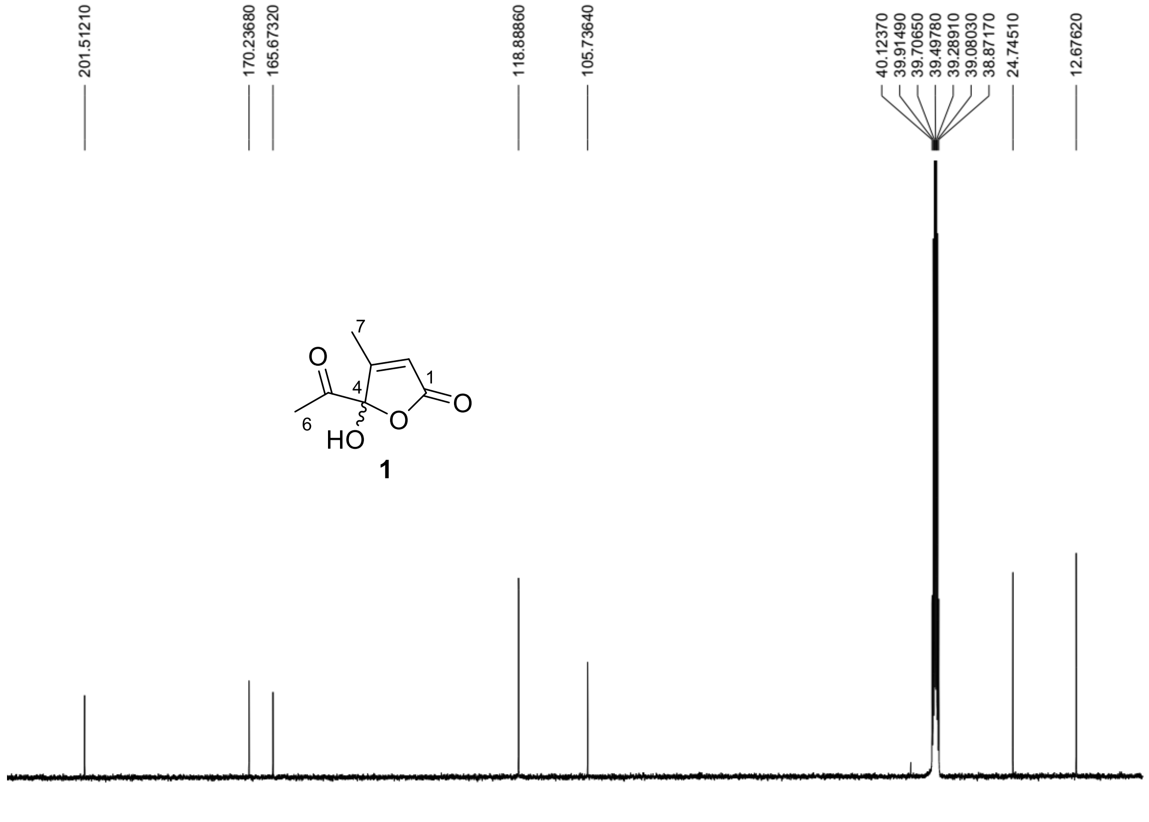

Figure S2. ^{13}C NMR spectrum (100 MHz) of **1** in DMSO-d_6.

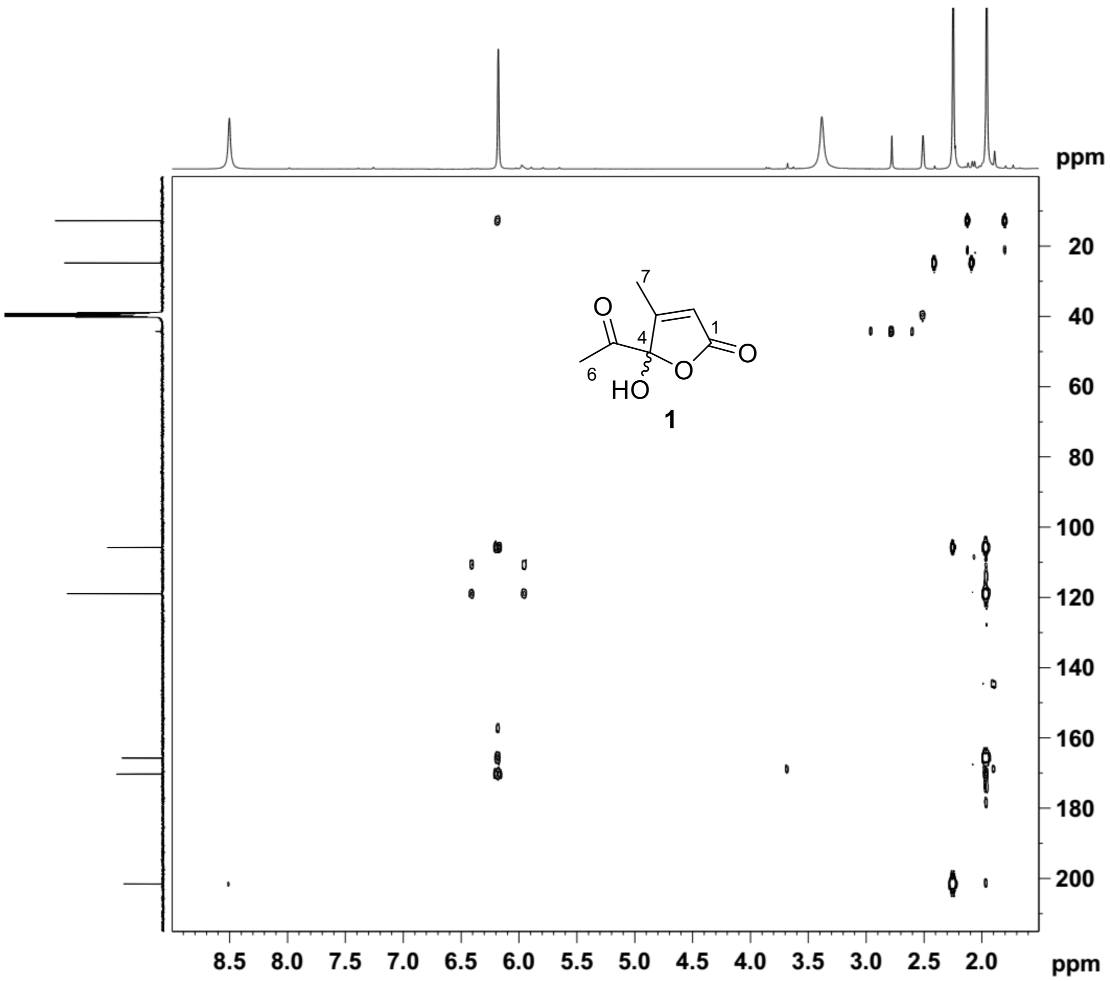

Figure S3. HMBC spectrum of **1** in DMSO-d_6.

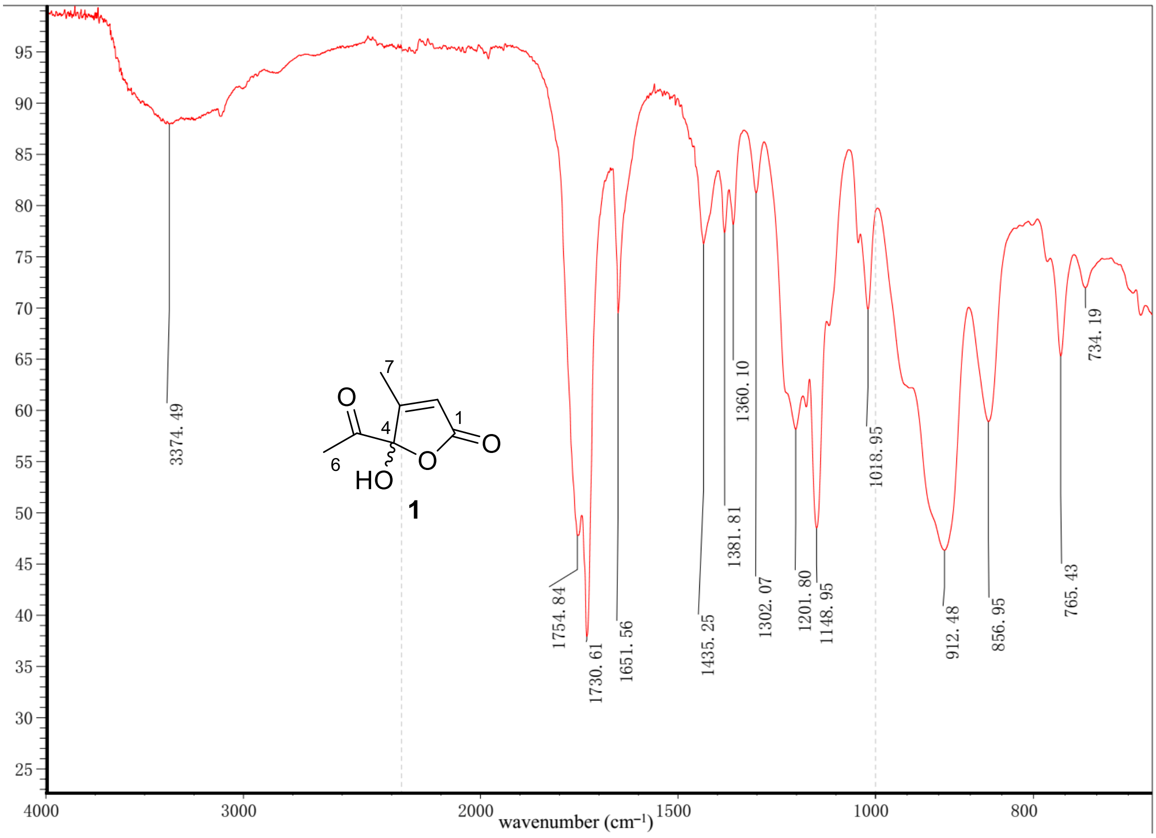

Figure S4. IR spectrum of **1**.

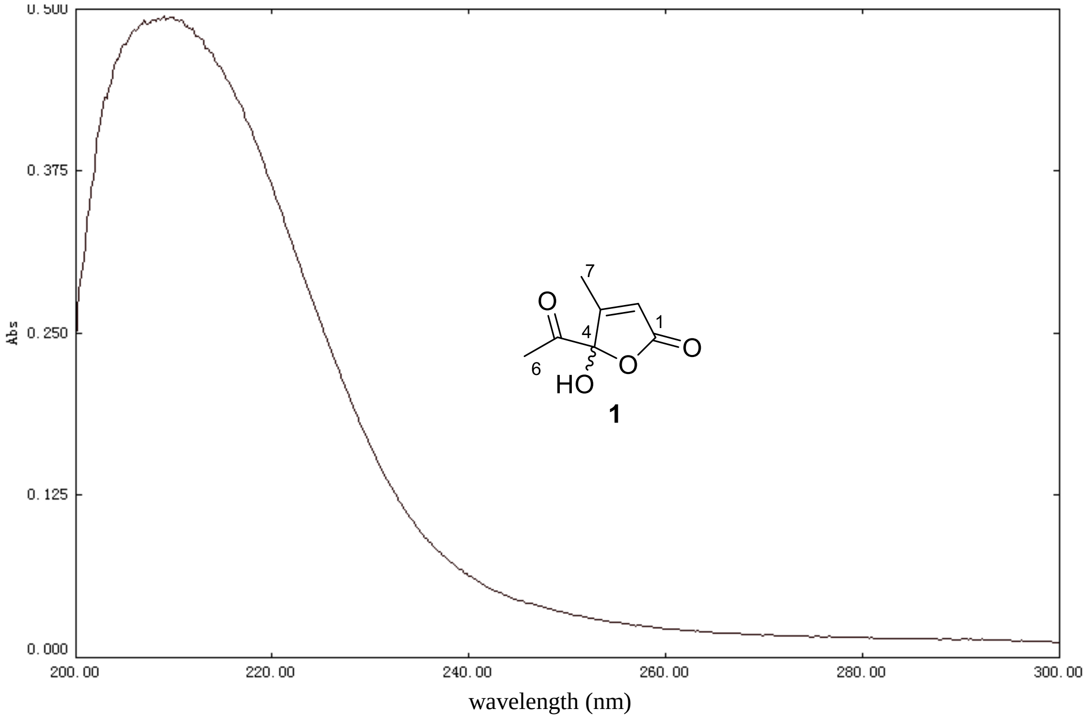

Figure S5. UV spectrum of **1** in MeOH.

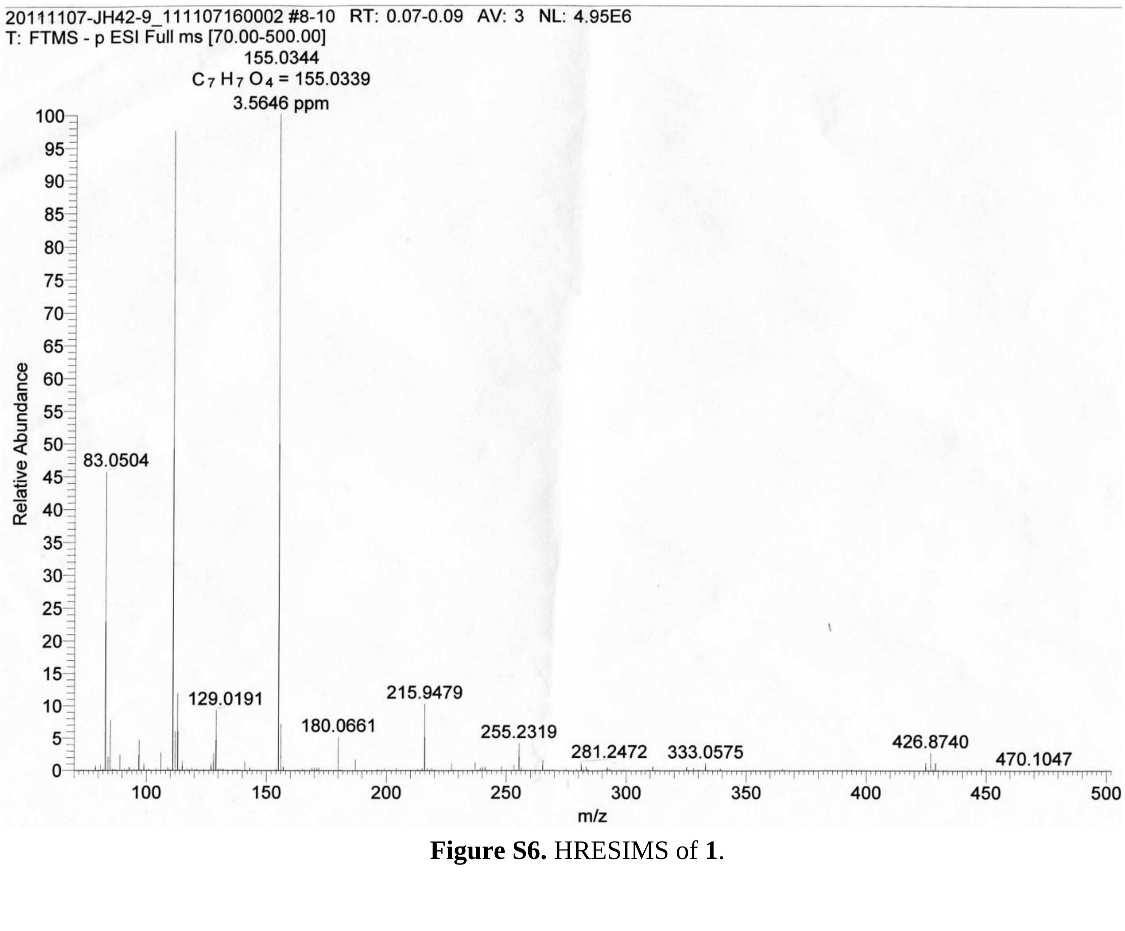

Figure S6. HRESIMS of **1**.

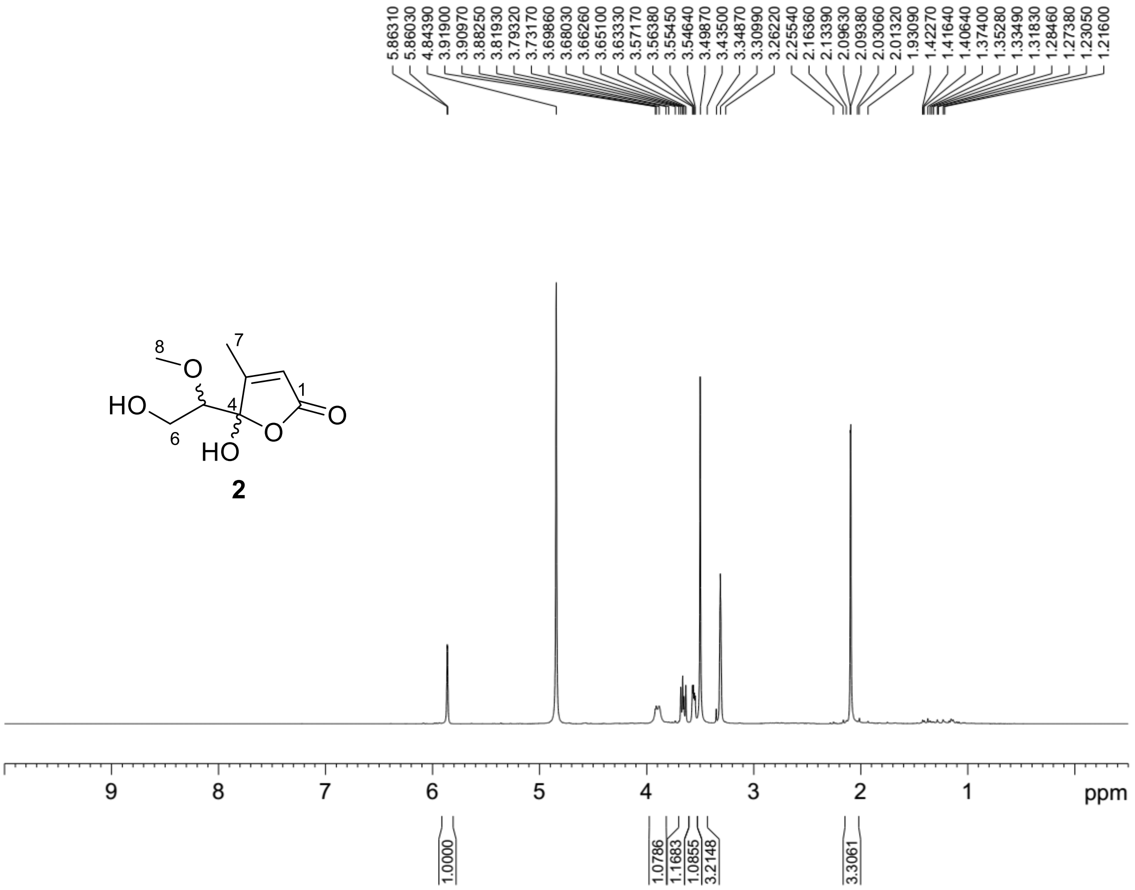

Figure S7. ^{1}H NMR spectrum (400 MHz) of **2** in MeOH-d_4.

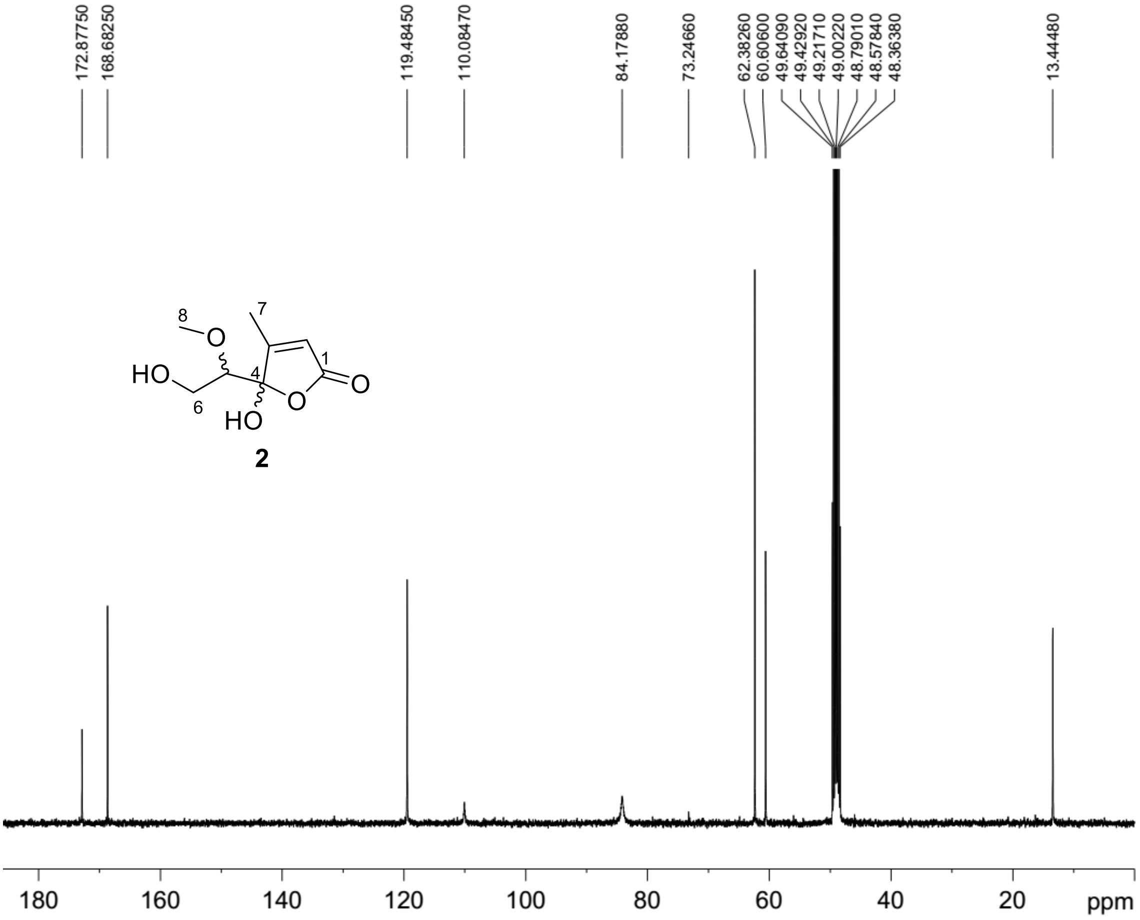

Figure S8. ^{13}C NMR spectrum (100 MHz) of **2** in MeOH-d_4.

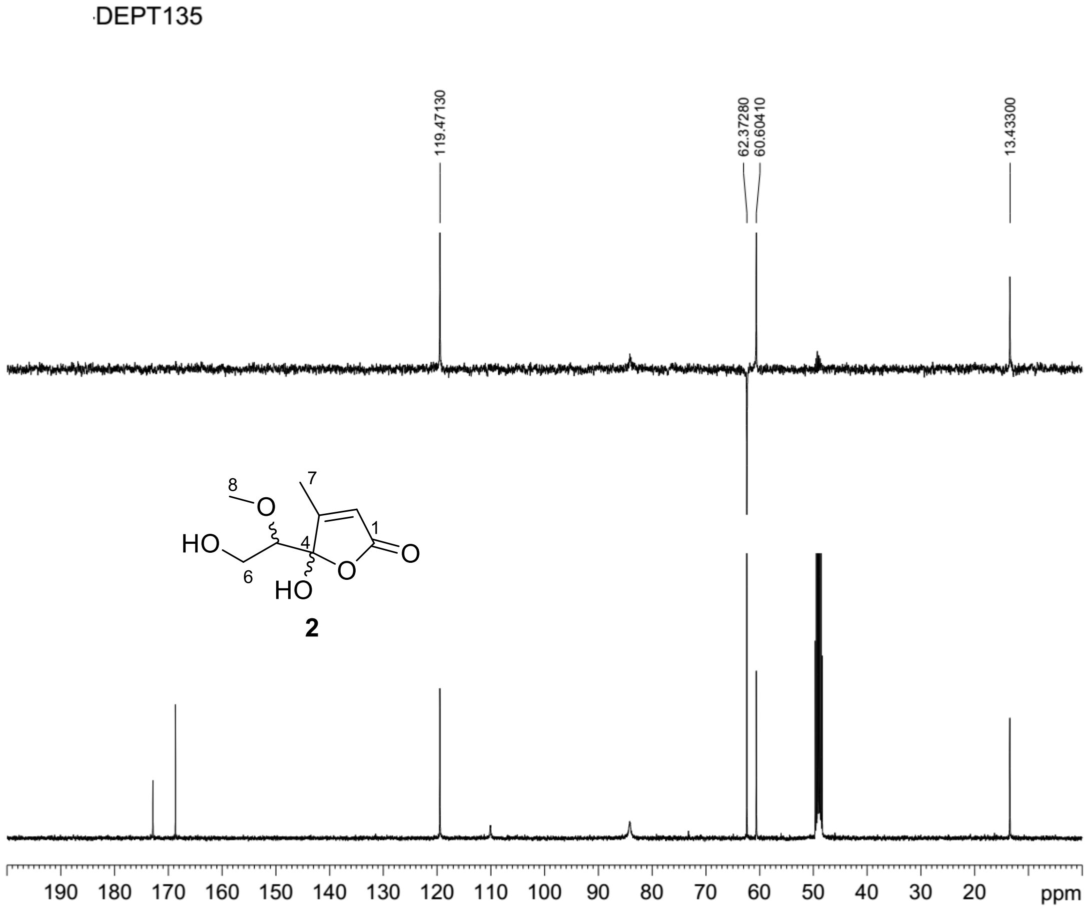

Figure S9. DEPT 135 spectrum of **2** in MeOH-d_4**.**

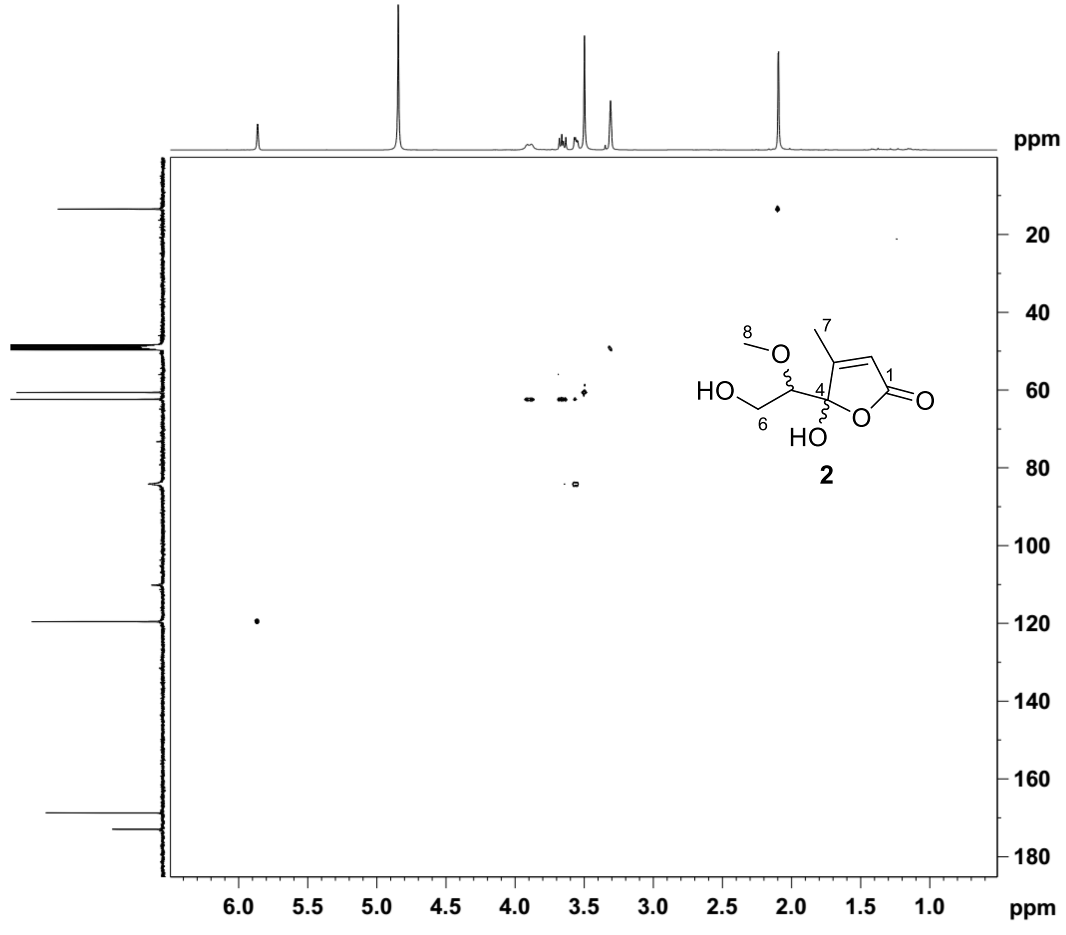

Figure S10. HSQC spectrum of **2** in MeOH-d_4.

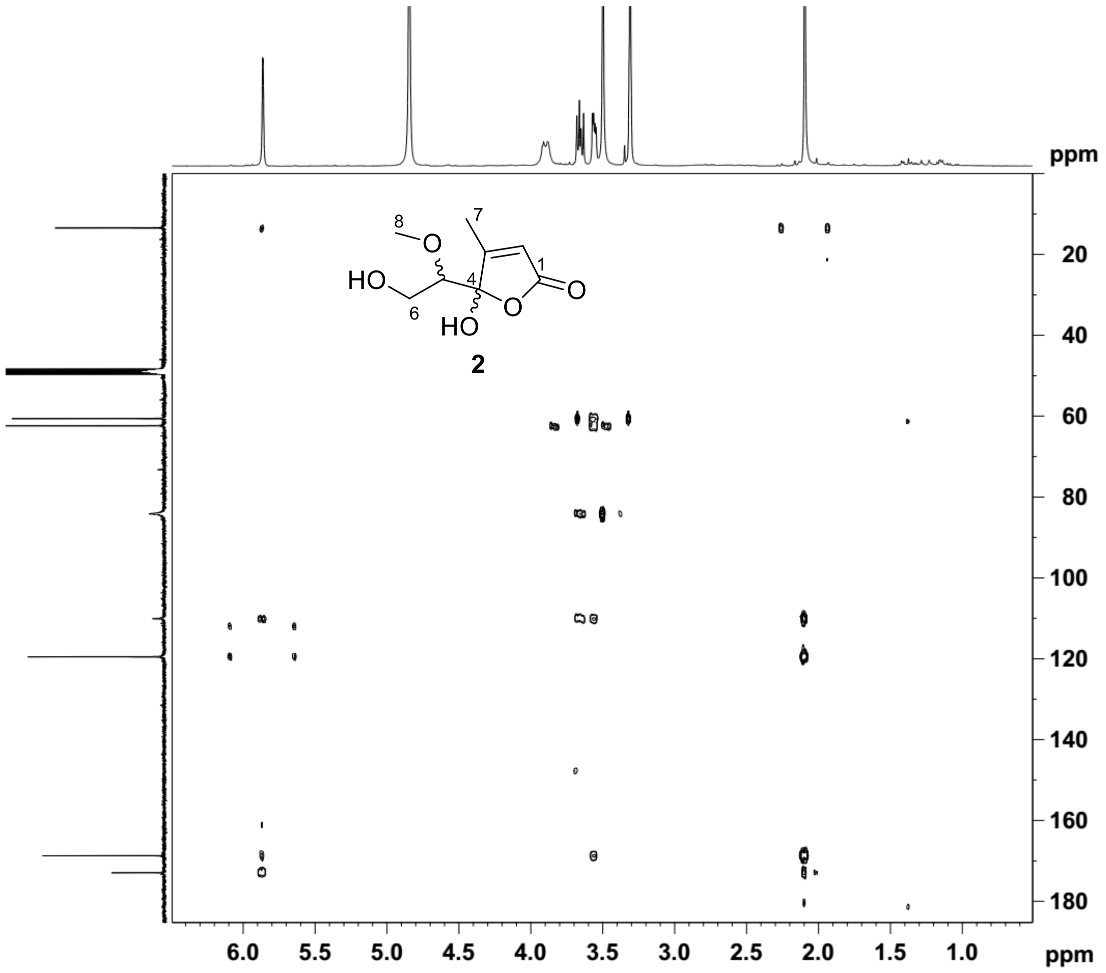

Figure S11. HMBC spectrum of **2** in MeOH-d_4.

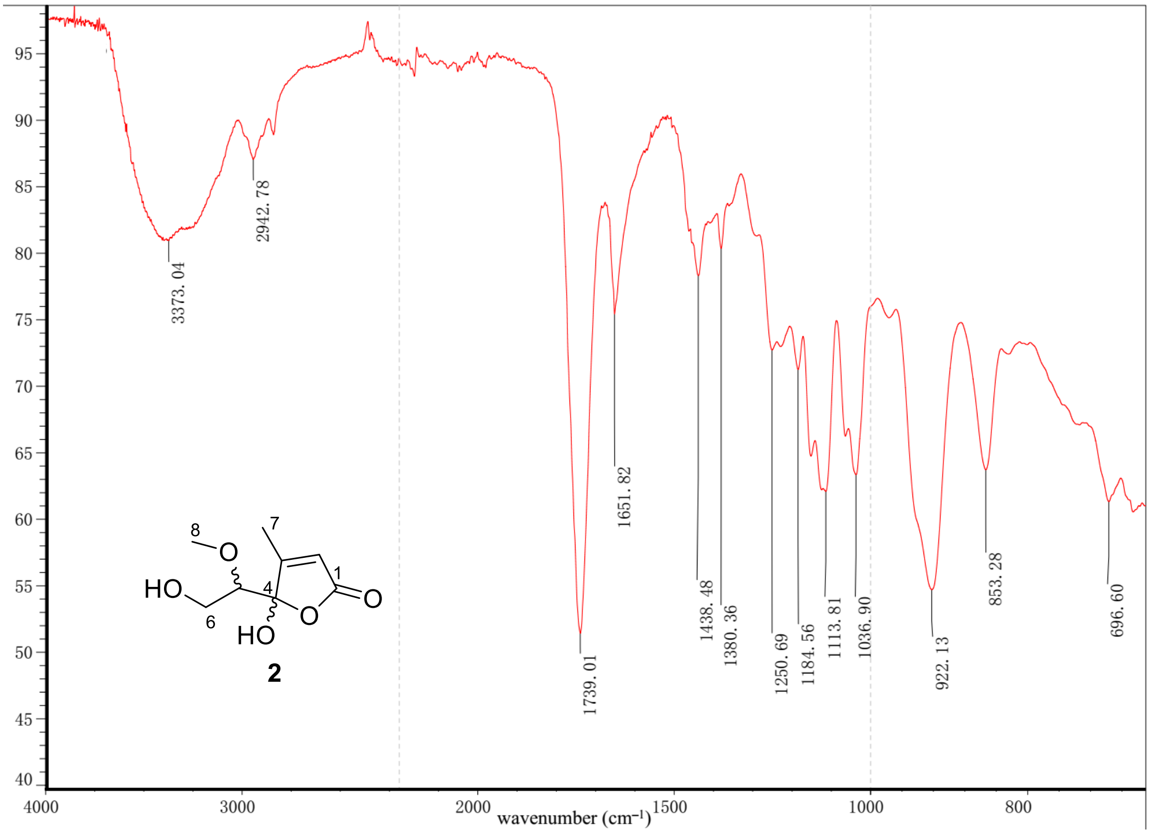

Figure S12. IR spectrum of **2**.

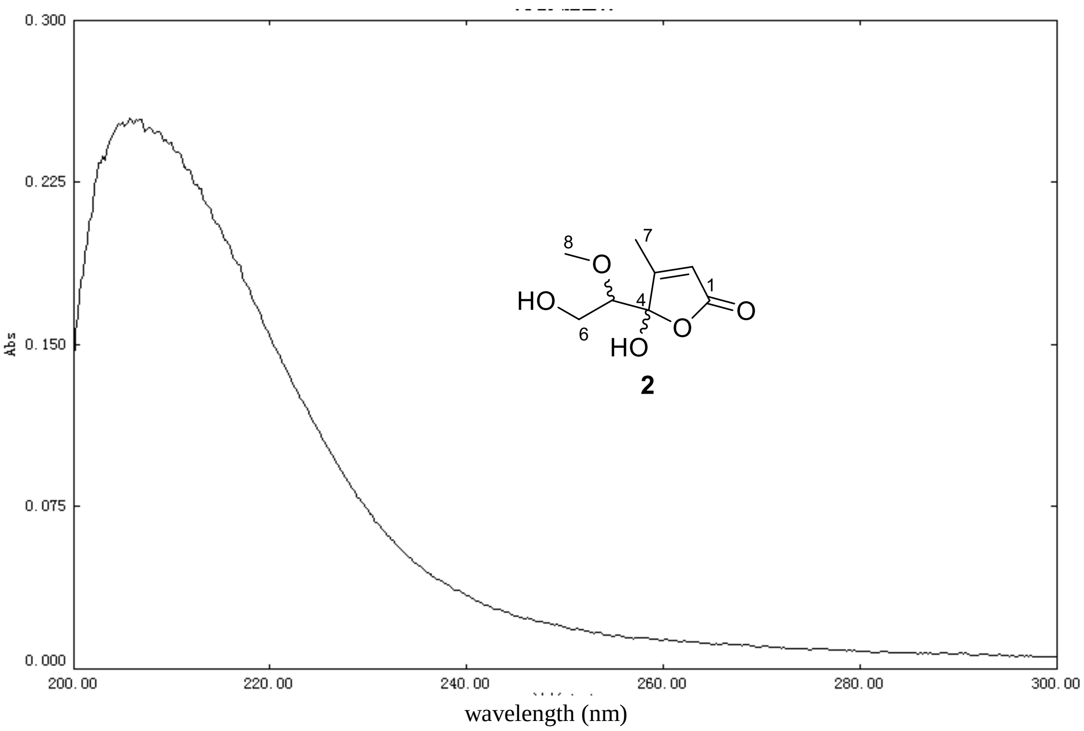

Figure S13. UV spectrum of **2** in MeOH**.**

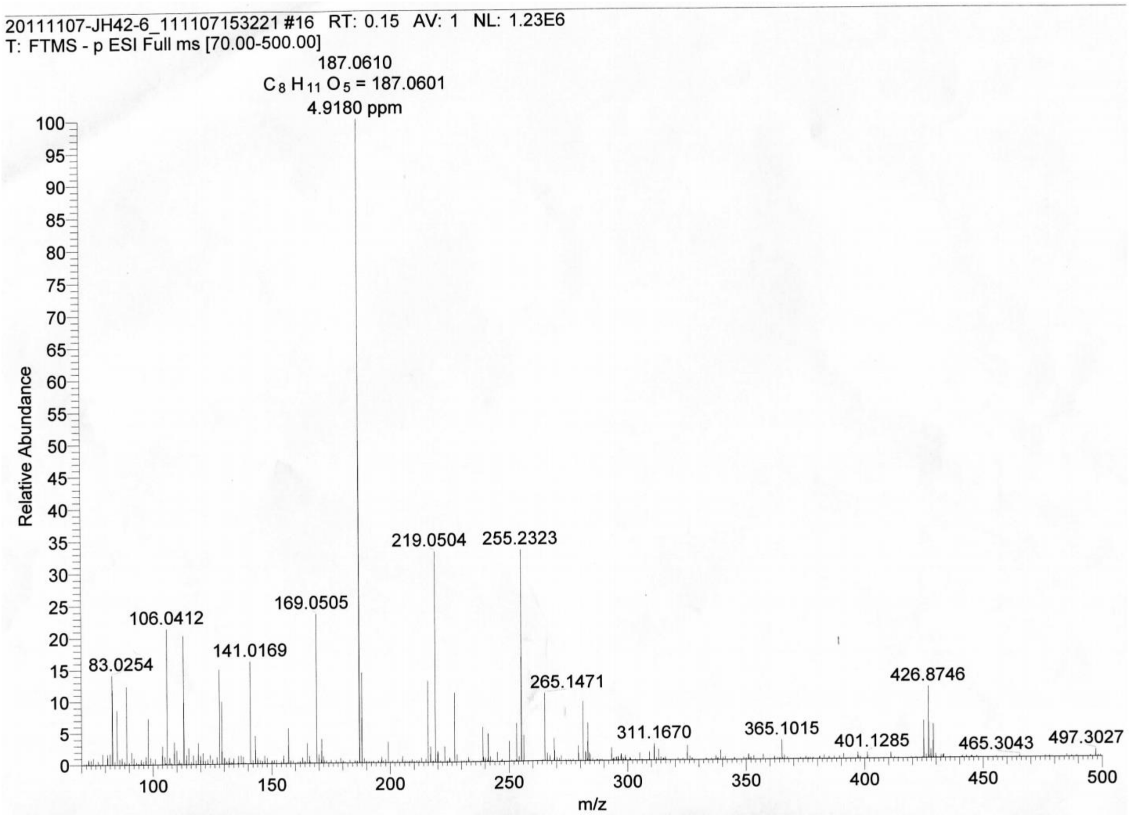

Figure S14. HRESIMS of **2.**

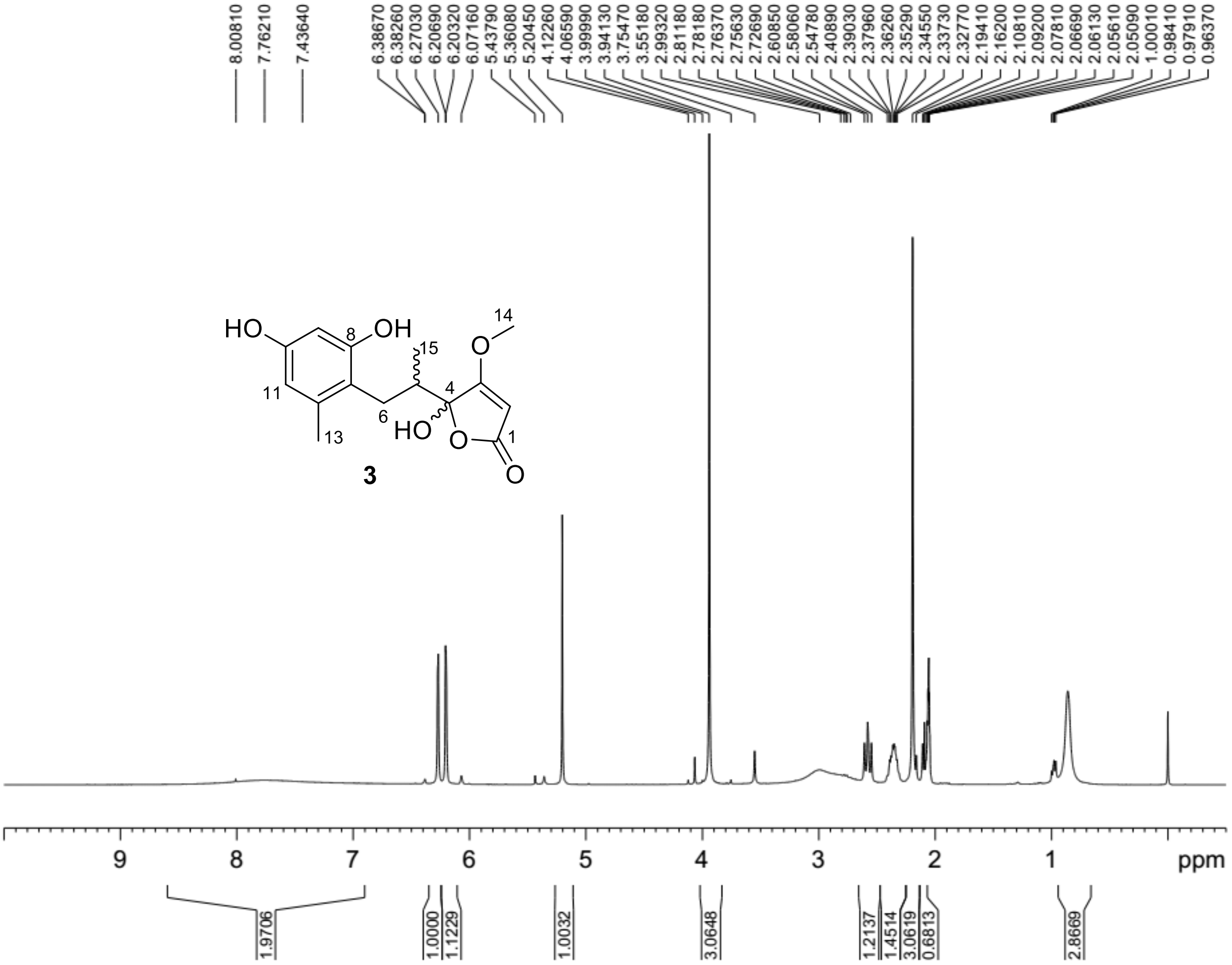

Figure S15. ^{1}H NMR spectrum (400 MHz) of **3** in acetone-d_6.

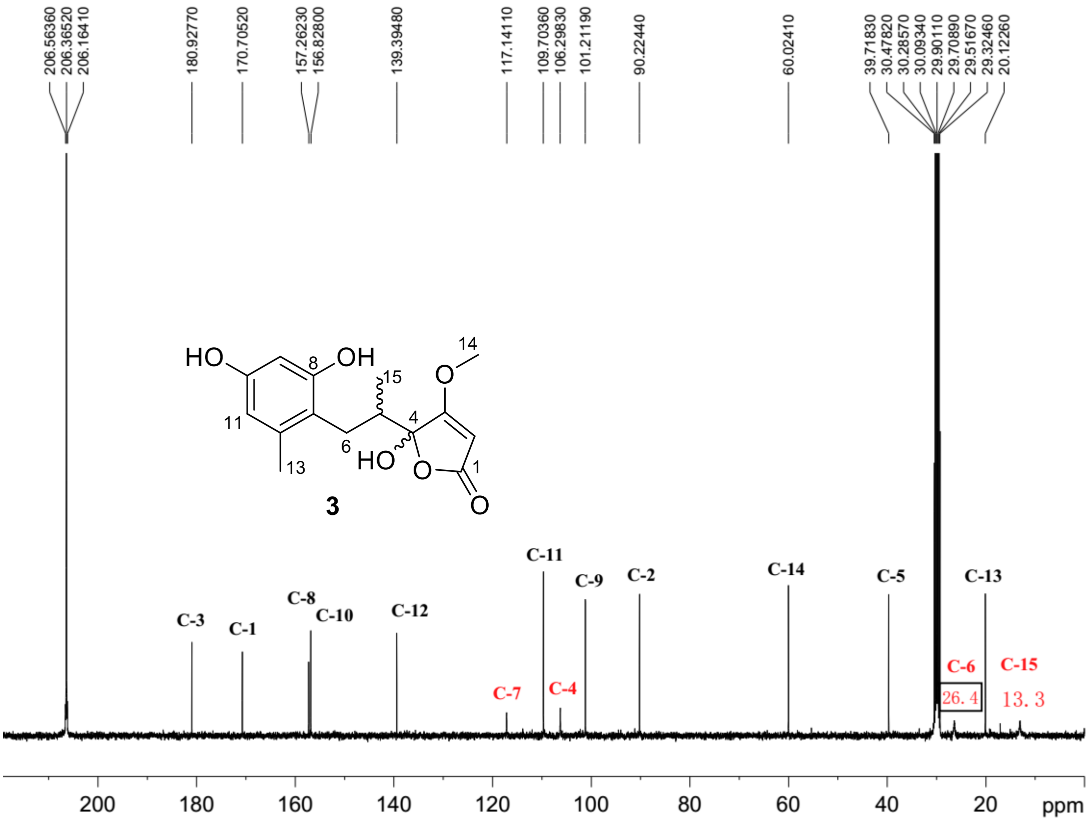

Figure S16. ^{13}C NMR spectrum (100 MHz) of **3** in acetone-d_6.

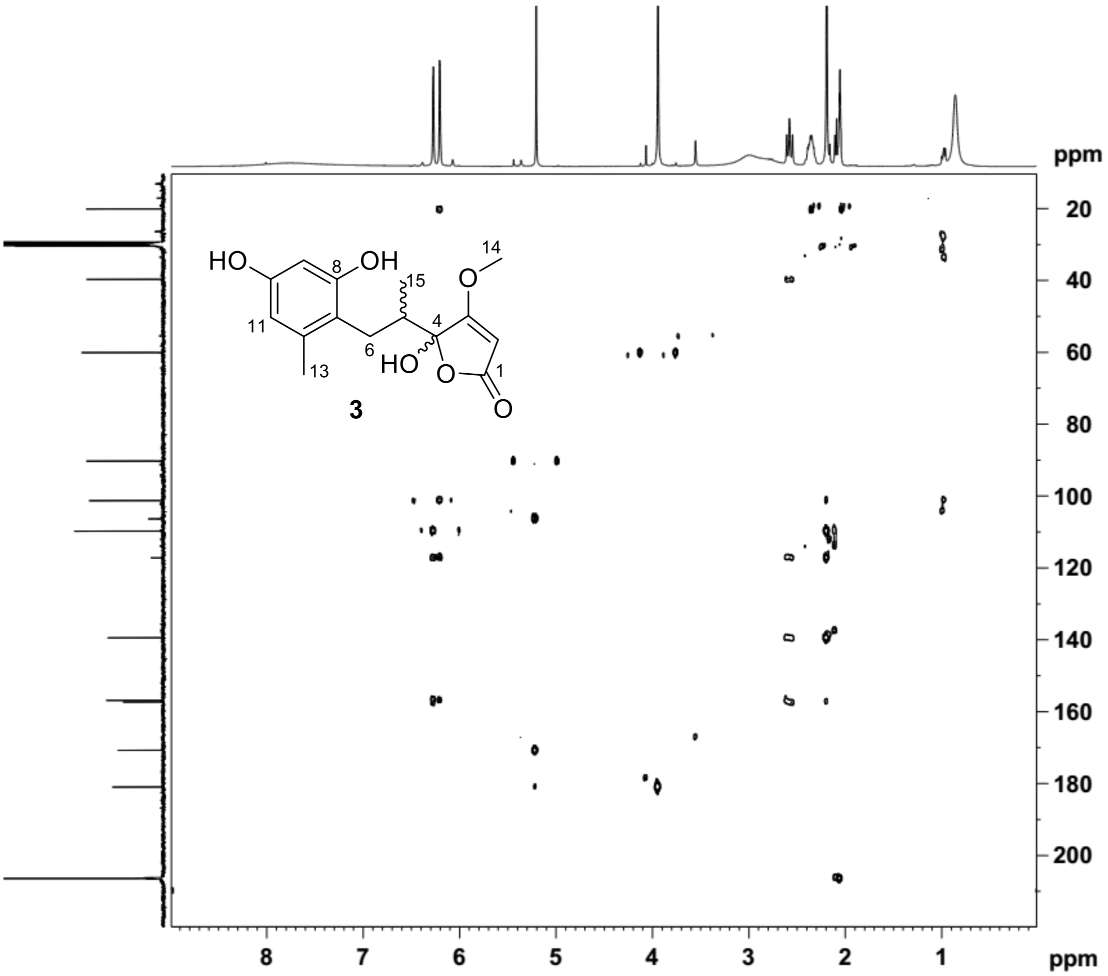

Figure S17. HMBC spectrum of **3** in acetone-d_6.

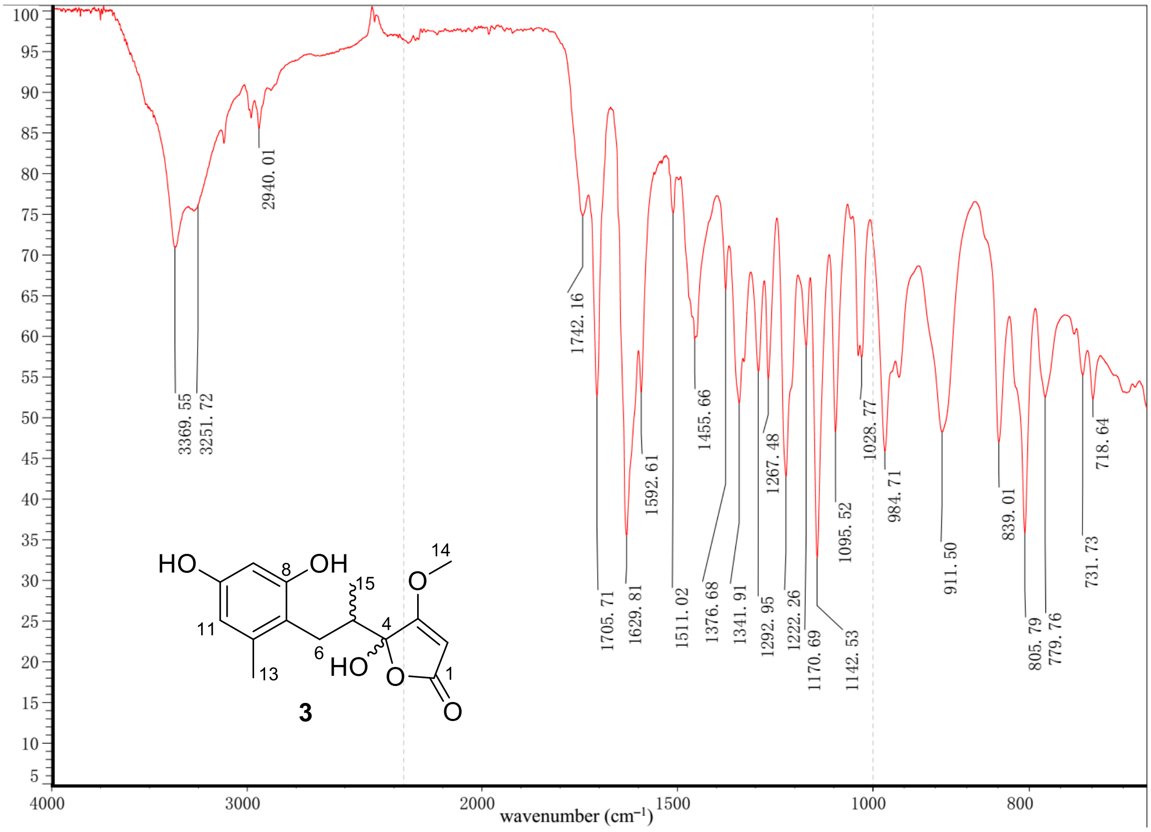

Figure S18. IR spectrum of **3**.

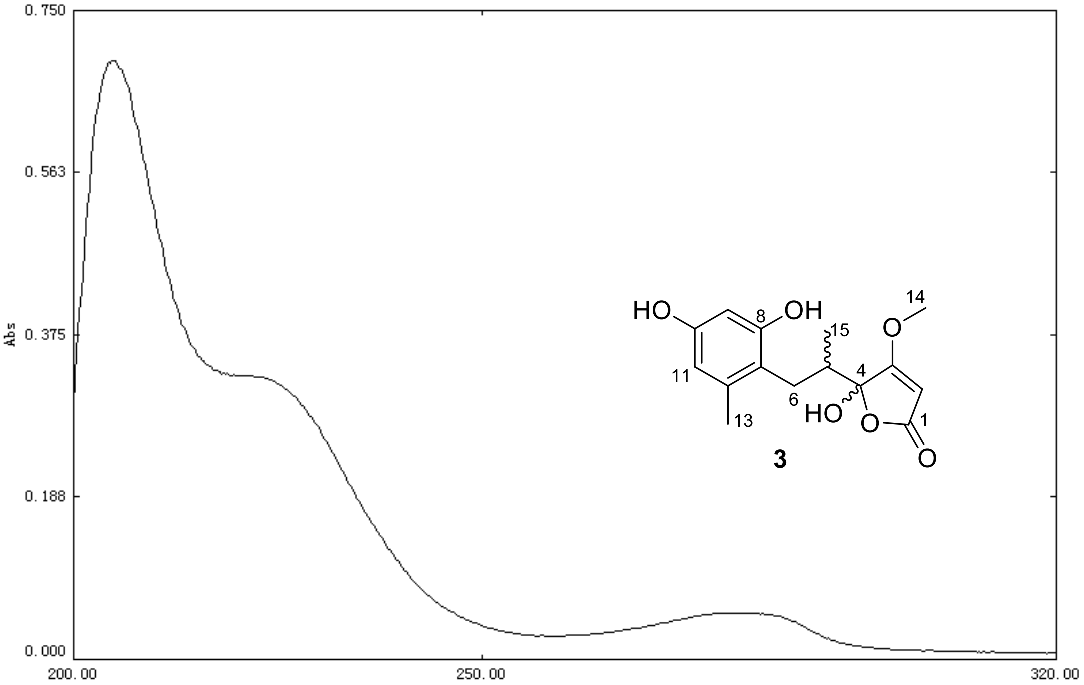

Figure S19. UV spectrum of **3** in MeOH.

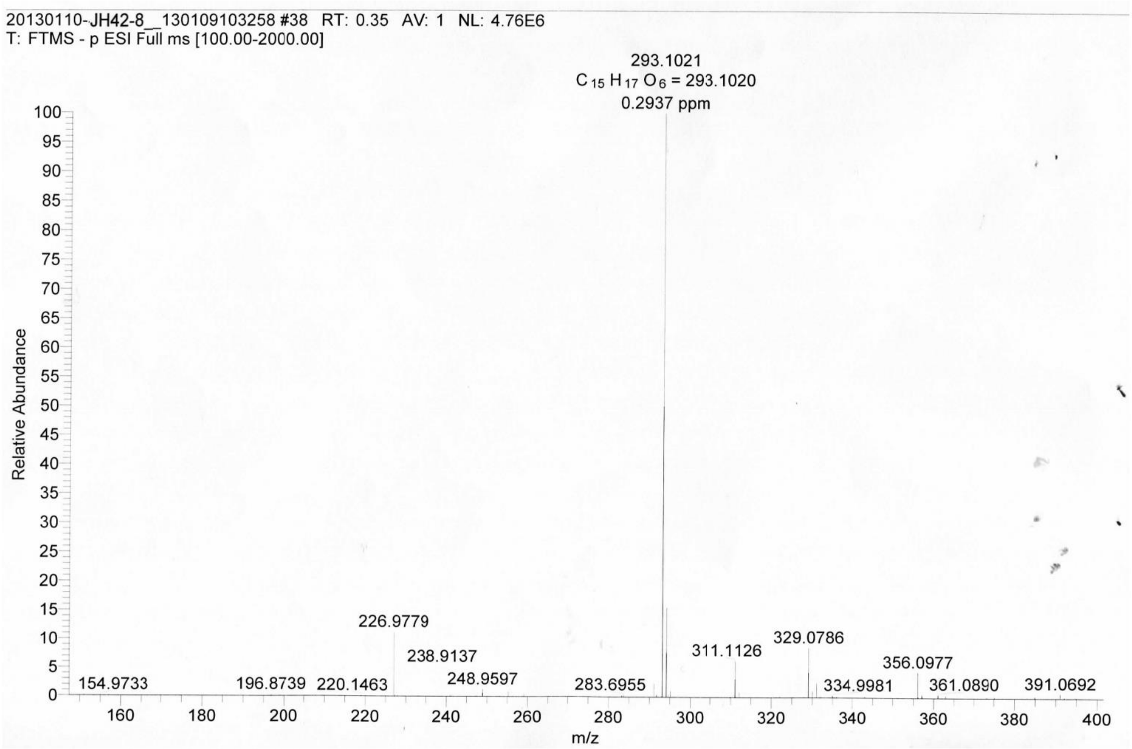

Figure S20. HRESIMS of **3**.

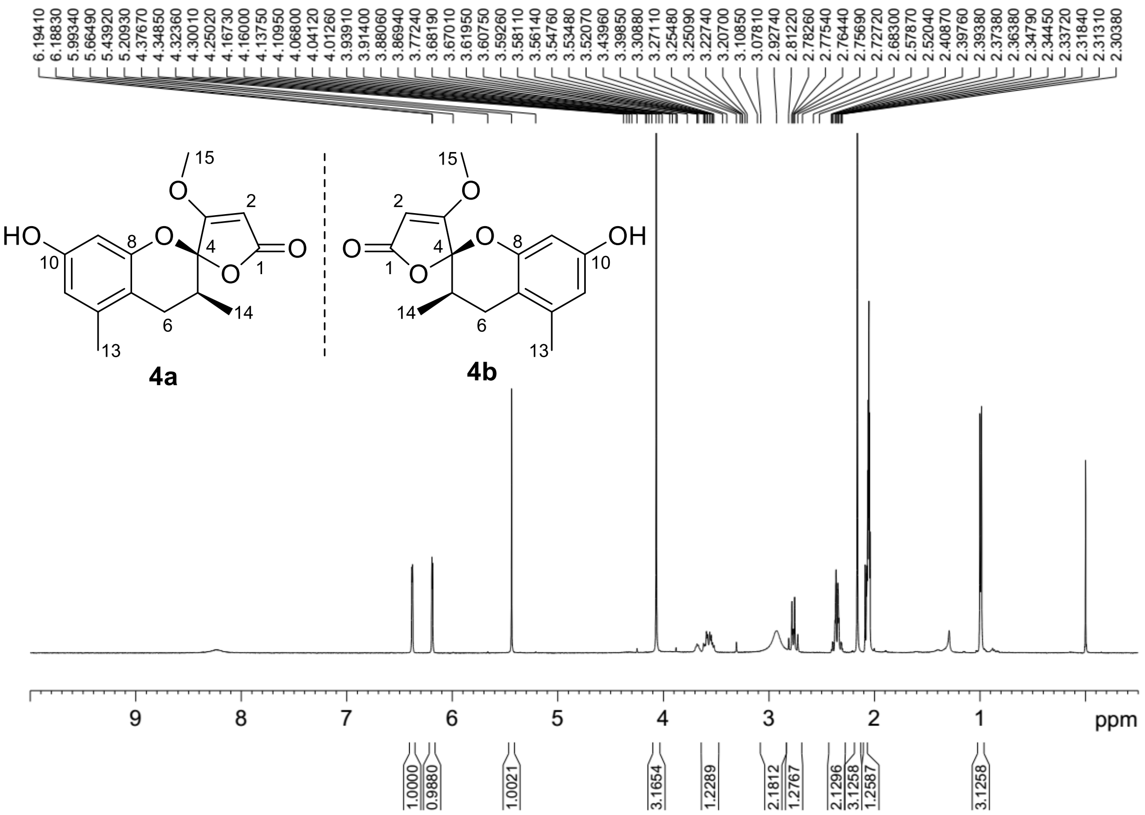

Figure S21. ^{1}H NMR spectrum (400 MHz) of **4** in acetone-d_6.

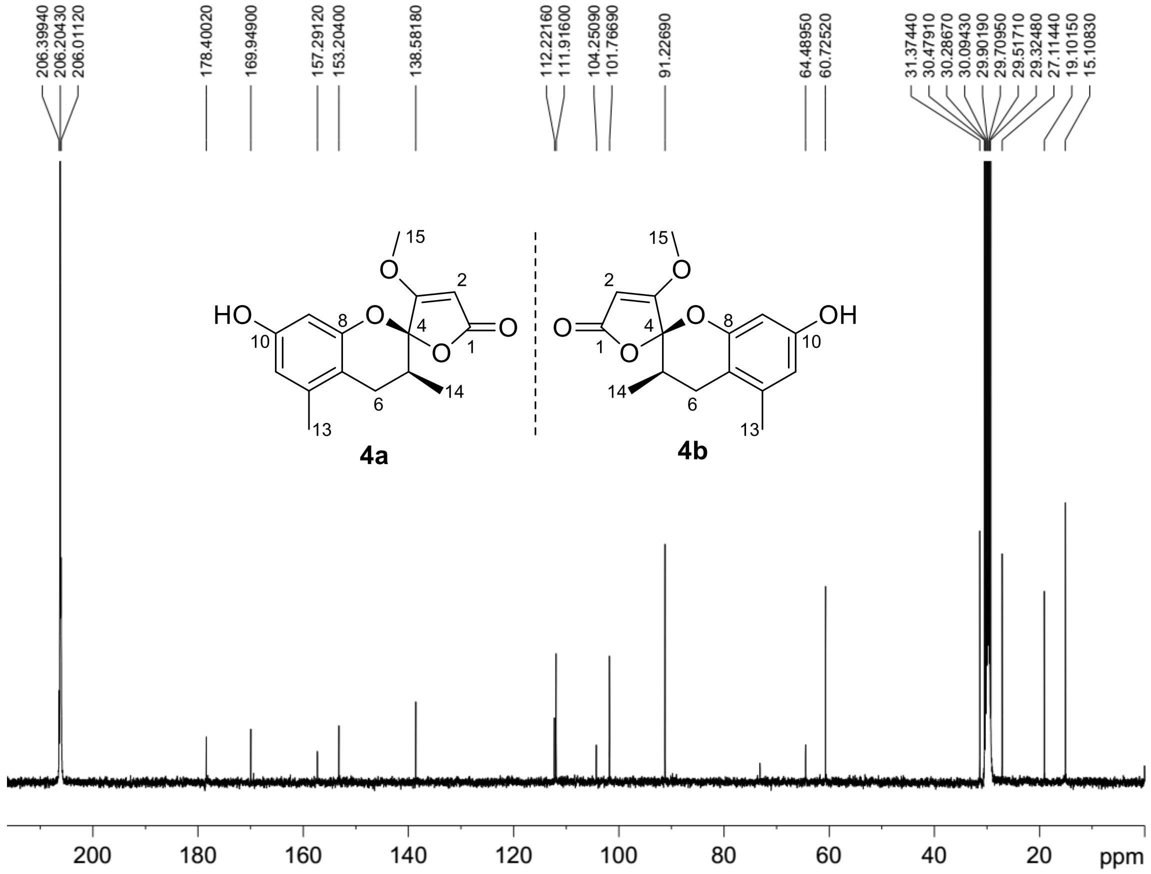

Figure S22. ^{13}C NMR spectrum (100 MHz) of **4** in acetone-d_6**.**

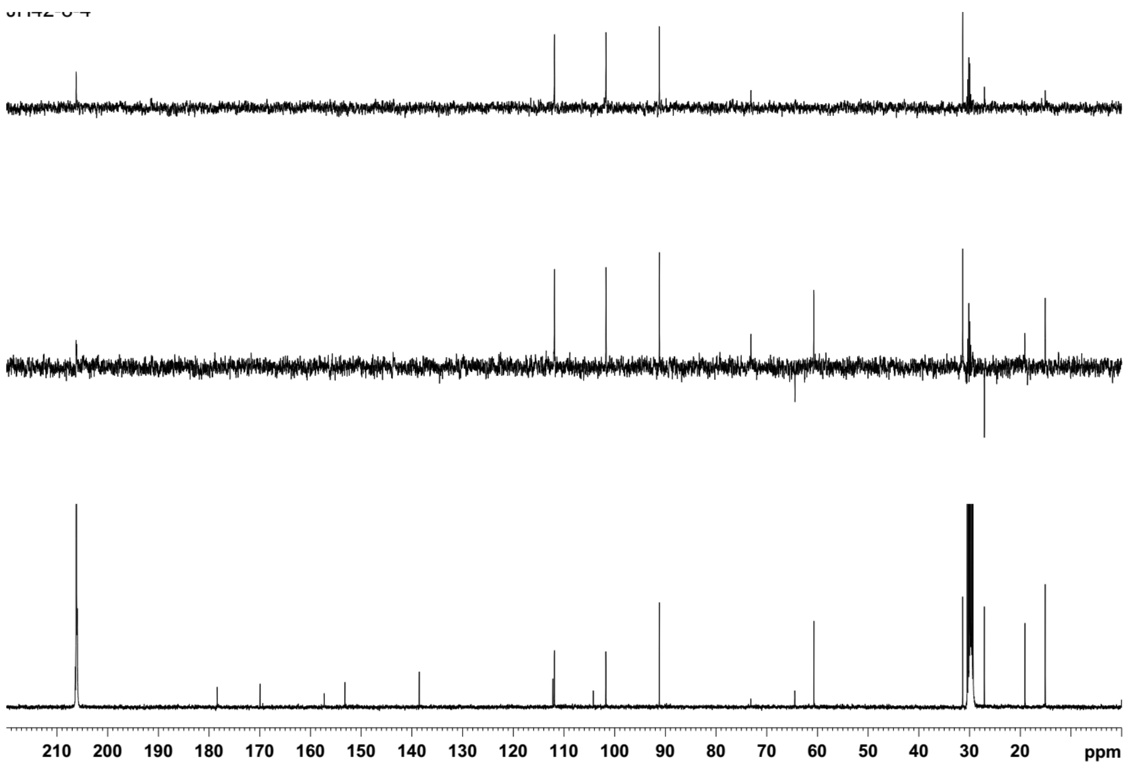

Figure S23. DEPT spectrum of **4** in acetone-d_6.

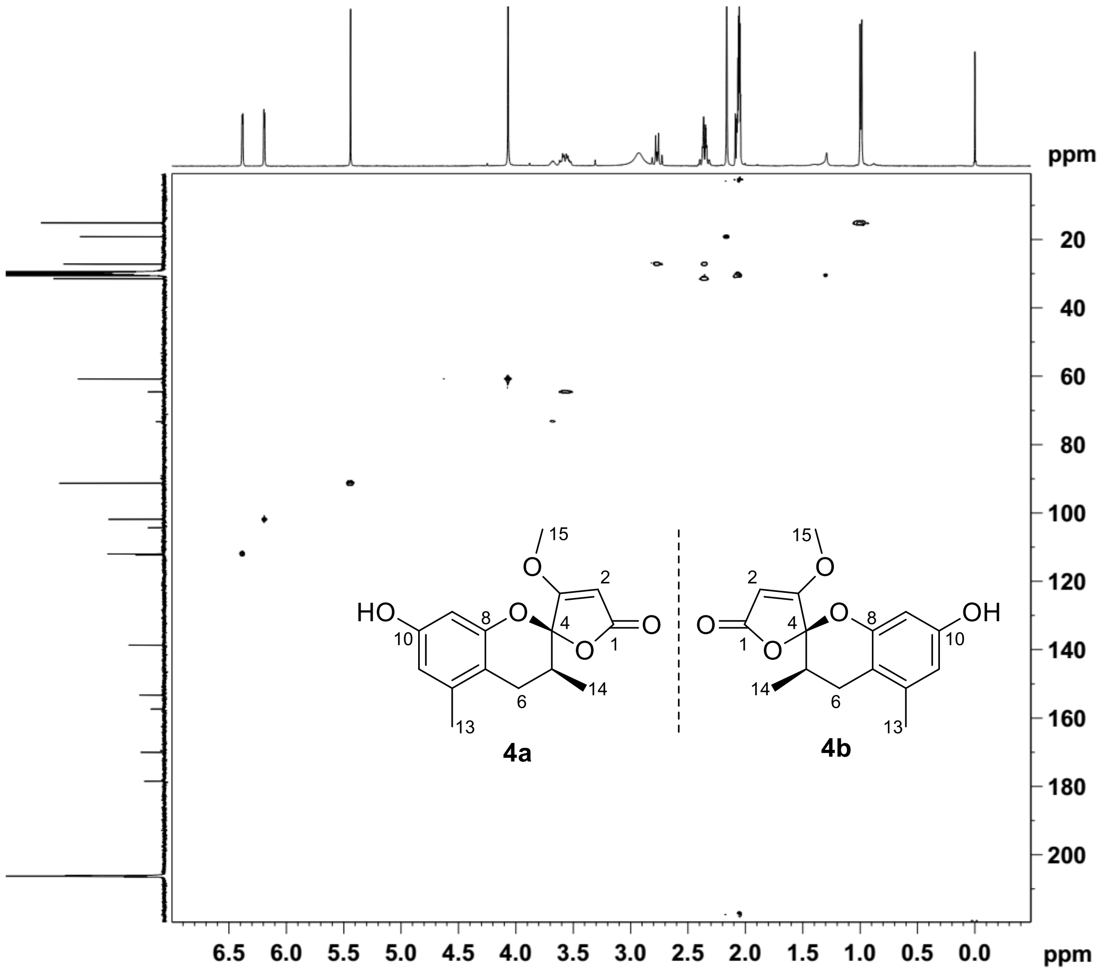

Figure S24. HSQC spectrumof **4** in acetone-d_6.

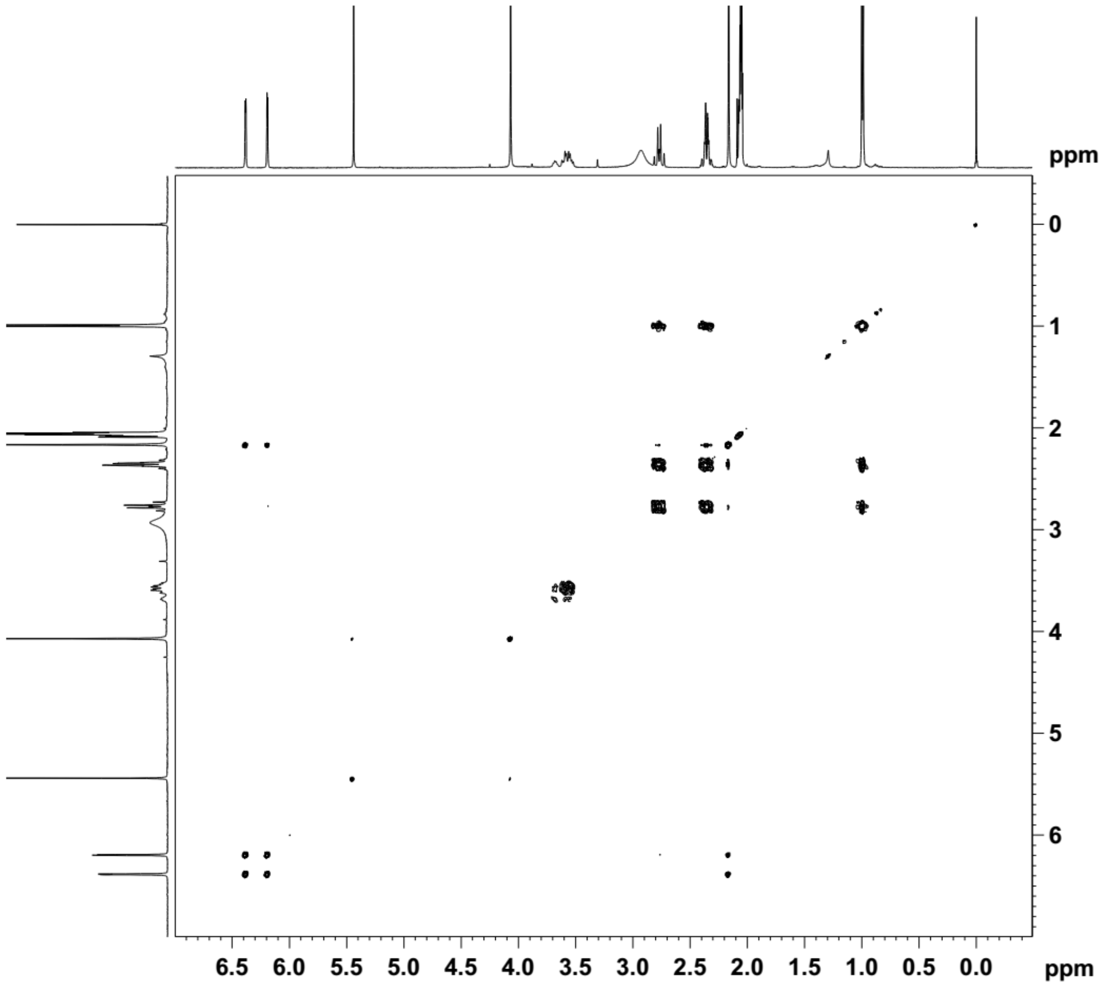

Figure S25. ^{1}H-^{1}H COSY spectrum of **4** in acetone-d_6.

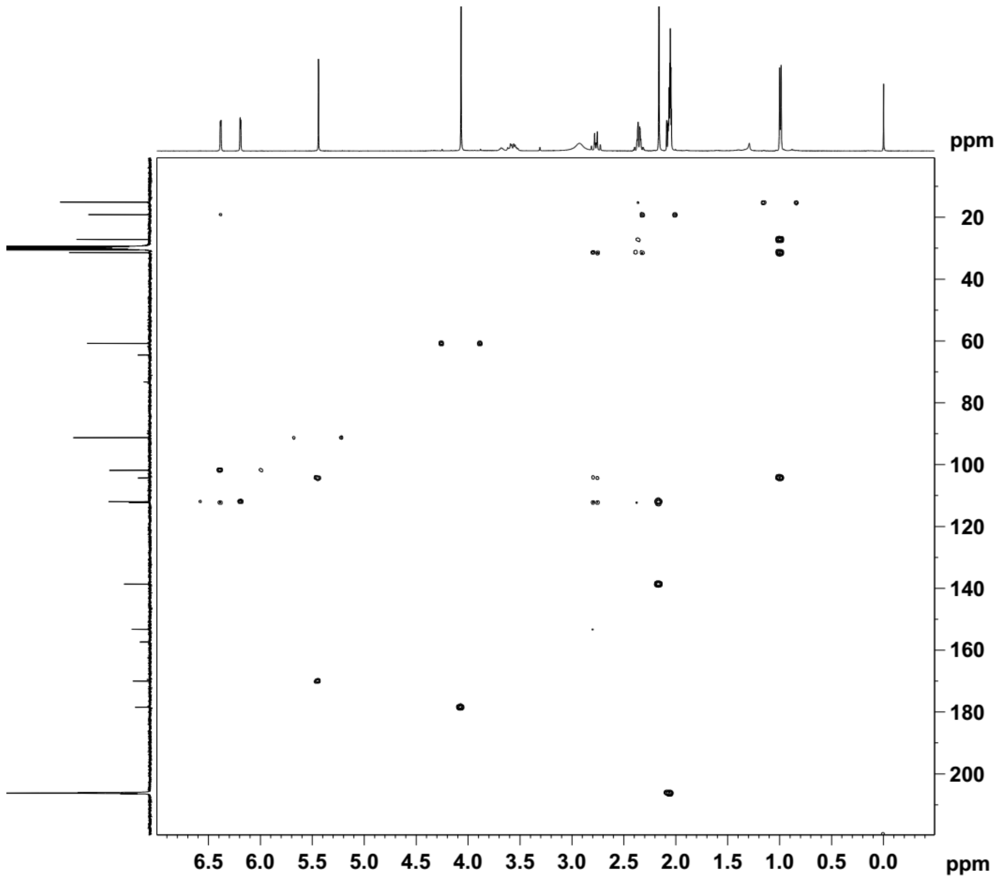

Figure S26. HMBC spectrum of **4** in acetone-d_6.

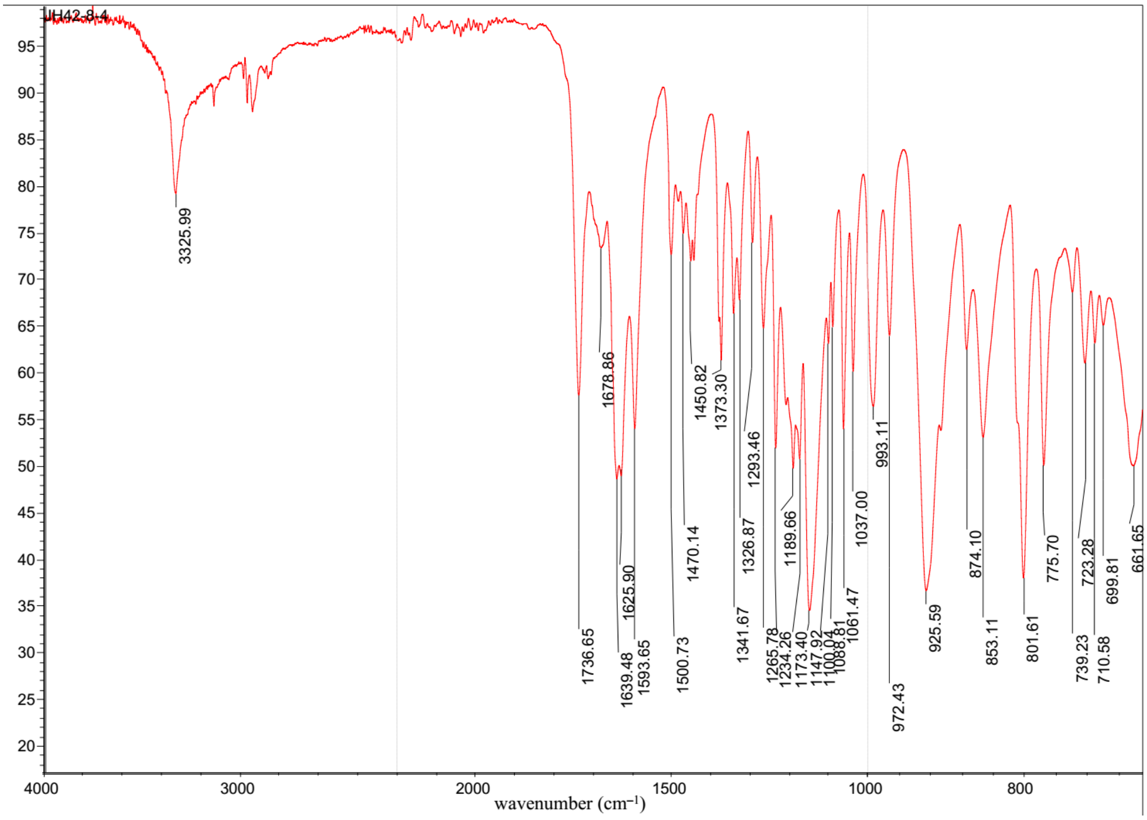

Figure S27. IR spectrum of **4**.

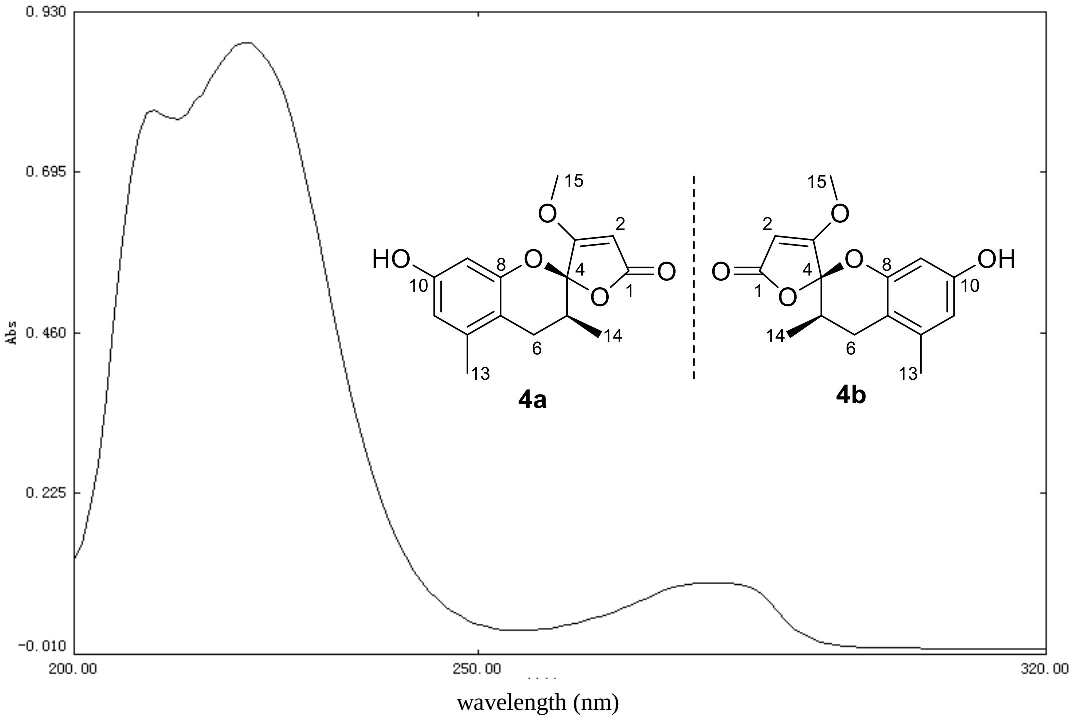

Figure S28. UV spectrum of **4** in MeOH.

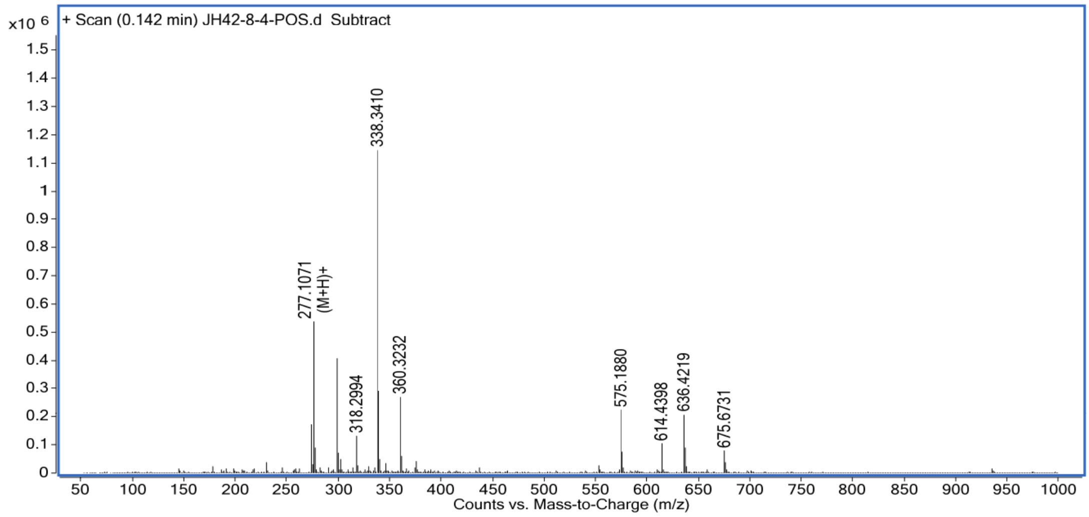

Name	Formula	Score	Mass	Mass (MFG)	Diff (ppm)	Diff (abs. ppm)	Diff (mDa)
	C15 H16 O5	99.37	276.0998	276.0998	-0.03	0.03	-0.01

Species	Ion Formula	m/z	Height	Score (MFG)	Score (MFG, MS)	Score (MFG, mass)	Score (MFG, abund)	Score (MFG, iso. spacing)
(M+H)+	C15 H17 O5	277.1071	535175.6	99.37	99.37	100	99.91	97.46

m/z	m/z (Calc)	Diff (ppm)	Diff (mDa)	Height	Height (Calc)	Height %	Height % (Calc)	Height Sum %	Height Sum% (Calc)
277.1071	277.1071	-0.04	0	535175.6	534049	100	100	84.3	84.1
278.1099	278.1104	1.86	0.5	88073.8	88703.3	16.5	16.6	13.9	14
279.1151	279.1127	-8.45	-2.4	11888.1	12385.2	2.2	2.3	1.9	1.9

Figure S29. HRESIMS of **4**.

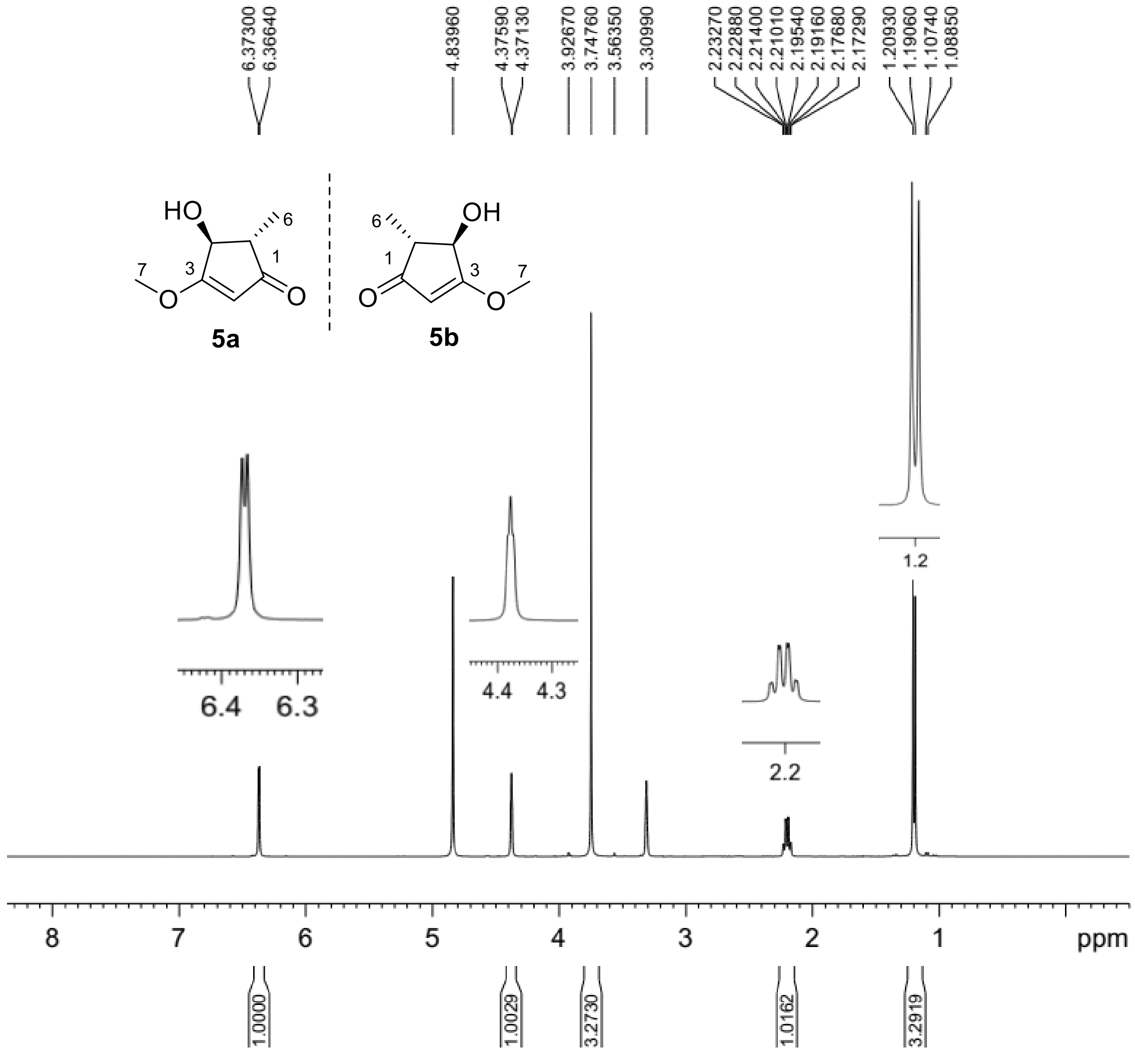

Figure S30. ^{1}H NMR spectrum (400 MHz) of **5** in MeOH-d_4.

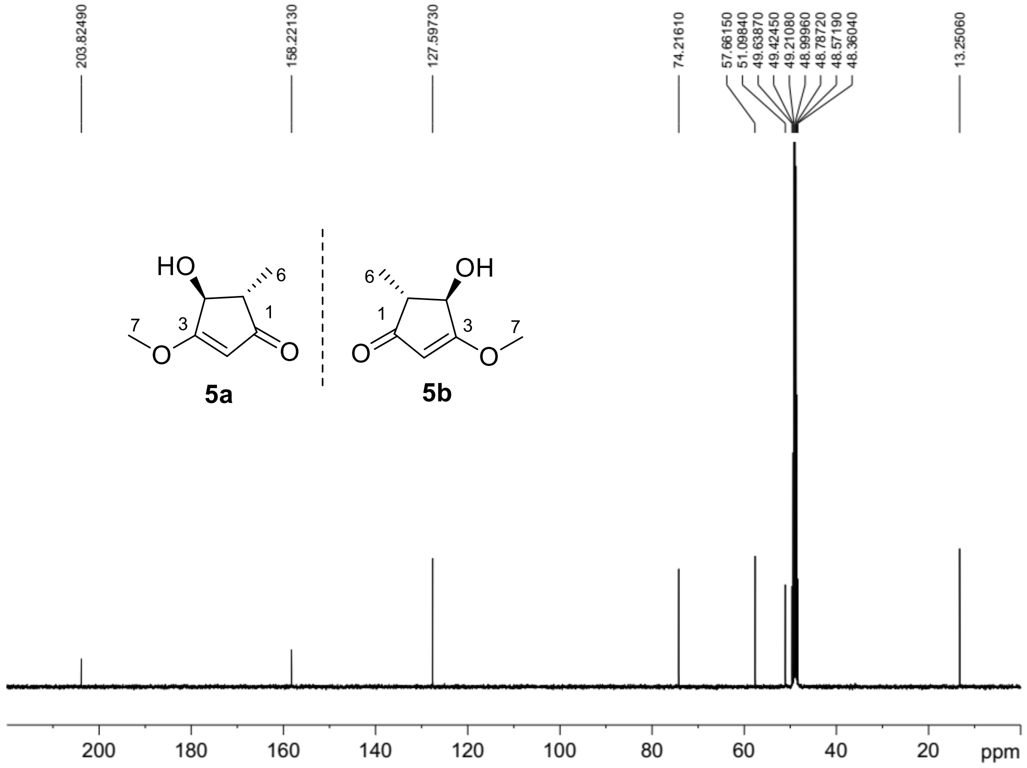

Figure S31. ^{13}C NMR spectrum (100 MHz) of **5** in MeOH-d_4.

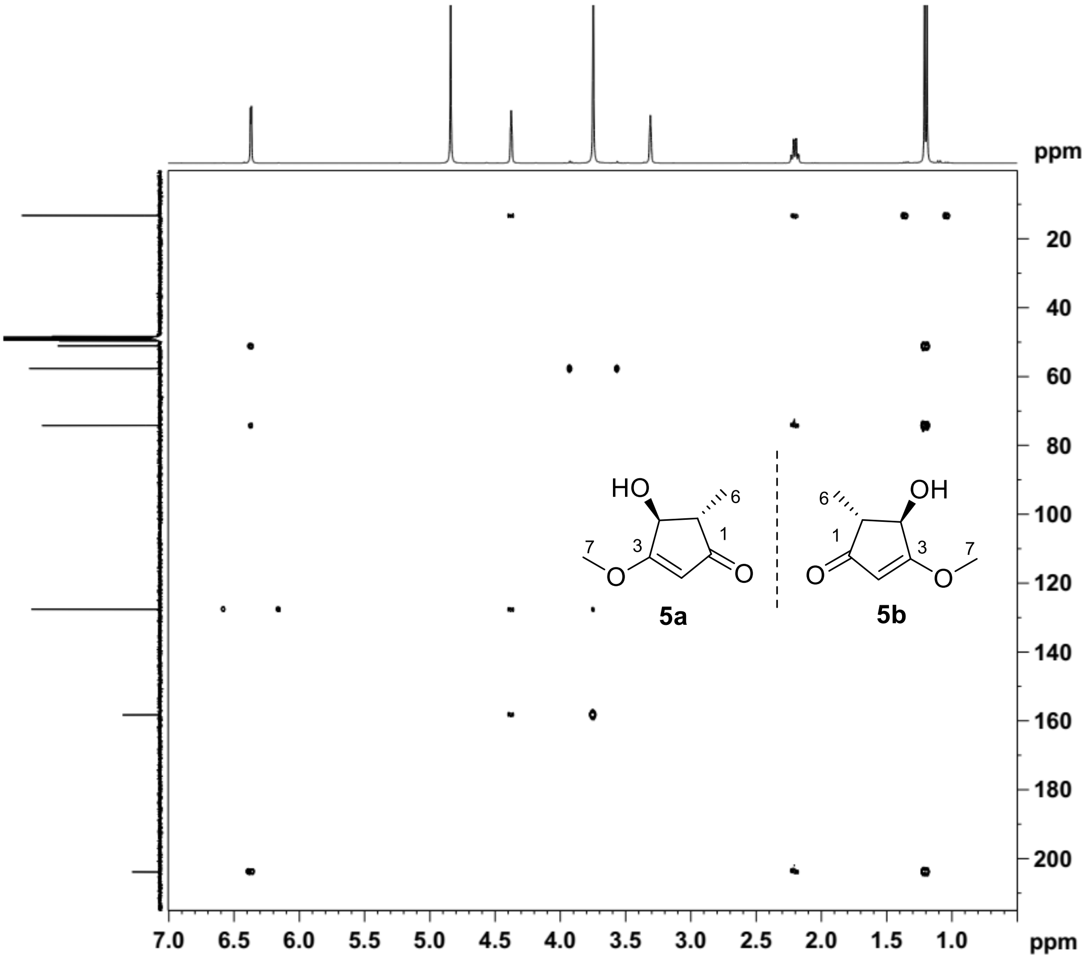

Figure S32. HMBC spectrum of **5** in MeOH-d_4.

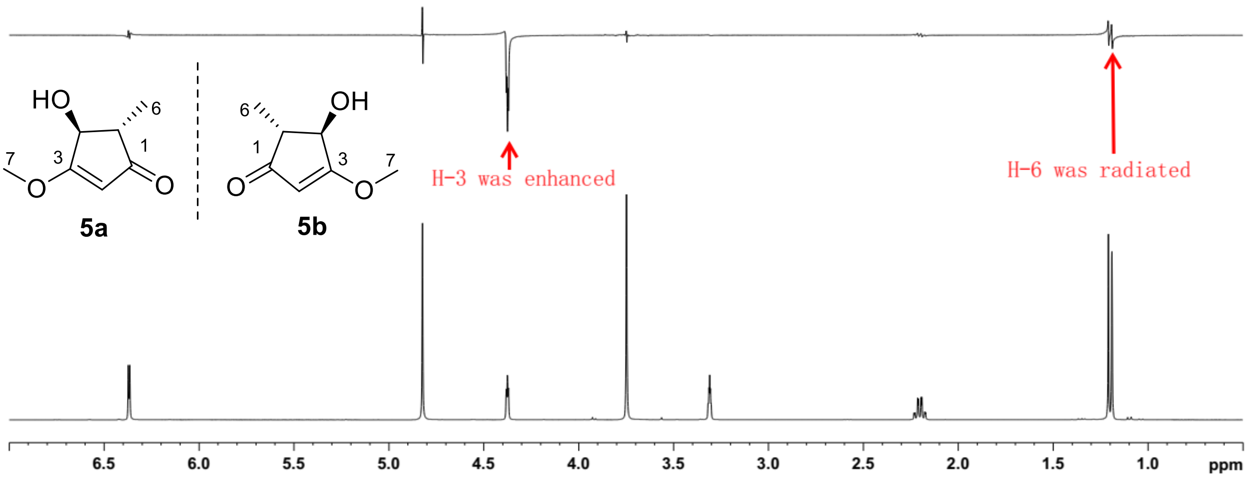

Figure S33. NOE difference spectrum of **5** in MeOH-d_4.

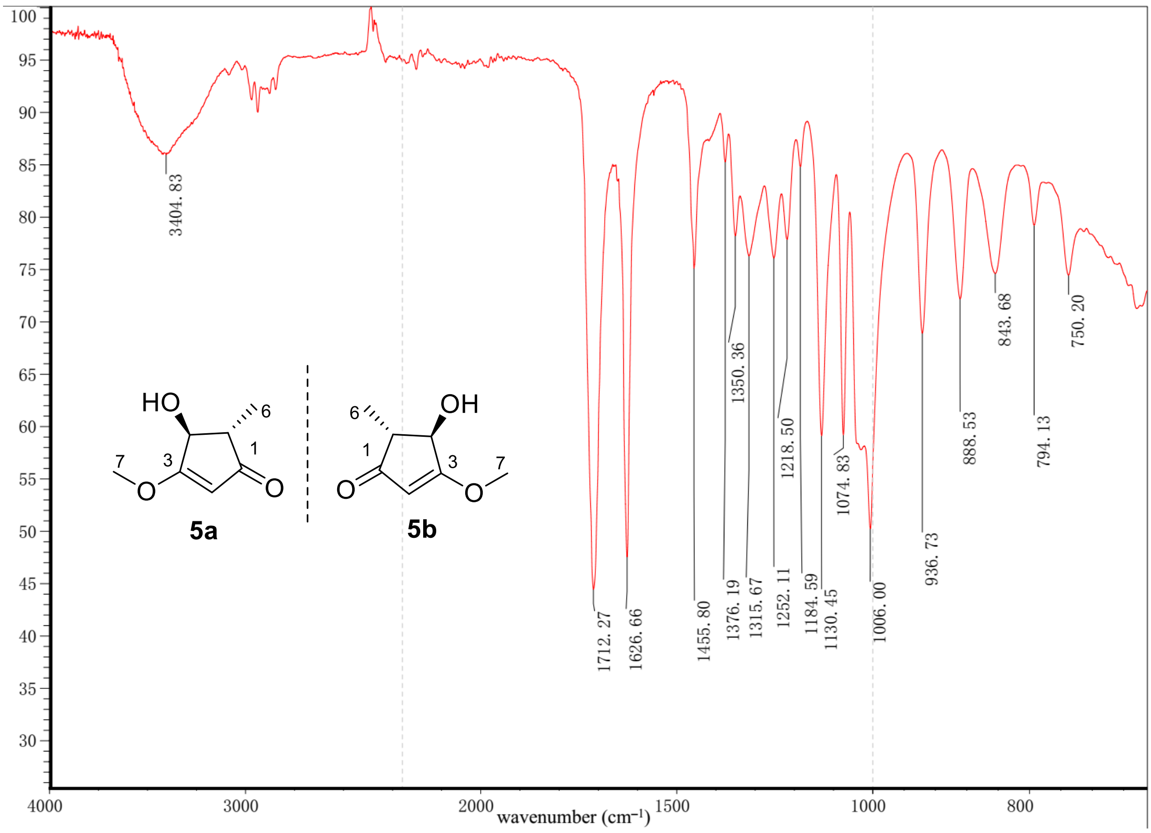

Figure S34. IR spectrum of **5**.

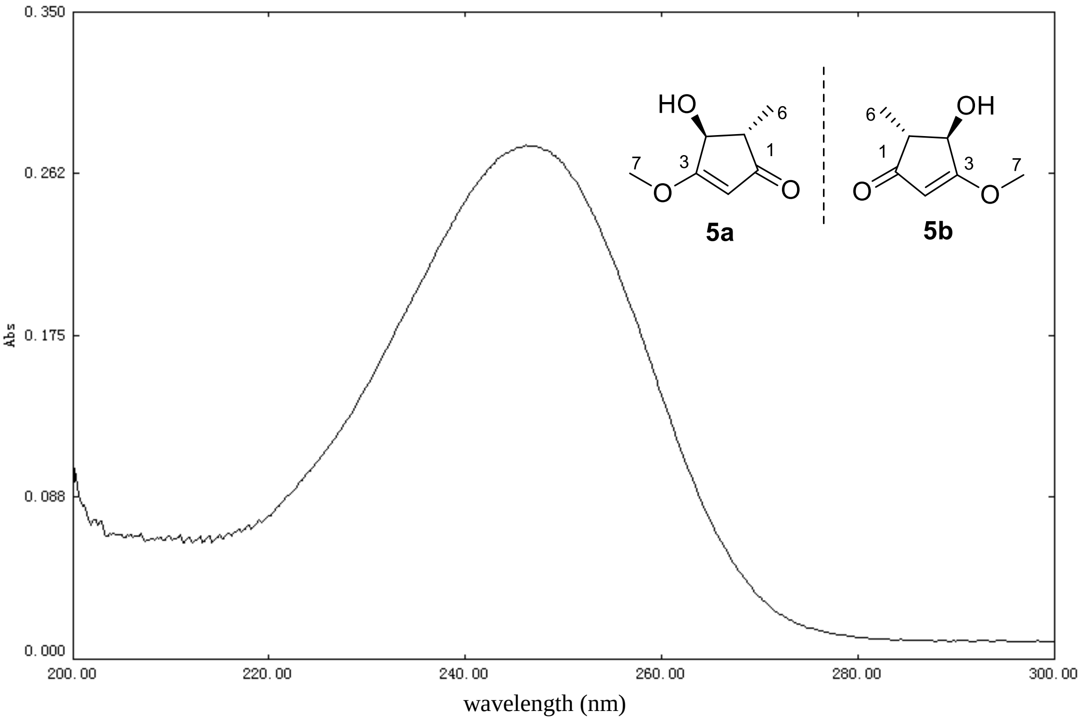

Figure S35. UV spectrum of **5** in MeOH.

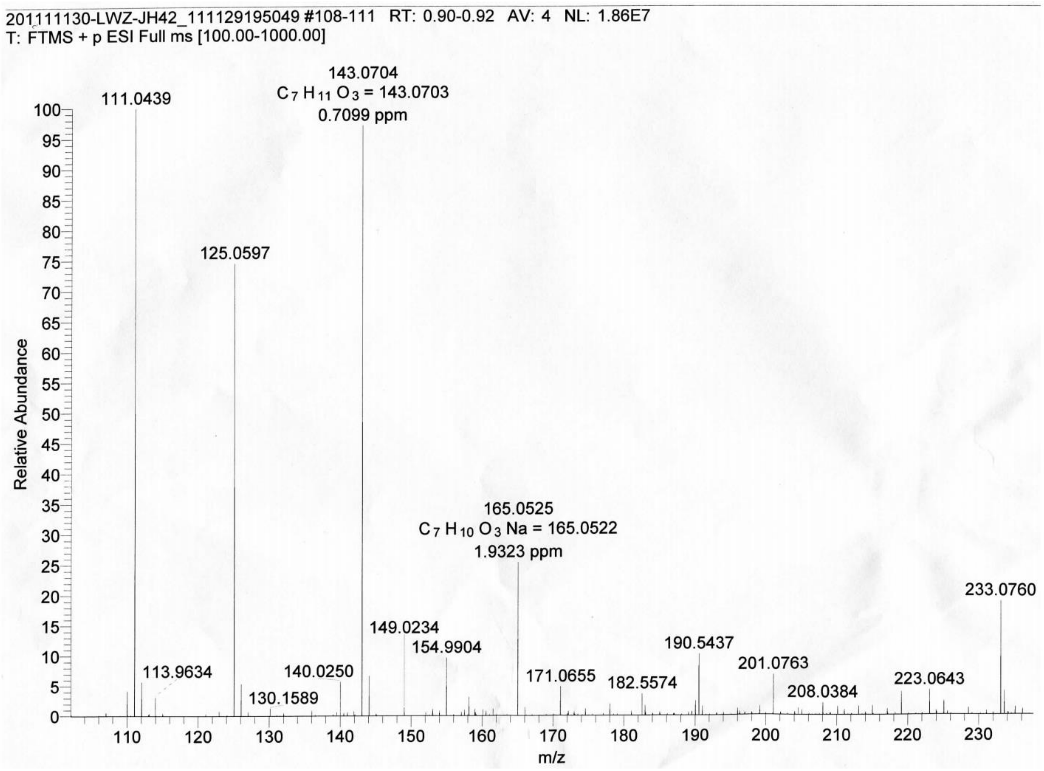

Figure S36. HRESIMS of **5**.

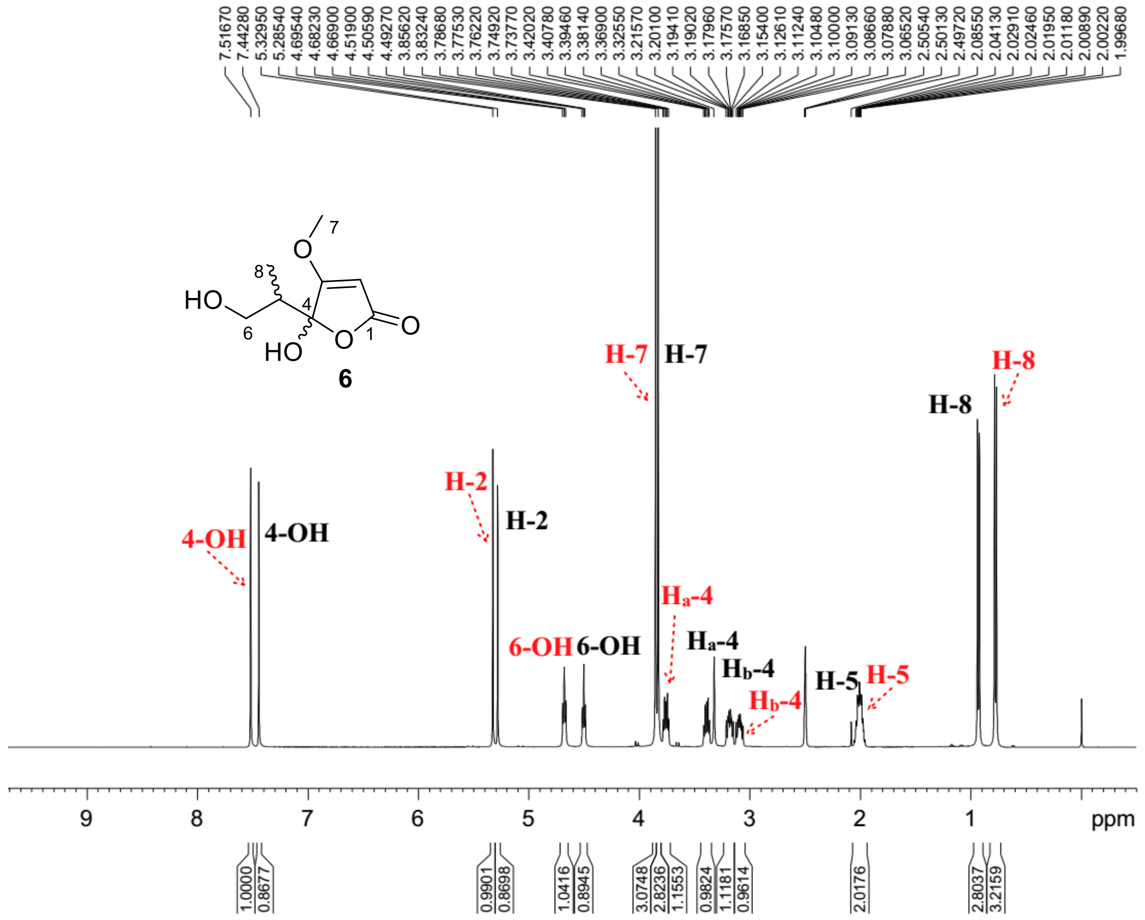

Figure S37. ^{1}H NMR spectrum (400 MHz) of **6** in DMSO-d_6.

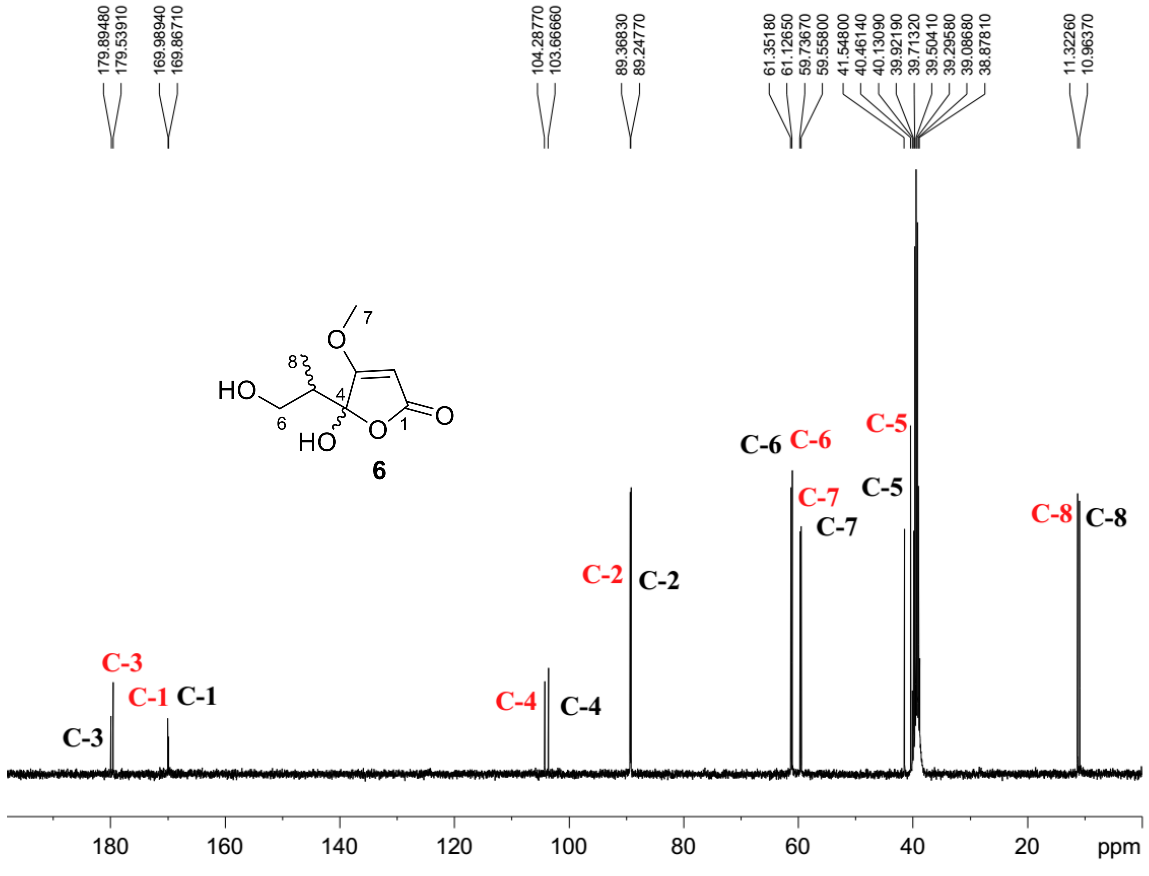

Figure S38. ^{13}C NMR spectrum (100 MHz) of **6** in DMSO-d_4.

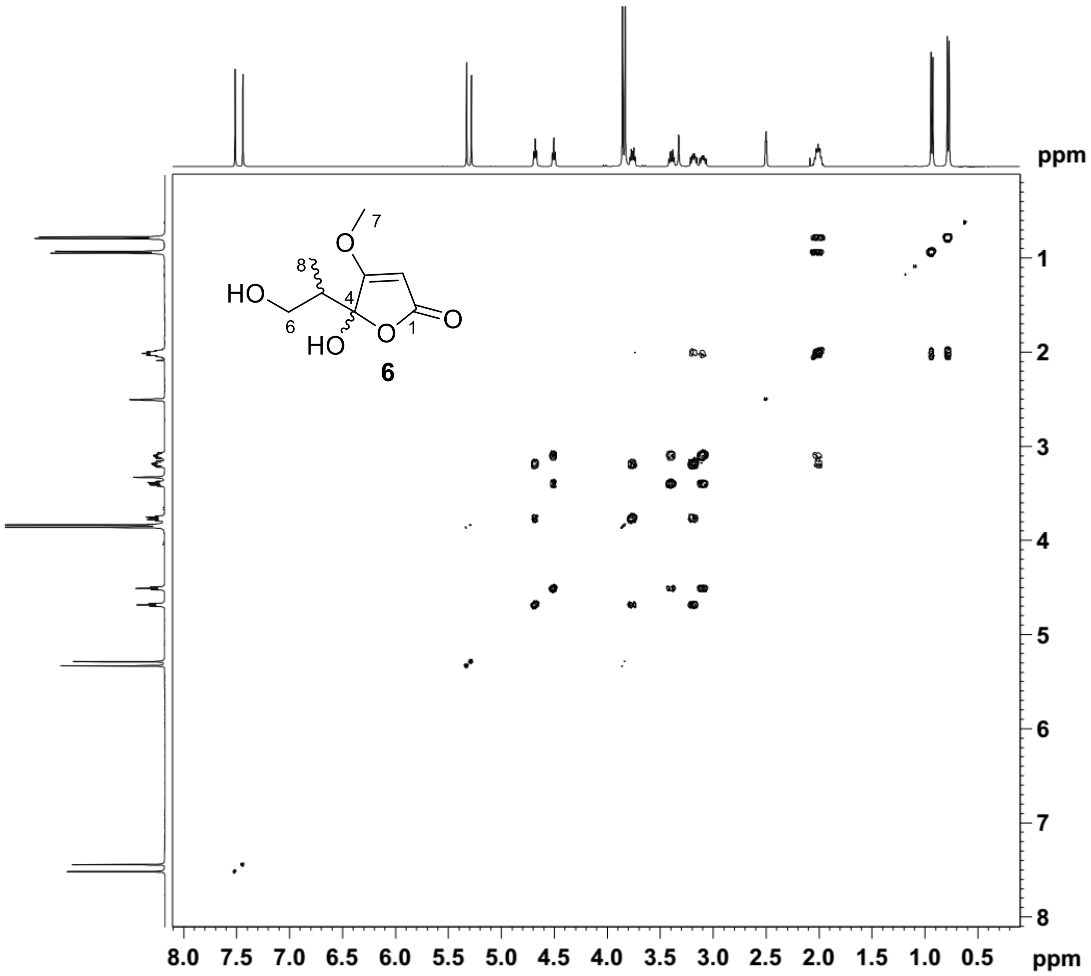

Figure S39. ^{1}H-^{1}H COSY spectrum of 6 in DMSO-d_6.

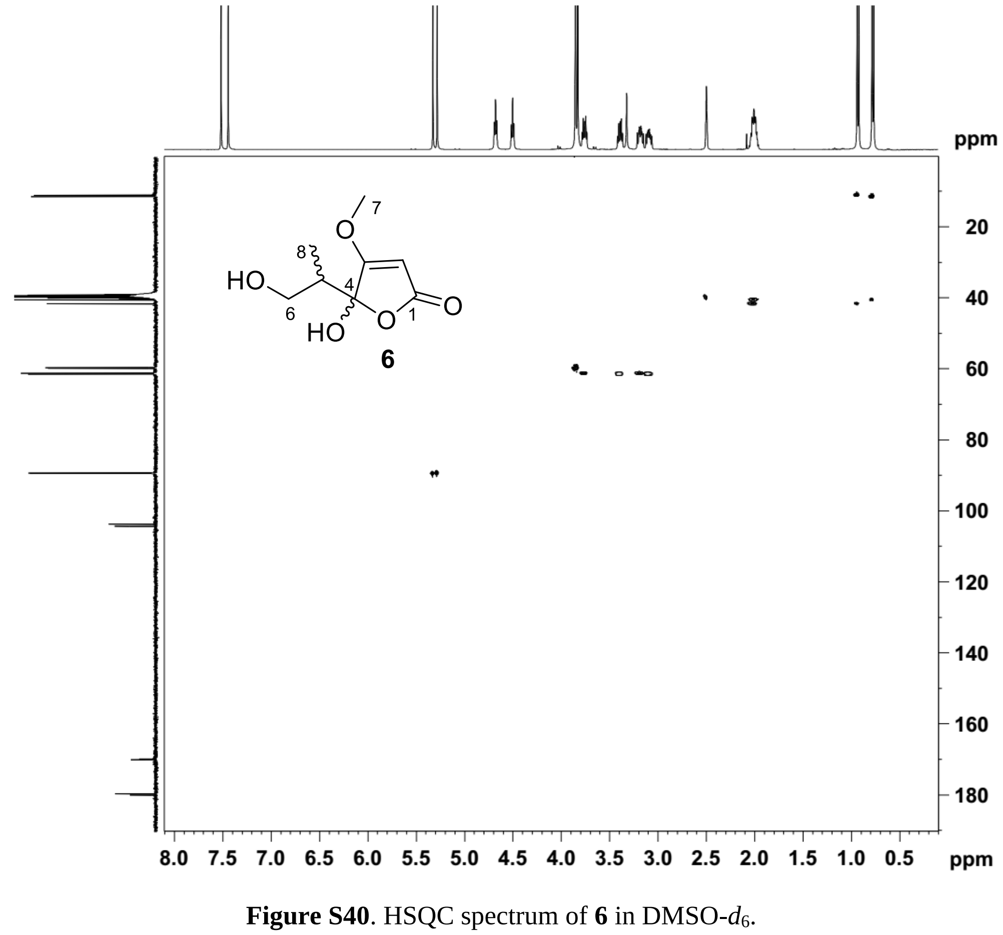

Figure S40. HSQC spectrum of **6** in DMSO-d_6.

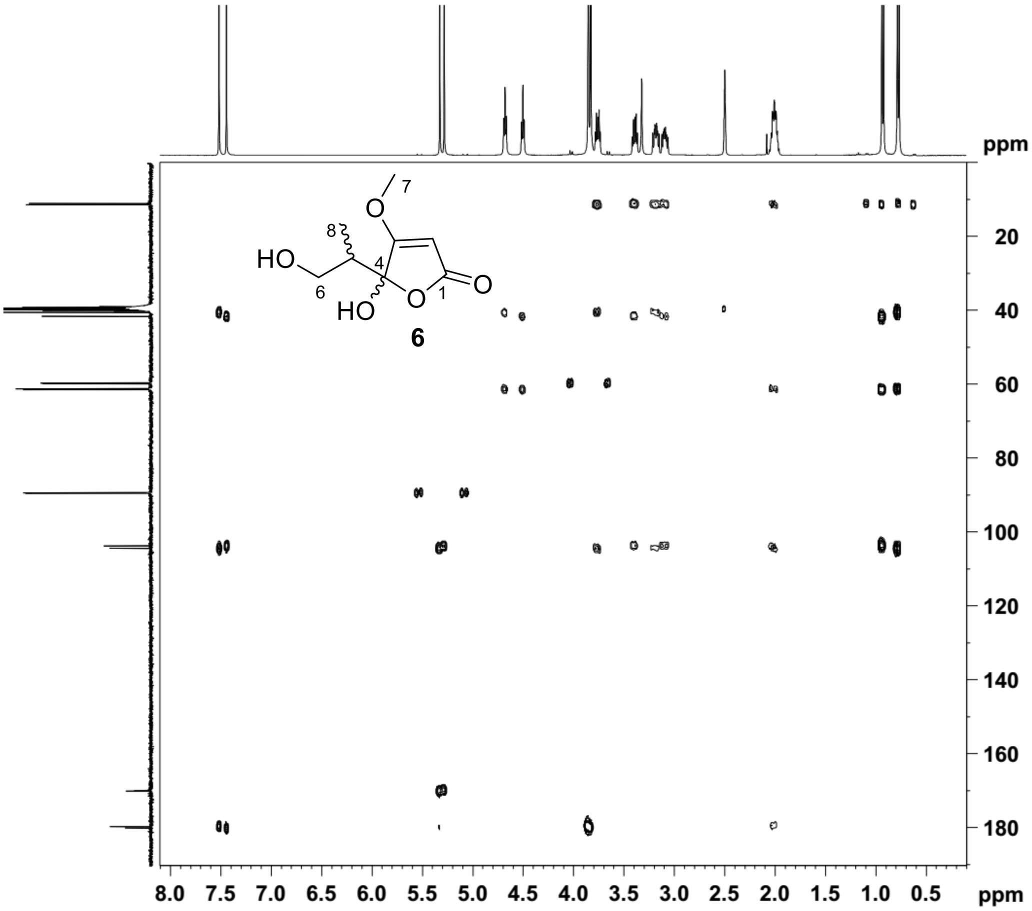

Figure S41. HMBC spectrum of **6** in DMSO-d_6.

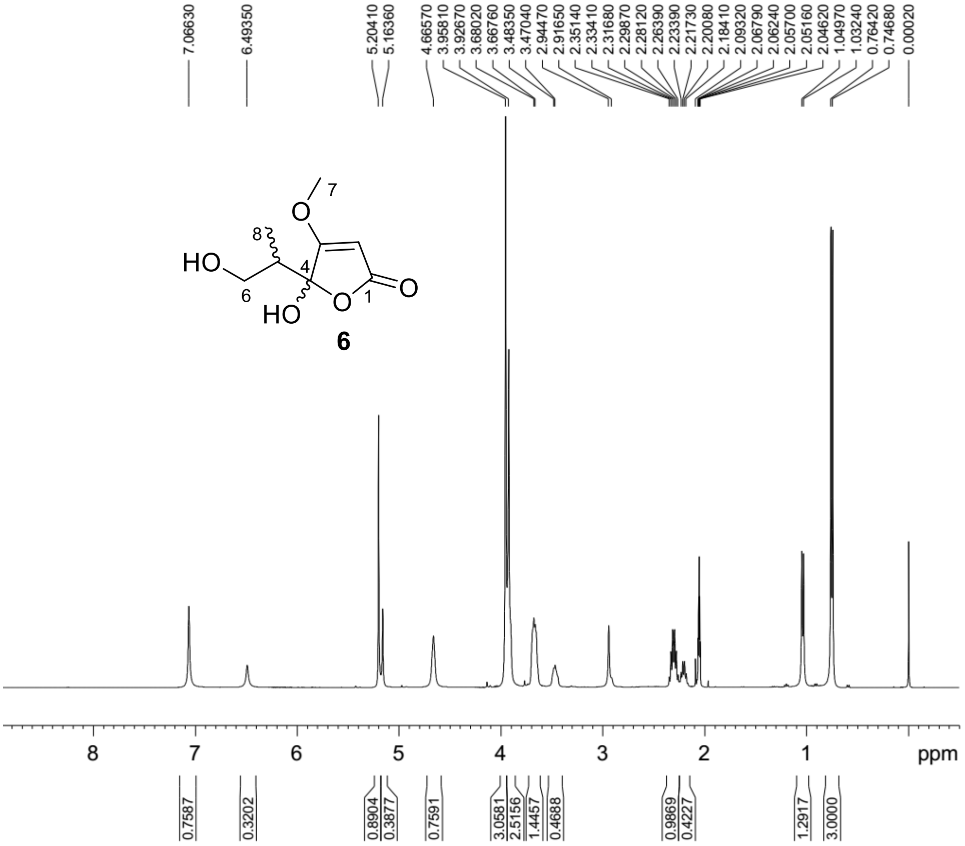

Figure S42. ^{1}H NMR spectrum (400 MHz) of **6** in acetone-d_6.

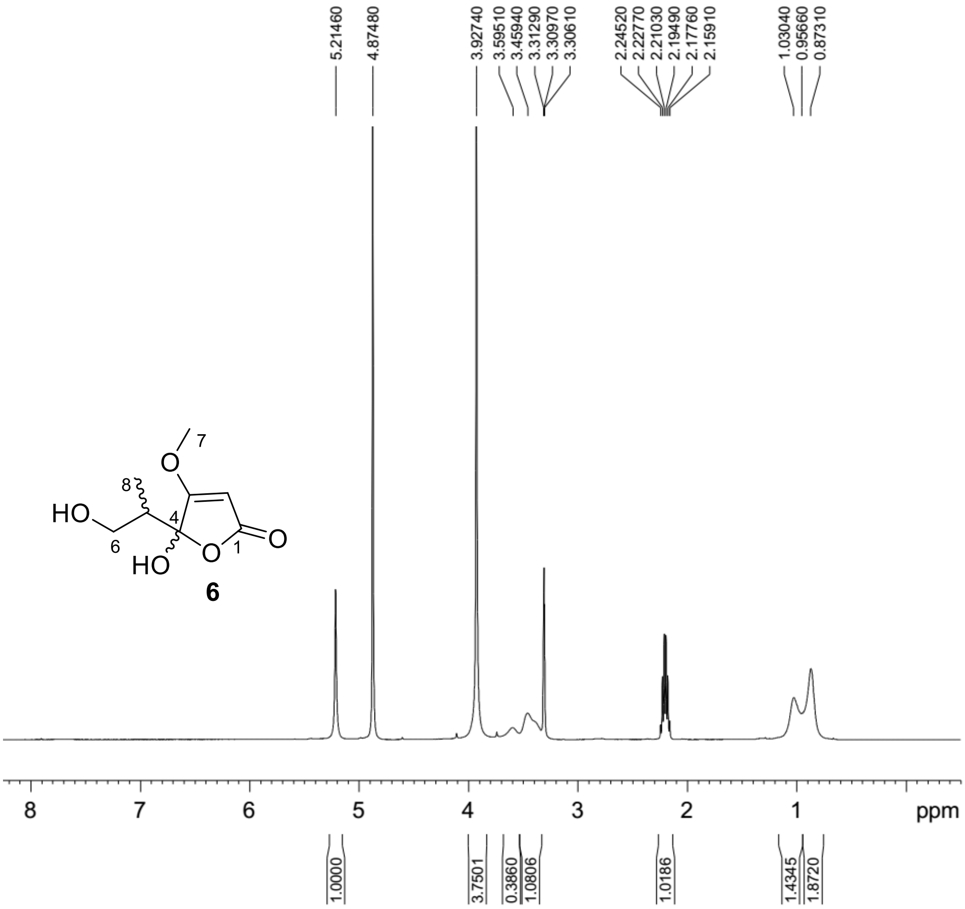

Figure S43. ^{1}H NMR spectrum (400 MHz) of **6** in MeOH-d_4.

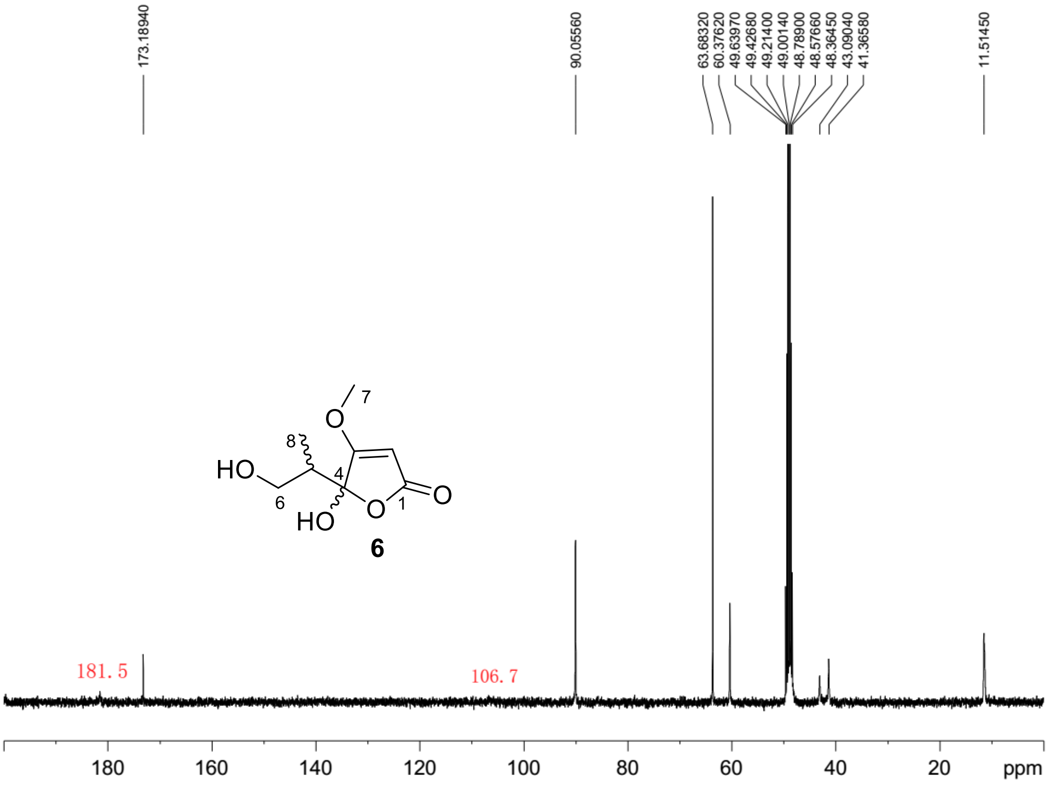

Figure S44. ^{13}C NMR spectrum (100 MHz) of **6** in MeOH-d_4.

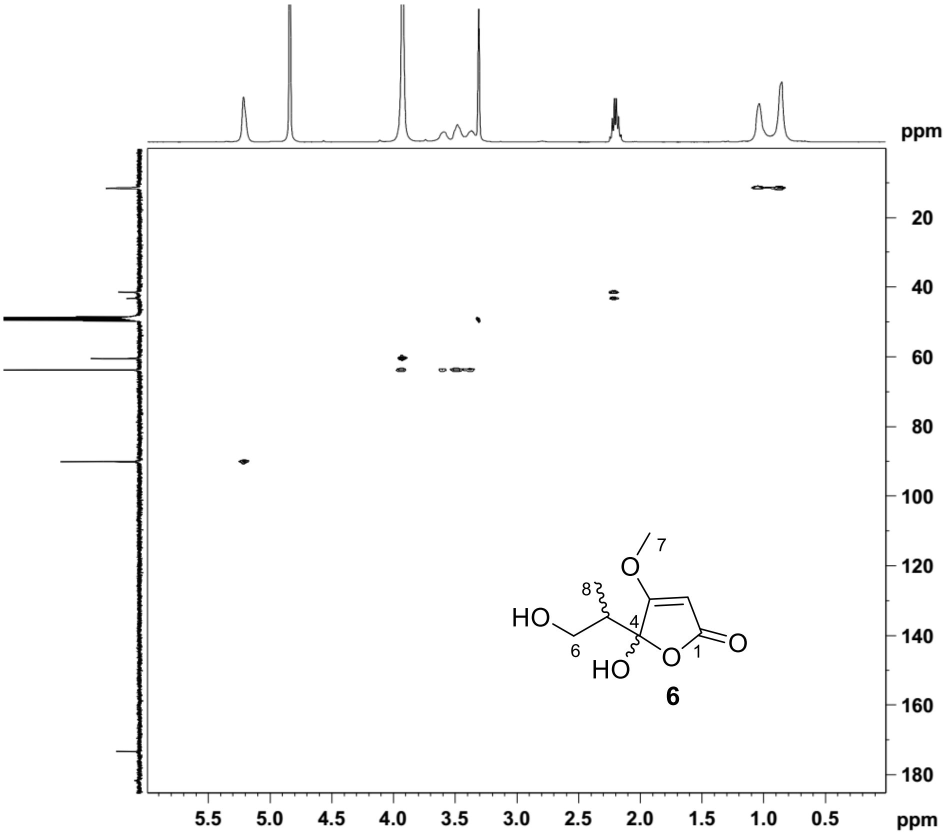

Figure S45. HSQC spectrum of **6** in MeOH-d_4.

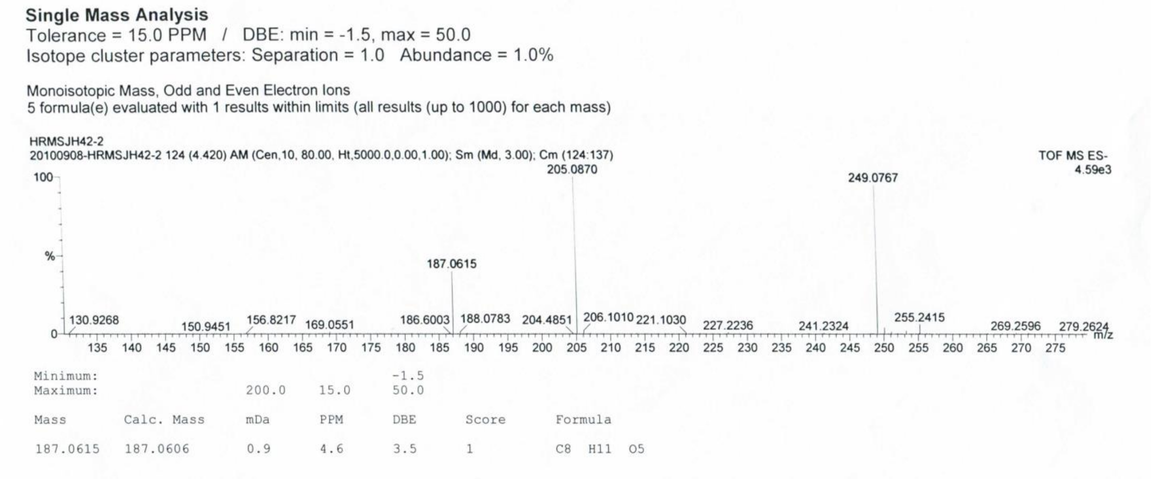

Figure S46. HRESIMS of **6**.

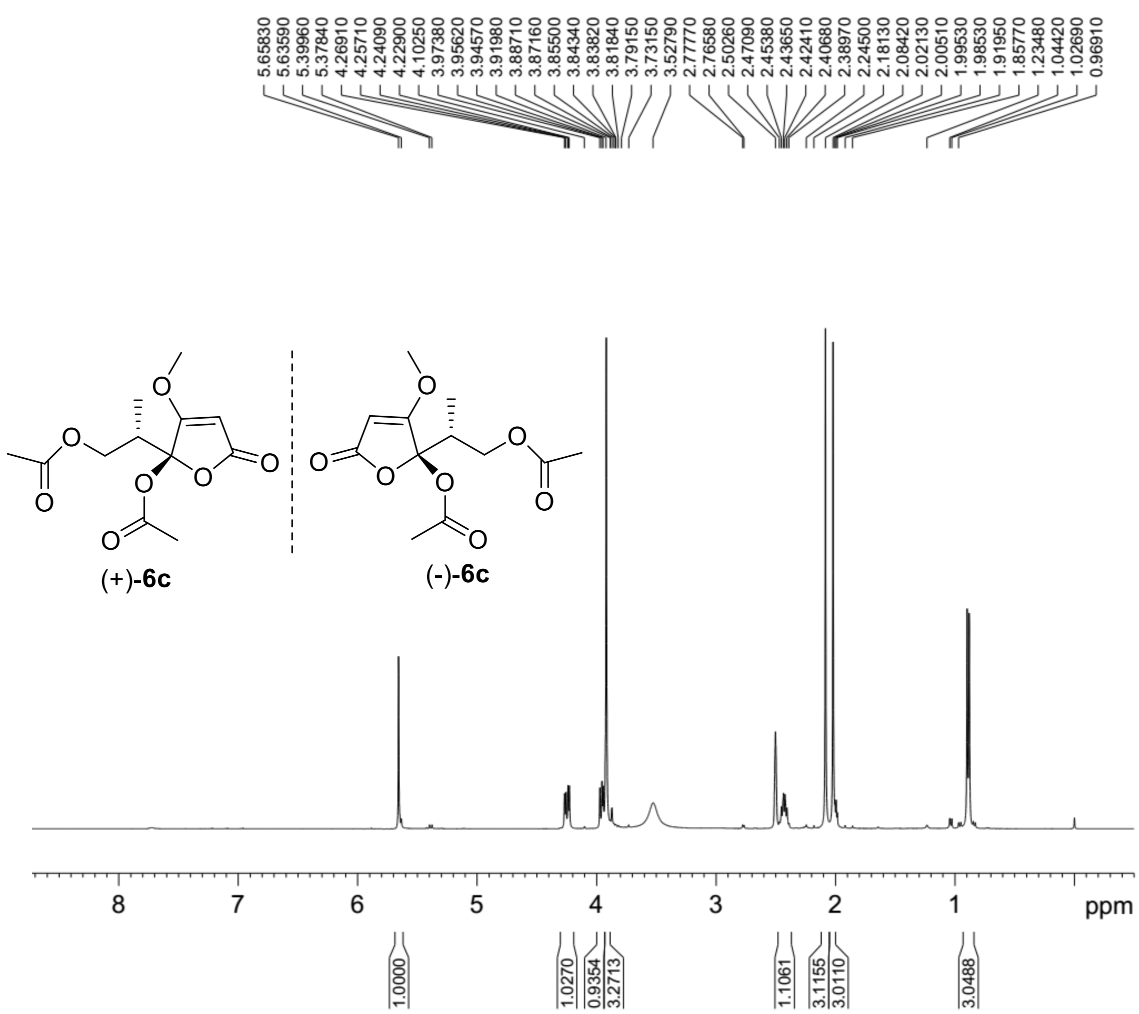

Figure S47. ^{1}H NMR spectrum (400 MHz) of **6c** in DMSO-d_6.

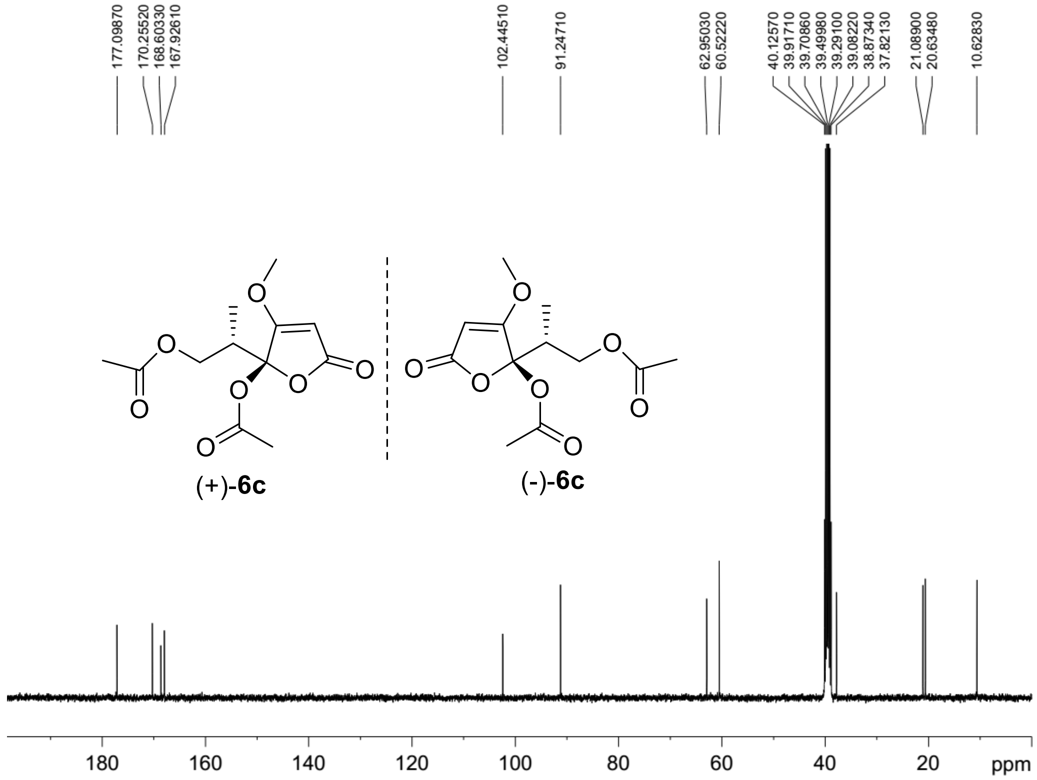

Figure S48. ^{13}C NMR spectrum (100 MHz) of **6c** in DMSO-d_6.

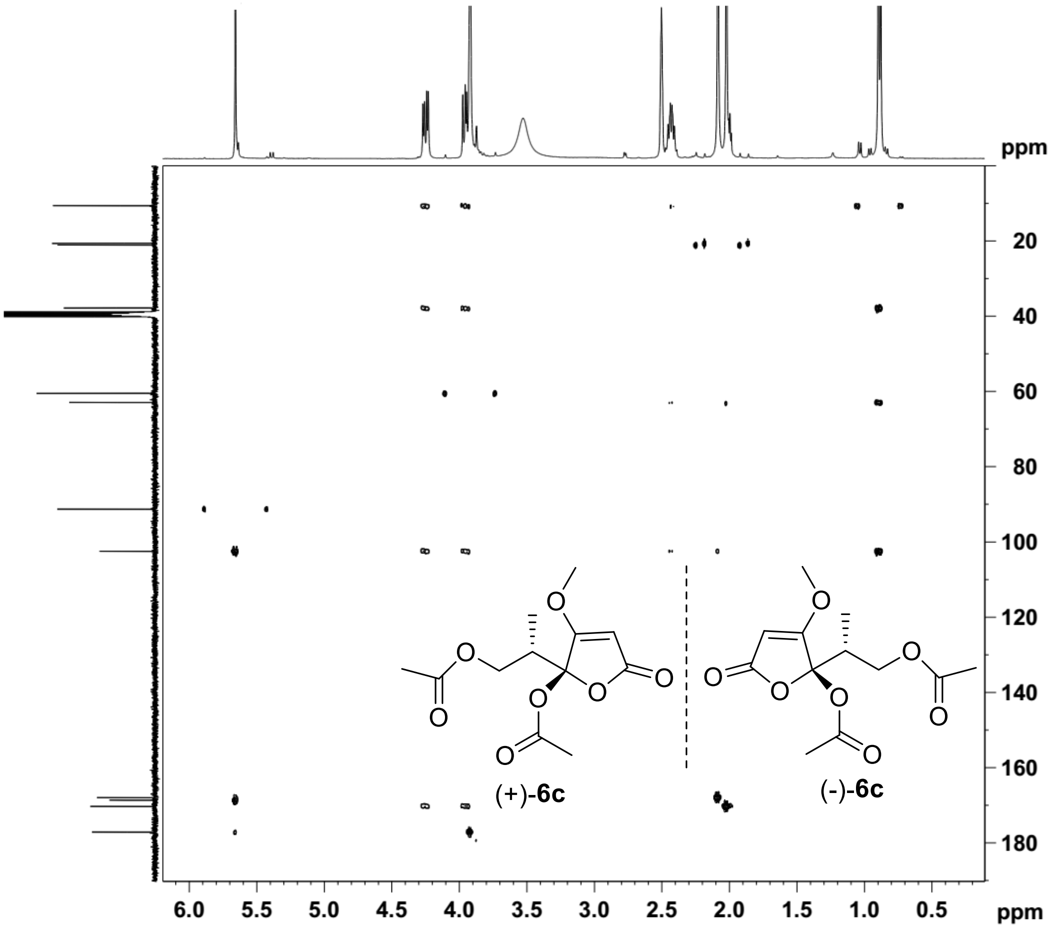

Figure S49. HMBC spectrum of **6c** in DMSO-d_6.

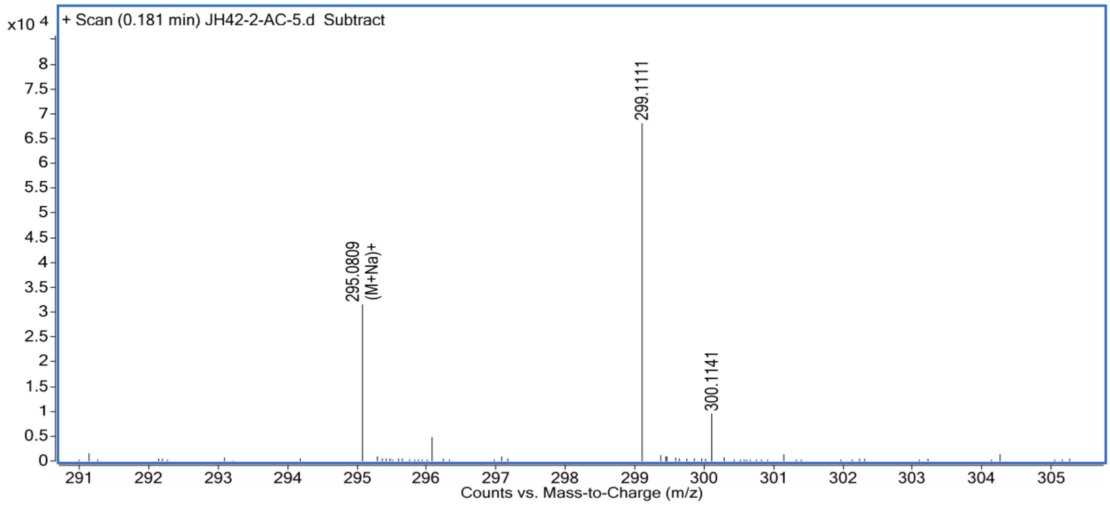

Name	Formula	Score	Mass	Mass (MFG)	Diff (ppm)	Diff (abs. ppm)	Diff (mDa)
	C12 H16 O7	85.48	272.0917	272.0896	-7.78	7.78	-2.12

Species	Ion Formula	m/z	Height	Score (MFG)	Score (MFG, MS)	Score (MFG, mass)	Score (MFG, abund)	Score (MFG, iso. spacing)
(M+Na)+	C12 H16 Na O7	295.0788	31516.6	85.48	85.48	71.45	97.74	98.84

m/z	m/z (Calc)	Diff (ppm)	Diff (mDa)	Height	Height (Calc)	Height %	Height % (Calc)	Height Sum %	Height Sum% (Calc)
295.0809	295.0788	-7.18	-2.1	31516.6	31986.9	100	100	85.2	86.4
296.0839	296.0822	-5.7	-1.7	4693.9	4295.7	14.9	13.4	12.7	11.6
297.0856	297.084	-5.47	-1.6	798.2	726.1	2.5	2.3	2.2	2

Figure S50. HRESIMS of **6c**.

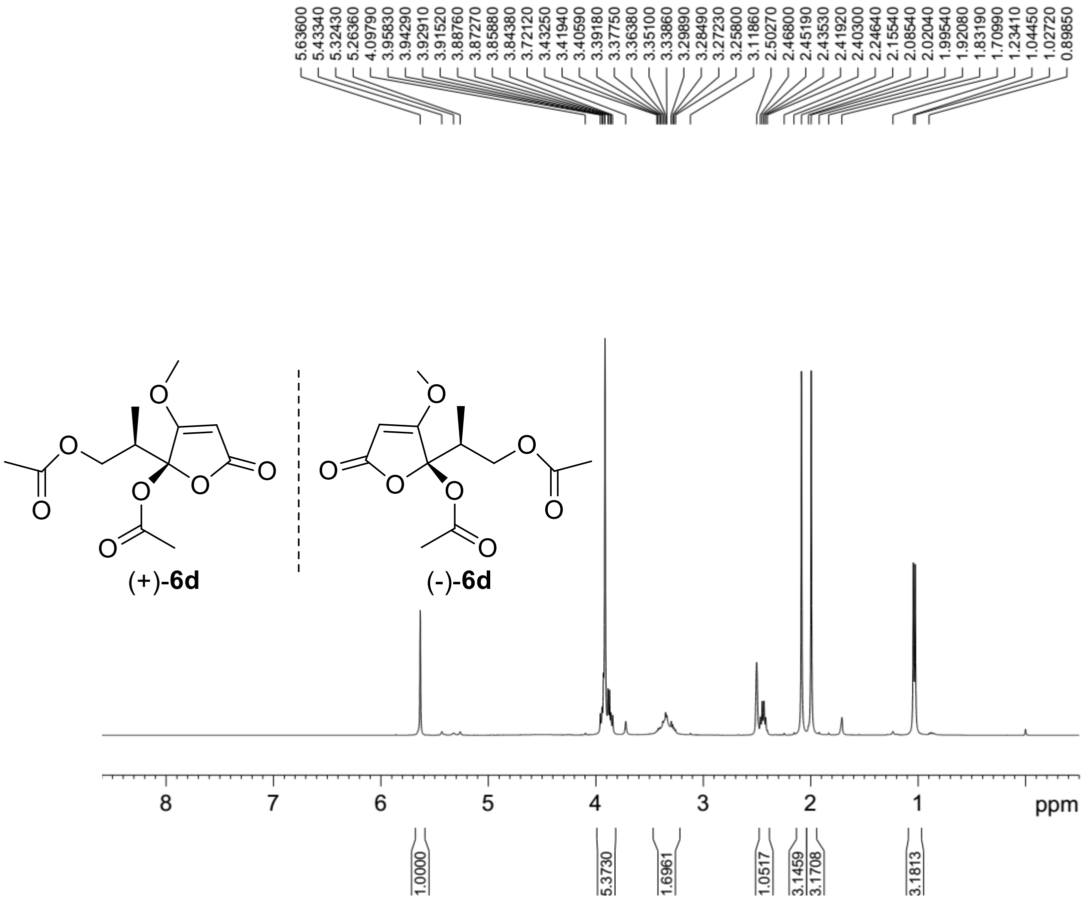

Figure S51. ^{1}H NMR spectrum (400 MHz) of **6d** in DMSO-d_6.

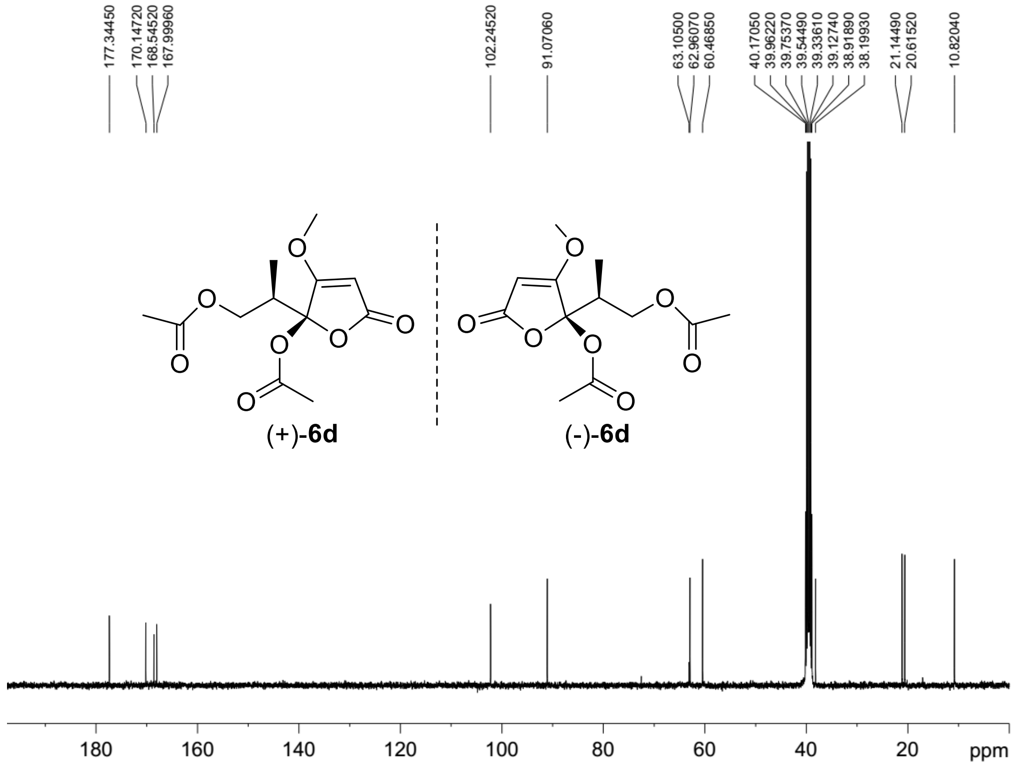

Figure S52. ^{13}C NMR spectrum (100 MHz) of **6d** in DMSO-d_6.

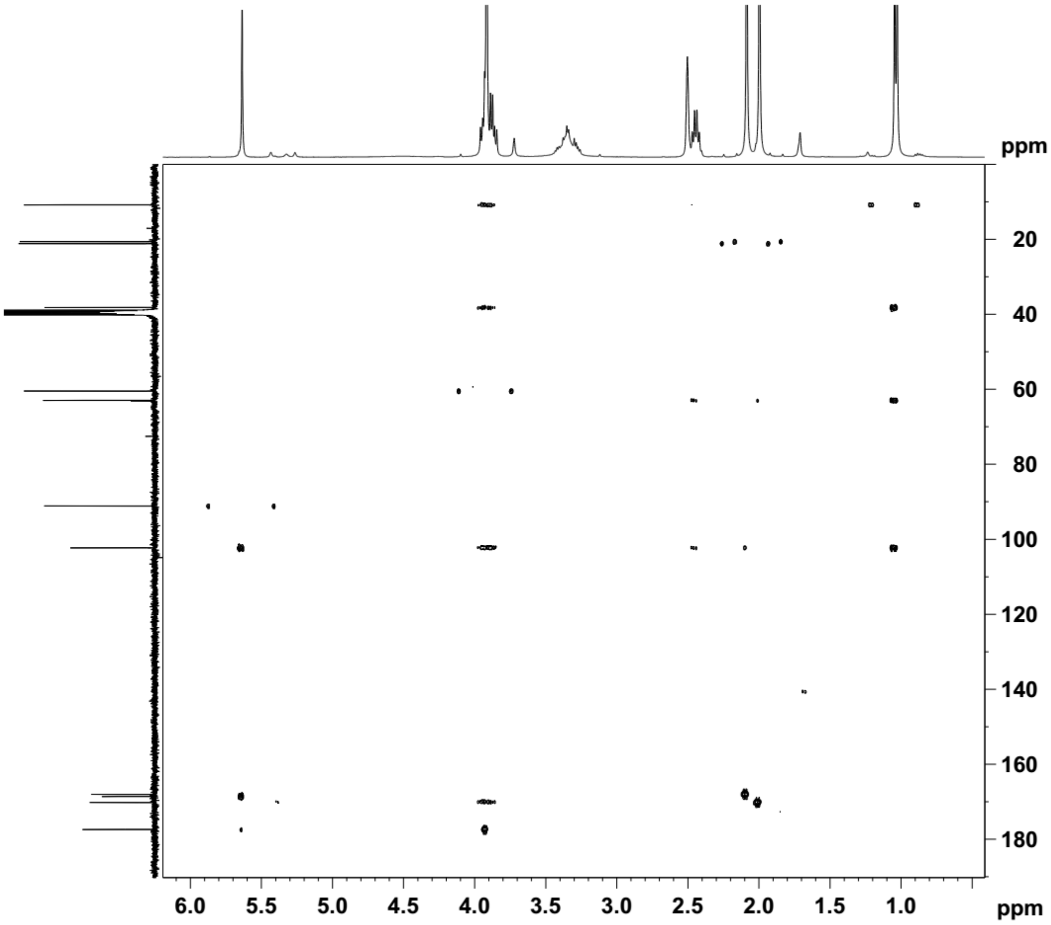

Figure S53. HMBC spectrum of **6d** in DMSO-d_6.

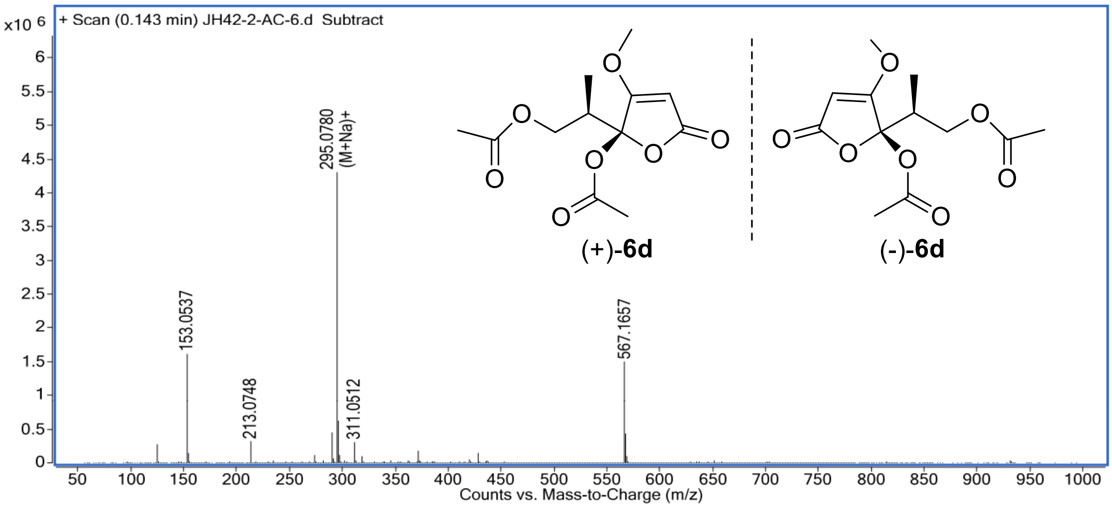

Name	Formula	Score	Mass	Mass (MFG)	Diff (ppm)	Diff (abs. ppm)	Diff (mDa)
	C12 H16 O7	97.5	272.0888	272.0896	2.89	2.89	0.79

Species	Ion Formula	m/z	Height	Score (MFG)	Score (MFG, MS)	Score (MFG, mass)	Score (MFG, abund)	Score (MFG, iso. spacing)
(M+Na)+	C12 H16 Na O7	295.0788	4303675	97.5	97.5	95.47	98.84	99.96

m/z	m/z (Calc)	Diff (ppm)	Diff (mDa)	Height	Height (Calc)	Height %	Height % (Calc)	Height Sum %	Height Sum% (Calc)
295.078	295.0788	2.67	0.8	4303675	4347022.9	100	100	85.6	86.4
296.0814	296.0822	2.78	0.8	621920.8	583785.3	14.5	13.4	12.4	11.6
297.0828	297.084	4.1	1.2	103890.3	98677.8	2.4	2.3	2.1	2

Figure S54. HRESIMS of **6d**.

Attachment S1. Supporting information for the calculated ECD spectra of compounds **4**, **5**, and **6c**.

1. Computational methods

1.1 Conformational analysis

Conformational analysis was initially performed using Confab [1] with systematic search at MMFF94 force field for undetermined relative configurations of compounds **4**, **5**, and **6c** (**Figure AS1**). Room-temperature equilibrium populations were calculated according to Boltzmann distribution law (1) and those with populations lower than 1% were filtered. The energies and populations of dominative conformers were provided in **Table AS1**.

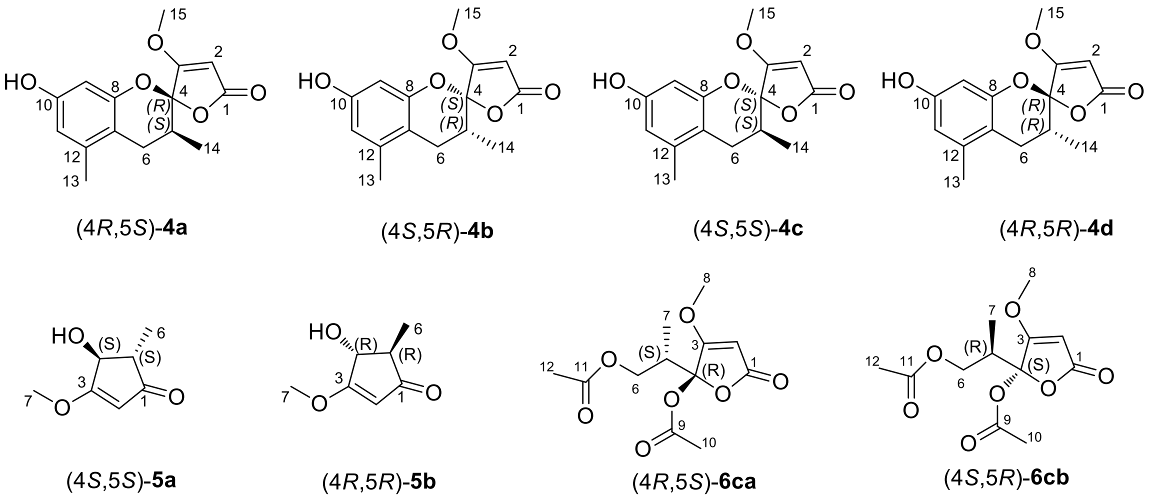

Figure AS1. Chemical structure of all undetermined relative configurations of compounds **4**, **5**, and **6c**.

$$\frac{N_i}{N} = \frac{g_i e^{-\frac{E_i}{k_B T}}}{\sum g_i e^{-\frac{E_i}{k_B T}}} \qquad \textbf{(1)}$$

Where N_i is the number of conformer i with energy E_i and degeneracy g_i at temperature *T*, and k_B is Boltzmann constant.

1.2 ECD calculation

The theoretical calculations were carried out using Gaussian 09 [2]. At first, conformers were optimized at PM6 using semi-empirical theory method and again filtered by Boltzmann-based populations. The remaining structures were finally optimized at B3LYP/6-311G(d,p) in methanol using the IEFPCM model (**Error! Reference source not found.**). Vibrational frequency analysis confirmed the stable structures. Based on the optimized structures, the ECD calculation was conducted using Time-dependent Density functional theory (TD-DFT) in methanol at B3LYP/6-311G(d,p) for compounds **5** and **6c**, and at BP86/6-311G(d,p) for compound **4**. Rotatory strengths for a total of 30 excited states were calculated. The ECD spectrum was simulated in SpecDis [3] by overlapping Gaussian functions for each transition according to (2).

$$\Delta\varepsilon(E) = \frac{1}{2.297\times10^{-39}} \times \frac{1}{\sqrt{2\pi\sigma}} \sum_i^A \Delta E_i R_i e^{-\left(\frac{E-E_i}{2\sigma}\right)^2} \qquad \textbf{(2)}$$

Where σ represents the width of the band at 1/*e* height, and ΔE_i and R_i are the excitation energies and rotatory strengths for transition *i*, respectively.

The σ values were 0.25 eV, 0.30 eV, and 0.32 eV for compounds **4**, **5**, and **6c** and the UV-shift values were set -5 nm, 27 nm, and 4

nm, respectively.

1.3 References

1. O'Boyle, N.M.; Vandermeersch, T.; Flynn, C.J.; Maguire, A.R.; Hutchison, G.R. Confab-systematic generation of diverse low-energy conformers. *J. Cheminformatics*, **2011**, *3 (3)*, 8–16.
2. Frisch, M.J.; Trucks, G.W.; Schlegel, H.B.; Scuseria, G.E.; Robb, M.A.; Cheeseman, J.R.; Scalmani, G.; Barone, V.; Mennucci, B.; Petersson, G.A.; Nakatsuji, H.; Caricato, M.; Li, X.; Hratchian, H.P.; Izmaylov, A.F.; Bloino, J.; Zheng, G.; Sonnenberg, J.L.; Hada, M.; Ehara, M.; Toyota, K.; Fukuda, R.; Hasegawa, J.; Ishida, M.; Nakajima, T.; Honda, Y.; Kitao, O.; Nakai, H.; Vreven, T.; Montgomery Jr., J.A.; Peralta, J.E.; Ogliaro, F.; Bearpark, M.; Heyd, J.J.; Brothers, E.; Kudin, K.N.; Staroverov, V.N.; Kobayashi, R.; Normand, J.; Raghavachari, K.; Rendell, A.; Burant, J.C.; Iyengar, S.S.; Tomasi, J.; Cossi, M.; Rega, N.; Millam, J.M.; Klene, M.; Knox, J.E.; Cross, J.B.; Bakken, V.; Adamo, C.; Jaramillo, J.; Gomperts, R.; Stratmann, R.E.; Yazyev, O.; Austin, A.J.; Cammi, R.; Pomelli, C.; Ochterski, J.W.; Martin, R.L.; Morokuma, K.; Zakrzewski, V.G.; Voth, G.A.; Salvador, P.; Dannenberg, J.J.; Dapprich, S.; Daniels, A.D.; Farkas, O.; Foresman, J.B.; Ortiz, J.V.; Cioslowski, J.; Fox, D.J. Gaussian 09 Revision D.01. Gaussian Inc., Wallingford CT 2009.
3. Bruhn, T.; Schaumlöffel, A.; Hemberger, Y.; Bringmann, G. Specdis: quantifying the comparison of calculated and experimental electronic circular dichroism spectra. *Chirality* **2013**, *25(4)*, 243–249.
4. Lodewyk, M.W.; Siebert M.R.; Tantillo, D.J. Computational prediction of ^{1}H and ^{13}C chemical shifts: a useful tool for natural product, mechanistic, and synthetic organic chemistry. *Chem. Rev.* **2012**, *112* (3), 1839–1862.

2. Energies and Coordinates

2.1 Energies at MMFF94 force field

Systematic conformational search was performed by Confab program at MMFF94 force field. Conformers for each configuration were obtained with filtration by RMSD threshold of 0.5 Å.

Table AS1 Energies of compounds **4**, **5**, and **6c** at MMFF94 force field.

Configuration	Conformer	Energy (kcal/mol)	Population (%)
**4a**	1	4.64	85.29
**4a**	2	5.69	14.71
**4b**	1	4.65	85.27
**4b**	2	5.69	14.73
**4c**	1	5.82	20.41
**4c**	2	5.02	79.59
**4d**	1	5.82	20.43
**4d**	2	5.02	79.57
**5a**	1	7.11	99.78
**5b**	1	7.38	99.65
**6ca**	1	-39.54	25.50
**6ca**	2	-39.40	19.89
**6ca**	3	-38.76	6.77
**6ca**	4	-38.64	5.50
**6ca**	5	-38.57	4.96
**6ca**	6	-38.54	4.67
**6ca**	7	-38.36	3.48
**6ca**	8	-38.35	3.38
**6ca**	9	-38.07	2.14
**6ca**	10	-38.00	1.89
**6ca**	11	-37.88	1.55
**6ca**	12	-37.87	1.52
**6ca**	13	-37.85	1.45
**6ca**	14	-37.78	1.30
**6ca**	15	-37.70	1.13
**6ca**	16	-37.68	1.11
**6ca**	17	-37.62	1.00
**6cb**	1	-39.51	19.18
**6cb**	2	-39.42	16.57
**6cb**	3	-39.40	16.04
**6cb**	4	-38.94	7.32
**6cb**	5	-38.84	6.25
**6cb**	6	-38.54	3.77
**6cb**	7	-38.42	3.09
**6cb**	8	-38.27	2.38
**6cb**	9	-38.26	2.33
**6cb**	10	-38.23	2.22
**6cb**	11	-38.10	1.80

**6cb**	12	-38.10	1.79
**6cb**	13	-37.89	1.24
**6cb**	14	-37.81	1.09
**6cb**	15	-37.80	1.07
**6cb**	16	-37.78	1.04

2.2 Energies at B3LYP theory level

Structures for ECD calculations were optimized at B3LYP/6-311G(d,p) in methanol.

Table AS2 Energies of compounds **4**, **5**, and **6c** at B3LYP/6-311G(d,p) in methanol.

Configuration	Conformer	Structure	E (Hartree)	E (kcal/mol)	Population (%)
**4a**	1	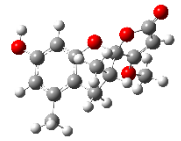	-957.58341493	-600892.66	100
**4b**	1	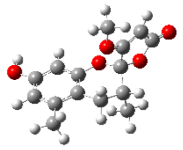	-957.58341493	-600892.66	100
**4c**	1	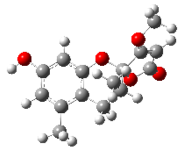	-957.58521469	-600893.79	100
**4d**	1	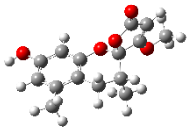	-957.58521469	-600893.79	100
**5a**	1	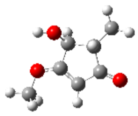	-498.54159321	-312839.57	100.00
**5b**	1	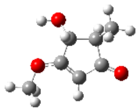	-498.54159322	-312839.57	100.00
**6ca**	1	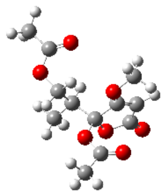	-993.76209792	-623595.13	34.12

**6ca**	2	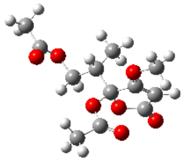	-993.76271899	-623595.52	65.88
**6cb**	1	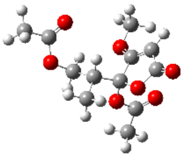	-993.76209795	-623595.13	26.77
**6cb**	2	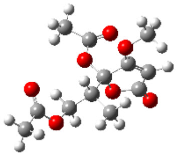	-993.76271900	-623595.52	51.68
**6cb**	4	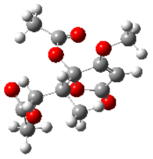	-993.76189351	-623595.00	21.56

2.3 Coordinates at B3LYP theory level

Table AS3 Standard orientations of compounds **4**, **5**, and **6c** at B3LYP/6-311G(d,p) level in methanol.

Conformer **4a**-1

Center	Atomic	Atomic	Coordinates (Angstroms)		
Number	Number	Type	X	Y	Z
1	6	0	-3.198983	-1.382865	-0.485884
2	6	0	-3.663771	-0.204543	0.102471
3	1	0	-4.719843	-0.118868	0.327582
4	6	0	-2.784830	0.835834	0.389039
5	6	0	-1.413206	0.711997	0.088582
6	6	0	-0.974777	-0.482505	-0.485908
7	6	0	-1.849646	-1.529082	-0.779880
8	1	0	-1.452390	-2.431448	-1.231466
9	6	0	-0.412981	1.804994	0.384467
10	1	0	-0.847524	2.786885	0.177235
11	1	0	-0.157715	1.798126	1.449312
12	6	0	0.860096	1.653360	-0.467591
13	1	0	1.658856	2.262785	-0.032818
14	6	0	1.349237	0.196077	-0.419546
15	8	0	0.339036	-0.720134	-0.828739
16	6	0	-3.313284	2.100190	1.024158
17	1	0	-4.379299	2.011810	1.237656
18	1	0	-3.175301	2.967063	0.369234
19	1	0	-2.799029	2.325046	1.963928
20	8	0	-4.117915	-2.360834	-0.744581
21	1	0	-3.675777	-3.107007	-1.162251
22	6	0	0.632725	2.107836	-1.917807
23	1	0	1.531992	1.970813	-2.517219
24	1	0	0.363482	3.167141	-1.929187
25	1	0	-0.179164	1.548539	-2.387520
26	8	0	2.419544	0.013917	-1.329078
27	6	0	3.533670	-0.524353	-0.688892
28	6	0	3.204189	-0.694855	0.729624
29	1	0	3.902617	-1.111870	1.436243
30	6	0	1.943386	-0.274424	0.909414
31	8	0	4.542948	-0.756550	-1.285403
32	8	0	1.160945	-0.241495	1.981948
33	6	0	1.704522	-0.784525	3.193207
34	1	0	2.585507	-0.216059	3.503501
35	1	0	1.969462	-1.835179	3.049638
36	1	0	0.919585	-0.695817	3.940223

Conformer **4b**-1

Center	Atomic	Atomic	Coordinates (Angstroms)		
Number	Number	Type	X	Y	Z
1	6	0	3.198983	-1.382865	-0.485884
2	6	0	3.663771	-0.204543	0.102471
3	1	0	4.719843	-0.118868	0.327582

4	6	0	2.784830	0.835834	0.389039
5	6	0	1.413206	0.711997	0.088582
6	6	0	0.974777	-0.482505	-0.485908
7	6	0	1.849646	-1.529082	-0.779880
8	1	0	1.452390	-2.431448	-1.231466
9	6	0	0.412981	1.804994	0.384467
10	1	0	0.847524	2.786885	0.177235
11	1	0	0.157715	1.798126	1.449312
12	6	0	-0.860096	1.653360	-0.467591
13	1	0	-1.658856	2.262785	-0.032818
14	6	0	-1.349237	0.196077	-0.419546
15	8	0	-0.339036	-0.720134	-0.828739
16	6	0	3.313284	2.100190	1.024158
17	1	0	4.379299	2.011810	1.237656
18	1	0	3.175301	2.967063	0.369234
19	1	0	2.799029	2.325046	1.963928
20	8	0	4.117915	-2.360834	-0.744581
21	1	0	3.675777	-3.107007	-1.162251
22	6	0	-0.632725	2.107836	-1.917807
23	1	0	-1.531992	1.970813	-2.517219
24	1	0	-0.363482	3.167141	-1.929187
25	1	0	0.179164	1.548539	-2.387520
26	8	0	-2.419544	0.013917	-1.329078
27	6	0	-3.533670	-0.524353	-0.688892
28	6	0	-3.204189	-0.694855	0.729624
29	1	0	-3.902617	-1.111870	1.436243
30	6	0	-1.943386	-0.274424	0.909414
31	8	0	-4.542948	-0.756550	-1.285403
32	8	0	-1.160945	-0.241495	1.981948
33	6	0	-1.704522	-0.784525	3.193207
34	1	0	-2.585507	-0.216059	3.503501
35	1	0	-1.969462	-1.835179	3.049638
36	1	0	-0.919585	-0.695817	3.940223

Conformer **4c**-1

Center Number	Atomic Number	Atomic Type	Coordinates (Angstroms)		
			X	Y	Z
1	6	0	3.265586	1.443720	0.484805
2	6	0	3.800685	0.193387	0.166987
3	1	0	4.876182	0.043619	0.201840
4	6	0	2.969017	-0.867682	-0.191647
5	6	0	1.575815	-0.684968	-0.240563
6	6	0	1.070829	0.574152	0.100748
7	6	0	1.891031	1.639097	0.455564
8	1	0	1.459846	2.597073	0.712844
9	6	0	0.620543	-1.795420	-0.616880
10	1	0	1.039980	-2.406928	-1.420446
11	1	0	0.464001	-2.458847	0.239795
12	6	0	-0.737179	-1.234827	-1.082395
13	1	0	-1.474711	-2.042606	-1.069942

14	6	0	-1.184319	-0.215142	-0.018556
15	8	0	-0.282784	0.861072	0.086871
16	6	0	3.577168	-2.210311	-0.520979
17	1	0	3.175715	-3.000657	0.120733
18	1	0	4.660210	-2.191179	-0.390138
19	1	0	3.372475	-2.503651	-1.556054
20	8	0	4.046335	2.508617	0.837802
21	1	0	4.968779	2.233863	0.839019
22	6	0	-0.656339	-0.646350	-2.497873
23	1	0	0.065226	0.171356	-2.550582
24	1	0	-1.619195	-0.265201	-2.836648
25	1	0	-0.333526	-1.424986	-3.194087
26	8	0	-1.268948	-0.905844	1.236987
27	6	0	-2.527657	-0.747376	1.800468
28	6	0	-3.335115	0.070853	0.888363
29	1	0	-4.353681	0.342235	1.111333
30	6	0	-2.571989	0.397593	-0.165128
31	8	0	-2.814199	-1.243721	2.850455
32	8	0	-2.809504	1.148661	-1.233828
33	6	0	-4.097508	1.774553	-1.302458
34	1	0	-4.888395	1.019858	-1.332901
35	1	0	-4.099731	2.355202	-2.221694
36	1	0	-4.243783	2.432901	-0.442117

Conformer **4d**-1

Center Number	Atomic Number	Atomic Type	Coordinates (Angstroms)		
			X	Y	Z
1	6	0	-3.265586	1.443720	0.484805
2	6	0	-3.800685	0.193387	0.166987
3	1	0	-4.876182	0.043619	0.201840
4	6	0	-2.969017	-0.867682	-0.191647
5	6	0	-1.575815	-0.684968	-0.240563
6	6	0	-1.070829	0.574152	0.100748
7	6	0	-1.891031	1.639097	0.455564
8	1	0	-1.459846	2.597073	0.712844
9	6	0	-0.620543	-1.795420	-0.616880
10	1	0	-1.039980	-2.406928	-1.420446
11	1	0	-0.464001	-2.458847	0.239795
12	6	0	0.737179	-1.234827	-1.082395
13	1	0	1.474711	-2.042606	-1.069942
14	6	0	1.184319	-0.215142	-0.018556
15	8	0	0.282784	0.861072	0.086871
16	6	0	-3.577168	-2.210311	-0.520979
17	1	0	-3.175715	-3.000657	0.120733
18	1	0	-4.660210	-2.191179	-0.390138
19	1	0	-3.372475	-2.503651	-1.556054
20	8	0	-4.046335	2.508617	0.837802
21	1	0	-4.968779	2.233863	0.839019
22	6	0	0.656339	-0.646350	-2.497873
23	1	0	-0.065226	0.171356	-2.550582

24	1	0	1.619195	-0.265201	-2.836648
25	1	0	0.333526	-1.424986	-3.194087
26	8	0	1.268948	-0.905844	1.236987
27	6	0	2.527657	-0.747376	1.800468
28	6	0	3.335115	0.070853	0.888363
29	1	0	4.353681	0.342235	1.111333
30	6	0	2.571989	0.397593	-0.165128
31	8	0	2.814199	-1.243721	2.850455
32	8	0	2.809504	1.148661	-1.233828
33	6	0	4.097508	1.774553	-1.302458
34	1	0	4.888395	1.019858	-1.332901
35	1	0	4.099731	2.355202	-2.221694
36	1	0	4.243783	2.432901	-0.442117

Conformer **5a**-1

Center	Atomic	Atomic	Coordinates (Angstroms)		
Number	Number	Type	X	Y	Z
1	6	0	-0.123080	1.061651	0.195321
2	1	0	-0.170661	1.417757	1.236010
3	6	0	0.907717	-0.044764	0.107297
4	6	0	0.371808	-1.266847	-0.086409
5	1	0	0.878289	-2.221017	-0.142120
6	6	0	-1.086779	-1.135183	-0.200705
7	6	0	-1.430270	0.369498	-0.210449
8	1	0	-1.601888	0.639034	-1.259523
9	8	0	0.161894	2.148097	-0.676110
10	1	0	1.020766	2.511057	-0.428161
11	6	0	-2.666882	0.715202	0.612871
12	1	0	-2.510747	0.504351	1.675250
13	1	0	-3.515866	0.115765	0.280171
14	1	0	-2.925663	1.771782	0.509290
15	8	0	2.176404	0.347055	0.252256
16	8	0	-1.899951	-2.031033	-0.278492
17	6	0	3.188723	-0.659095	0.145846
18	1	0	4.139550	-0.143944	0.260043
19	1	0	3.141031	-1.145990	-0.831360
20	1	0	3.070991	-1.404524	0.936539

Conformer **5b**-1

Center	Atomic	Atomic	Coordinates (Angstroms)		
Number	Number	Type	X	Y	Z
1	6	0	0.123080	1.061651	0.195321
2	1	0	0.170661	1.417757	1.236010
3	6	0	-0.907717	-0.044764	0.107297
4	6	0	-0.371808	-1.266847	-0.086409
5	1	0	-0.878289	-2.221017	-0.142120
6	6	0	1.086779	-1.135183	-0.200705
7	6	0	1.430270	0.369498	-0.210449
8	1	0	1.601888	0.639034	-1.259523
9	8	0	-0.161894	2.148097	-0.676110
10	1	0	-1.020766	2.511057	-0.428161

11	6	0	2.666882	0.715202	0.612871
12	1	0	2.510747	0.504351	1.675250
13	1	0	3.515866	0.115765	0.280171
14	1	0	2.925663	1.771782	0.509290
15	8	0	-2.176404	0.347055	0.252256
16	8	0	1.899951	-2.031033	-0.278492
17	6	0	-3.188723	-0.659095	0.145846
18	1	0	-4.139550	-0.143944	0.260043
19	1	0	-3.141031	-1.145990	-0.831360
20	1	0	-3.070991	-1.404524	0.936539

Conformer **6ca**-1

Center	Atomic	Atomic	Coordinates (Angstroms)		
Number	Number	Type	X	Y	Z
1	6	0	0.833214	-1.086099	-0.381497
2	6	0	0.709146	0.217730	0.405306
3	8	0	1.463815	-0.036637	1.576316
4	6	0	1.878935	-1.376243	1.609282
5	6	0	1.429879	-2.018317	0.375250
6	1	0	1.651841	-3.049073	0.149936
7	8	0	2.487407	-1.807677	2.541981
8	6	0	-0.744406	0.571464	0.805361
9	1	0	-1.167553	-0.359567	1.196112
10	6	0	-1.585316	0.988766	-0.409765
11	1	0	-1.460404	0.303833	-1.243325
12	1	0	-1.338261	2.001387	-0.724466
13	8	0	-2.987957	1.043146	-0.056257
14	8	0	0.332699	-1.112406	-1.608969
15	6	0	-0.783587	1.640714	1.903886
16	1	0	-0.312569	2.566795	1.566120
17	1	0	-0.266737	1.303797	2.801598
18	1	0	-1.820459	1.863392	2.162283
19	8	0	1.218165	1.376228	-0.262884
20	6	0	-3.690837	-0.107250	-0.185117
21	6	0	-5.127329	0.096919	0.226515
22	1	0	-5.710335	-0.783591	-0.035460
23	1	0	-5.543981	0.984037	-0.253511
24	1	0	-5.181757	0.256206	1.306770
25	6	0	2.463374	1.362274	-0.837070
26	6	0	2.812556	2.742899	-1.327407
27	1	0	2.017902	3.138958	-1.962692
28	1	0	3.749780	2.704897	-1.878446
29	1	0	2.917087	3.419204	-0.475358
30	8	0	-3.215139	-1.146396	-0.569461
31	8	0	3.152684	0.386568	-0.923829
32	6	0	0.405378	-2.364048	-2.305127
33	1	0	-0.168638	-3.123872	-1.770476
34	1	0	1.447497	-2.674884	-2.408209
35	1	0	-0.032853	-2.186993	-3.283741

Conformer **6ca**-2

Center Number	Atomic Number	Atomic Type	Coordinates (Angstroms) X	Y	Z
1	6	0	-1.665154	-0.569139	0.758272
2	6	0	-0.578399	-0.096139	-0.196126
3	8	0	-1.015285	-0.568203	-1.458596
4	6	0	-2.188538	-1.326407	-1.313633
5	6	0	-2.550625	-1.333367	0.104505
6	1	0	-3.436531	-1.829714	0.467149
7	8	0	-2.708283	-1.841112	-2.257326
8	6	0	0.825487	-0.649419	0.140766
9	1	0	1.111829	-0.171012	1.082077
10	6	0	1.826314	-0.203923	-0.935863
11	1	0	1.817788	0.876740	-1.049118
12	1	0	1.607812	-0.681336	-1.889936
13	8	0	3.167067	-0.631168	-0.602484
14	8	0	-1.545244	-0.198546	2.026030
15	6	0	0.830669	-2.171634	0.312312
16	1	0	0.451497	-2.675366	-0.580312
17	1	0	0.228076	-2.489427	1.165252
18	1	0	1.851183	-2.517933	0.479043
19	8	0	-0.409852	1.324676	-0.238275
20	6	0	3.907255	0.207039	0.163456
21	6	0	5.282640	-0.366942	0.399111
22	1	0	5.898643	0.364990	0.917325
23	1	0	5.747443	-0.647377	-0.547978
24	1	0	5.209535	-1.273832	1.004941
25	6	0	-1.479932	2.173626	-0.333609
26	6	0	-0.987534	3.586505	-0.504810
27	1	0	-0.538158	3.697692	-1.495152
28	1	0	-0.215074	3.818091	0.230279
29	1	0	-1.823953	4.275820	-0.409633
30	8	0	3.505995	1.258844	0.591730
31	8	0	-2.625974	1.825266	-0.286227
32	6	0	-2.603643	-0.593244	2.908636
33	1	0	-2.670779	-1.683064	2.959994
34	1	0	-3.552065	-0.174617	2.564844
35	1	0	-2.345873	-0.189438	3.884310

Conformer **6cb**-1

Center Number	Atomic Number	Atomic Type	Coordinates (Angstroms) X	Y	Z
1	6	0	-0.833214	-1.086099	-0.381497
2	6	0	-0.709146	0.217730	0.405306
3	8	0	-1.463815	-0.036637	1.576316
4	6	0	-1.878935	-1.376243	1.609282
5	6	0	-1.429879	-2.018317	0.375250
6	1	0	-1.651841	-3.049073	0.149936
7	8	0	-2.487407	-1.807677	2.541981
8	6	0	0.744406	0.571464	0.805361
9	1	0	1.167553	-0.359567	1.196112

10	6	0	1.585316	0.988766	-0.409765
11	1	0	1.460404	0.303833	-1.243325
12	1	0	1.338261	2.001387	-0.724466
13	8	0	2.987957	1.043146	-0.056257
14	8	0	-0.332699	-1.112406	-1.608969
15	6	0	0.783587	1.640714	1.903886
16	1	0	0.312569	2.566795	1.566120
17	1	0	0.266737	1.303797	2.801598
18	1	0	1.820459	1.863392	2.162283
19	8	0	-1.218165	1.376228	-0.262884
20	6	0	3.690837	-0.107250	-0.185117
21	6	0	5.127329	0.096919	0.226515
22	1	0	5.710335	-0.783591	-0.035460
23	1	0	5.543981	0.984037	-0.253511
24	1	0	5.181757	0.256206	1.306770
25	6	0	-2.463374	1.362274	-0.837070
26	6	0	-2.812556	2.742899	-1.327407
27	1	0	-2.017902	3.138958	-1.962692
28	1	0	-3.749780	2.704897	-1.878446
29	1	0	-2.917087	3.419204	-0.475358
30	8	0	3.215139	-1.146396	-0.569461
31	8	0	-3.152684	0.386568	-0.923829
32	6	0	-0.405378	-2.364048	-2.305127
33	1	0	0.168638	-3.123872	-1.770476
34	1	0	-1.447497	-2.674884	-2.408209
35	1	0	0.032853	-2.186993	-3.283741

Conformer **6cb**-2

Center	Atomic	Atomic	Coordinates (Angstroms)		
Number	Number	Type	X	Y	Z
1	6	0	1.665154	-0.569139	0.758272
2	6	0	0.578399	-0.096139	-0.196126
3	8	0	1.015285	-0.568203	-1.458596
4	6	0	2.188538	-1.326407	-1.313633
5	6	0	2.550625	-1.333367	0.104505
6	1	0	3.436531	-1.829714	0.467149
7	8	0	2.708283	-1.841112	-2.257326
8	6	0	-0.825487	-0.649419	0.140766
9	1	0	-1.111829	-0.171012	1.082077
10	6	0	-1.826314	-0.203923	-0.935863
11	1	0	-1.817788	0.876740	-1.049118
12	1	0	-1.607812	-0.681336	-1.889936
13	8	0	-3.167067	-0.631168	-0.602484
14	8	0	1.545244	-0.198546	2.026030
15	6	0	-0.830669	-2.171634	0.312312
16	1	0	-0.451497	-2.675366	-0.580312
17	1	0	-0.228076	-2.489427	1.165252
18	1	0	-1.851183	-2.517933	0.479043
19	8	0	0.409852	1.324676	-0.238275
20	6	0	-3.907255	0.207039	0.163456

21	6	0	-5.282640	-0.366942	0.399111
22	1	0	-5.898643	0.364990	0.917325
23	1	0	-5.747443	-0.647377	-0.547978
24	1	0	-5.209535	-1.273832	1.004941
25	6	0	1.479932	2.173626	-0.333609
26	6	0	0.987534	3.586505	-0.504810
27	1	0	0.538158	3.697692	-1.495152
28	1	0	0.215074	3.818091	0.230279
29	1	0	1.823953	4.275820	-0.409633
30	8	0	-3.505995	1.258844	0.591730
31	8	0	2.625974	1.825266	-0.286227
32	6	0	2.603643	-0.593244	2.908636
33	1	0	2.670779	-1.683064	2.959994
34	1	0	3.552065	-0.174617	2.564844
35	1	0	2.345873	-0.189438	3.884310

Conformer **6cb**-4

Center Number	Atomic Number	Atomic Type	Coordinates (Angstroms) X	Y	Z
1	6	0	-1.925587	-0.406188	-0.037205
2	6	0	-0.456749	-0.124558	-0.325001
3	8	0	0.183807	-0.451305	0.897198
4	6	0	-0.745268	-0.943061	1.821212
5	6	0	-2.065841	-0.929466	1.188307
6	1	0	-2.956236	-1.237891	1.712489
7	8	0	-0.398479	-1.300990	2.907323
8	6	0	0.117162	-0.958542	-1.497592
9	1	0	-0.471759	-0.662530	-2.374074
10	6	0	1.569926	-0.581381	-1.823501
11	1	0	1.883527	-1.121168	-2.719670
12	1	0	1.681242	0.486789	-1.988142
13	8	0	2.463803	-0.996696	-0.774531
14	8	0	-2.786278	-0.126035	-1.008071
15	6	0	-0.038949	-2.466478	-1.275116
16	1	0	0.535299	-2.797408	-0.409509
17	1	0	-1.081365	-2.754902	-1.127518
18	1	0	0.331767	-3.010096	-2.147616
19	8	0	-0.190215	1.230233	-0.679519
20	6	0	3.049335	-0.033202	-0.015566
21	6	0	3.732467	-0.648552	1.174961
22	1	0	2.966886	-0.874534	1.922960
23	1	0	4.226398	-1.583973	0.909726
24	1	0	4.446272	0.056389	1.597263
25	6	0	-0.508681	2.264545	0.166520
26	6	0	0.194949	3.512535	-0.288758
27	1	0	1.272846	3.360682	-0.190969
28	1	0	-0.011675	3.708196	-1.343107
29	1	0	-0.124406	4.355731	0.320113
30	8	0	3.000306	1.145031	-0.262898
31	8	0	-1.230201	2.154298	1.116812

32	6	0	-4.171716	-0.347571	-0.715211
33	1	0	-4.351923	-1.404817	-0.503794
34	1	0	-4.476910	0.261740	0.138476
35	1	0	-4.718203	-0.046960	-1.605429

3. Experimental and calculated ECD spectra

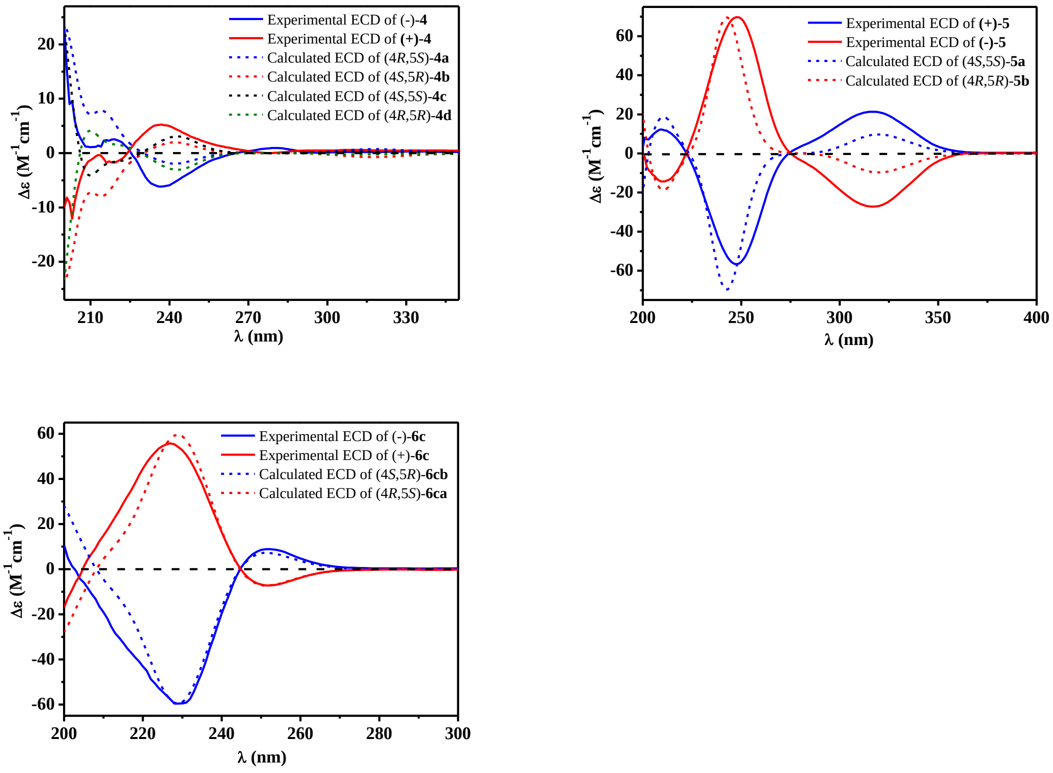

Figure AS2 Calculated ECD spectra of compounds **4**, **5**, and **6c** were compared with the experimental.
